# A network-based conditional genetic association analysis of the human metabolome

**DOI:** 10.1093/gigascience/giy137

**Published:** 2018-11-29

**Authors:** Y A Tsepilov, S Z Sharapov, O O Zaytseva, J Krumsek, C Prehn, J Adamski, G Kastenmüller, R Wang-Sattler, K Strauch, C Gieger, Y S Aulchenko

**Affiliations:** 1 Institute of Cytology and Genetics SB RAS, Novosibirsk, Lavrentieva Ave. 10, 630090, Russia; 2 Natural Scince Department, Novosibirsk State University, Novosibirsk, Pirogova Str. 1, 630090, Russia; 3 Institute of Computational Biology, Helmholtz Center Munich - German Research Center for Environmental Health, Neuherberg, Ingolstadter Landtrasse 1, 85764, Germany; 4 Institute of Experimental Genetics, Genome Analysis Center, Helmholtz Center Munich - German Research Center for Environmental Health, Neuherberg, Ingolstadter Landtrasse 1, 85764, Germany; 5 Institute of Experimental Genetics, Life and Food Science Center Weihenstephan, Technical University of Munich, Freising-Weihenstephan, Arcisstrasse 21, 80333, Germany; 6 German Center for Diabetes Research, Helmholtz Center Munich - German Research Center for Environmental Health, Neuherberg, Ingolstadter Landtrasse 1, 85764, Germany; 7 Institute of Bioinformatics and Systems Biology, Helmholtz Center Munich - German Research Center for Environmental Health, Neuherberg, Ingolstadter Landtrasse 1, 85764, Germany; 8 Research Unit of Molecular Epidemiology, Helmholtz Center Munich - German Research Center for Environmental Health, Neuherberg, Ingolstadter Landtrasse 1, 85764, Germany; 9 Institute of Epidemiology II, Helmholtz Center Munich - German Research Center for Environmental Health, Neuherberg, Ingolstadter Landtrasse 1, 85764, Germany; 10 Institute of Genetic Epidemiology, Helmholtz Center Munich - German Research Center for Environmental Health, Neuherberg, Ingolstadter Landtrasse 1, 85764, Germany; 11 Chair of Genetic Epidemiology, IBE, Faculty of Medicine, LMU Munich, Munich, Butenandstrasse 5, 81377, Germany; 12 PolyOmica, ’s-Hertogenbosch, Het Vlaggeschip 61, 5237 PA, The Netherlands

**Keywords:** genome-wide association study, multivariate model, metabolomics, conditional analysis, pleiotropy

## Abstract

**Background:**

Genome-wide association studies have identified hundreds of loci that influence a wide variety of complex human traits; however, little is known regarding the biological mechanism of action of these loci. The recent accumulation of functional genomics (“omics”), including metabolomics data, has created new opportunities for studying the functional role of specific changes in the genome. Functional genomic data are characterized by their high dimensionality, the presence of (strong) statistical dependency between traits, and, potentially, complex genetic control. Therefore, the analysis of such data requires specific statistical genetics methods.

**Results:**

To facilitate our understanding of the genetic control of omics phenotypes, we propose a trait-centered, network-based conditional genetic association (cGAS) approach for identifying the direct effects of genetic variants on omics-based traits. For each trait of interest, we selected from a biological network a set of other traits to be used as covariates in the cGAS. The network can be reconstructed either from biological pathway databases (a mechanistic approach) or directly from the data, using a Gaussian graphical model applied to the metabolome (a data-driven approach). We derived mathematical expressions that allow comparison of the power of univariate analyses with conditional genetic association analyses. We then tested our approach using data from a population-based Cooperative Health Research in the region of Augsburg (KORA) study (n = 1,784 subjects, 1.7 million single-nucleotide polymorphisms) with measured data for 151 metabolites.

**Conclusions:**

We found that compared to single-trait analysis, performing a genetic association analysis that includes biologically relevant covariates can either gain or lose power, depending on specific pleiotropic scenarios, for which we provide empirical examples. In the context of analyzed metabolomics data, the mechanistic network approach had more power compared to the data-driven approach. Nevertheless, we believe that our analysis shows that neither a prior-knowledge-only approach nor a phenotypic-data-only approach is optimal, and we discuss possibilities for improvement.

## Background

Genome-wide association studies (GWASs) are a highly popular method for identifying alleles that affect complex traits in humans, including the risk of common diseases. In the past decade, GWASs have enabled the identification of thousands of loci, significantly increasing our understanding of the genetic basis underlying the control of complex human traits [1]. On the other hand, this has had only a limited impact on the development of biomarkers and therapeutic agents; in most cases, any association found using a GWAS approach can only serve as a starting point for future research, rather than providing a direct answer to the question of the genetic region's precise biological function. The recent accumulation of functional genomics (or “omics” for short) data, including information regarding the levels of gene expression (the transcriptome), metabolites (the metabolome), proteins (the proteome), and glycosylation (the glycome), can provide new insight into the functional role of specific changes in the genome [2, 3].

Metabolomics is an emerging field that has been studied extensively in the past decade. A number of GWASs of metabolites have been performed using various platforms [4–8], revealing literally dozens of loci associated with variations in various lipid species, amino acids, and other small molecules. Linking the variants that underlie these variations in metabolomics with various diseases can provide functional insight into the many disease-related associations that were reported in previous studies, including cardiovascular and kidney disease, type 2 diabetes, cancer, gout, venous thromboembolism, and Crohn's disease [5].

However, analyzing metabolomics data requires specialized statistical methods due to their characteristically high dimensionality and the presence of statistical dependencies that reflect biological relationships between different variables. Conventional univariate GWAS (uGAS) approaches ignore any possible dependencies between different omics traits, which can confound the biological interpretation of the results and may lead to a loss of statistical power. On the other hand, utilizing multivariate phenotype information increases the statistical power of the association tests compared to univariate analysis [9–12]. Despite a large number of methodological studies, only a few empirical multivariate GWASs have been published using data for humans. We recently demonstrated [13] that using a multivariate analysis can substantially increase the power of locus identification in the context of human *N*-glycomics. Indeed, not only did our multivariate analysis double the number of loci identified in the analysis sample but also all five novel loci were strongly replicated. With respect to metabolomics, Inouye et al. [6] performed a multivariate GWAS on 130 metabolites (grouped in 11 sets) measured in approximately 6,600 individuals. They found that multivariate analysis doubled the number of loci detected in this sample; seven of these additional loci discovered were novel loci that had not been identified previously in other GWAS analyses of related traits. While no replication of novel loci was performed by Inouye et al., we compared the authors’ results with a recently published univariate GWAS of metabolomics derived from a cohort containing nearly 25,000 individuals [8]. We found that three of the seven single-nucleotide polymorphisms (SNPs) reported by Inouye et al. have a *P*-value < 5 × 10^−11^ for at least one metabolite (i.e., are significant at the genome-wide level after Bonferroni correction for 130 analyses). These findings provide empirical evidence supporting the value of using multivariate methods to analyze the genomics of metabolic traits, at least in the context of locus discovery.

It should be noted that these multivariate methods and tests were developed by statistical geneticists to specifically increase the power of gene identification. In such “gene-centric” tests, the model that includes the effects of genotype on multiple traits is contrasted with the null model in which the gene has no effect on any trait analyzed. Although useful and powerful for genetic mapping, this approach may have limited interpretability in a context in which one is interested in the genetic control and biology of a specific trait or a subset of traits (the “trait-centered” view). Several statistical methods have been suggested to address the question of which specific traits are affected in an analyzed ensemble (see, e.g., [10, 14]). One such method is based on conditional analysis [15], in which a “target trait” is analyzed as a genotype-dependent variable and related traits are included in the regression model as covariates. Such a modeling approach allows, at least in theory, one to rule out indirect genetic effects (e.g., effects that are in fact solely mediated through some other trait) and study only the genetic effects that directly affect the trait of interest.

Here, we present a statistical model in which a given trait depends on a genetic polymorphism and in which a number of related traits are included in the model as covariates. In this model, the relationship between the genotype and the trait of interest is our primary focus. Analyzing such a model allows us to identify the direct effect of genetics on the trait of interest. Mathematically, the model is equivalent to the model used by Deng and Pan [15]. We first compare this conditional genetic association (cGAS) approach with the standard model in which a trait of interest depends solely on genotype, without other traits used as covariates (i.e., the univariate genetic association, or uGAS, model). We do so by mathematically deriving expressions that allow us to examine the relative power of the uGAS and cGAS approaches, and we identify the situations in which these models are expected to yield different results.

As might be expected, and as demonstrated here, the choice of covariates plays a critical role in conditional analyses. First, we use the assumption that the covariates (i.e., biologically relevant traits) are known. Second, we explore the problem of selecting appropriate covariates, and we test the approaches by performing a proof-of-principle study using metabolomics data consisting of 151 metabolites (Biocrates assay) obtained from the KORA (Cooperative Health Research in the region of Augsburg) F4 study (n = 1,785 individuals). Specifically, we select covariates based on existing knowledge from metabolite biochemical networks (BN-cGAS) and use a data-driven approach based on Gaussian graphical modeling (GGM-cGAS). Finally, we compare and discuss the obtained results, and we discuss possible applications for this analysis based on biologically and/or statistically relevant traits.

## Results

### The power of performing a conditional analysis of genetic associations

We start with the theoretical substantiation and identification of specific scenarios in which adjusting for biologically relevant covariates can modify the power of an association analysis.

Let us consider a trait of interest *y*, covariate *c*, and genotype *g*. We can formulate this problem in terms of a linear regression as follows: $y = \mu + {\beta _g}*g + {\beta _c}*c + e$, where ${\beta _g}$ and ${\beta _c}$ are the effects of the genotype and covariate, respectively, and $e$ is the residual noise. Without a loss of generality, we assume that all random variables in this equation are distributed with a mean of zero and a standard deviation of 1, making (partial) regression coefficients equal to (partial) correlation coefficients. Given these assumptions, the joint distribution of *y, g*, and *c* can be specified using a set of three correlation coefficients, ${\rho _{yg}}$ (the correlation between the trait and the genotype), ${\rho _{cg}}$ (the correlation between the covariate and the genotype), and ${\rho _{yc}}$ (the correlation between the trait and the covariate). To test the association between *y* and *g*, we use the Wald test, which is defined as the square of the ratio between the effect estimate and its standard error, with the latter estimated under the alternative hypothesis (see [16]). The value of the “univariate” Wald test statistic is calculated as $T_u^2 = \frac{{n\ \hat{\rho }_{yg}^2}}{{\hat{\sigma }_u^2}}\ $, where *n* is the sample size and $\hat{\sigma }_u^2 = 1 - \hat{\rho }_{yg}^2$ is the estimated residual variance of *y*. For the conditional test, the Wald test is $T_c^2 = \frac{{n\ \hat{\beta }_g^2}}{{\hat{\sigma }_c^2}}\ $, where $\ {\hat{\beta }_g}$ is the estimated partial correlation between the trait *y* and the genotype *g* (estimated from the conditional model) and $\hat{\sigma }_c^2$ is the estimated residual variance of *y*. Note that under the null hypothesis, when *n* is large, both $T_u^2$ and $T_c^2$ are well approximated by chi-square distribution with one degree of freedom. For genetic association studies, *n* is thousands or orders of magnitude more.

For the conditional model, $\ {\hat{\beta }_g} = {\hat{\rho }_{yg}}\ - {\hat{\beta }_c}{\hat{\rho }_{cg}}$; thus, we can rewrite $T_c^2 = n{( {{{\hat{\rho }}_{yg}} - {{\hat{\beta }}_c}{{\hat{\rho }}_{cg}}} )^2}/\hat{\sigma }_c^2$. Consequently, the log-ratio of the conditional and univariate test statistics can be partitioned into two components:

(1) \begin{equation*} \log \left( {\frac{{T_c^2}}{{T_u^2}}} \right) = {\rm{log}}\left( {\frac{{\hat{\sigma }_u^2}}{{\hat{\sigma }_c^2}}} \right) + {\rm{log}}\left( {{{\left[ {1 - \frac{{{{\hat{\beta }}_c}{{\hat{\rho }}_{cg}}}}{{{{\hat{\rho }}_{yg}}}}} \right]}^2}} \right) \end{equation*}

Because the first term in Equation (1) is dependent only upon residual variances of the two models, we call this term the “noise” component. The second term depends upon the correlations between traits and between the traits and the genotype; we call this term the “pleiotropic” component. Because the noise component ($\hat{\sigma }_u^2$/$\hat{\sigma }_c^2$) is always ≥1, any possible decrease in the ratio between univariate and conditional tests is determined by the sign and the magnitude of the term ${\hat{\beta }_c}{\hat{\rho }_{cg}}/{\hat{\rho }_{yg}}$. If this term is negative, there will always be an increase in the power of the conditional analysis.

We can re-write ${\hat{\beta }_c}{\hat{\rho }_{cg}}/{\hat{\rho }_{yg}}$ as ${\hat{\beta }_c}\hat{\rho }_{yc}^*$, where $\hat{\rho }_{yc}^* = {\hat{\rho }_{cg}}/{\hat{\rho }_{yg}}$ is the component of the correlation between trait *y* and covariate *c*, which is induced by the variation in the genotype *g*. This quantity takes a central place in a Mendelian randomization analysis, which uses a genetic variation to anchor the causality arrow and consequently infers a causal relation between various traits (see, e.g., [17]). Note that whereas $\hat{\rho }_{yc}^*$ reflects the covariance between the trait and the covariate induced by the effect of the genotype, ${\hat{\beta }_c}$ is conditional on the genotype and is related to the residual sources of covariance between *y* and *c*.

In general, the genetically induced covariance and the residual covariance are expected to have a concordant sign (see Discussion for details and relevant references). Thus, we conclude somewhat surprisingly that when genotype-induced and environmental correlations are similar in sign (i.e., both are positive or both are negative), the product ${\hat{\beta }_c}\hat{\rho }_{yc}^*$ is positive and the contribution of the second term in Equation (1) to the relative power is negative. Note that the contribution of the first term in Equation (1) is always positive; therefore, even if ${\hat{\beta }_c}\hat{\rho }_{yc}^*$ is positive, the power of a conditional analysis may still be higher than the power of a univariate analysis. In contrast, an “unexpected” product (in which the signs are different and hence ${\hat{\beta }_c}\hat{\rho }_{yc}^*$ is negative) contributes positively to the relative power of the conditional model. Note that in such a situation, the power of a conditional analysis will always be higher than the power of a univariate analysis.

We can readily extend Equation (1) to a situation in which *k* covariates are included in the conditional model. Denoting the estimated coefficients of correlation between *g* and covariate *i* as ${\hat{\rho }_{gi}}$ and the estimated partial correlation between *y* and covariate *i* as ${\hat{\beta }_i}$ yields the following equation:

(2) \begin{equation*} \log \left( {\frac{{T_c^2}}{{T_u^2}}} \right) = {\rm{log}}\left( {\frac{{\hat{\sigma }_u^2}}{{\hat{\sigma }_c^2}}} \right) + {\rm{log}}\left( {{{\left[ {1 - \frac{1}{{{{\hat{\rho }}_{yg}}}}\mathop \sum \limits_{i = 1}^k {{\hat{\beta }}_i}{{\hat{\rho }}_{gi}}} \right]}^2}} \right) \end{equation*}

When appropriate covariates are selected, performing cGAS using individual-level data becomes rather trivial and can be achieved using standard statistical and software tools in which one estimates the effects of a SNP and covariates. However, cGAS becomes somewhat less trivial if one chooses to use summary-level univariate GWAS data such as data available from previously published studies. The formalization of cGAS in terms of summary univariate GWAS statistics is described in Supplementary Note 1. Here, we used methods based on analyzing summary-level data.

### Network-based selection of covariates

The ability to select appropriate covariates is extremely important, as it can have direct implications regarding the outcome of the analysis. If the biological/biochemical relationships between traits of interest are known and are summarized in a database(s), this knowledge can be used directly, e.g., by using all direct neighbors as covariates. We refer to this approach as a biochemical network-driven cGAS (BN-cGAS). Alternatively, the network can be reconstructed in a hypothesis-free, empirical manner from the data, e.g., using a GGM [18]. We refer to this approach as a GGM-cGAS.

We compared cGAS and uGAS by performing a genome-wide analysis of genetic effects using summary-level data obtained from the KORA F4 study. This study included 151 metabolites measured in 1,784 individuals using the Biocrates assay and imputed at 1,717,498 SNPs.

First, we examined the potential of using cGAS when the covariates are selected based on a known biochemical network (i.e., BN-cGAS). Thus, our analysis was restricted to a subset of 105 metabolites for which at least the one-reaction-step immediate biochemical neighbors are known [18]. This biochemical network incorporates only lipid metabolites, and the pathway reactions cover two groups of pathways: fatty acid biosynthesis reactions, which apply to the metabolite classes lyso-PC, diacyl-PC, acyl-alkyl-PC, and sphingomyelins, and β-oxidation reactions that reflect fatty acid degradation and apply to acylcarnitines. The β-oxidation model consists of a linear chain of C2 degradation steps (C10 to C8 to C6, etc.). The number of covariates ranged from 1 to 4, with mean and median values of 2.48 covariates and 2 covariates, respectively.

Table 1 lists the 11 loci that were significant in either BN-cGAS or uGAS and fell into known associated regions (see Supplementary Note 2). Of these 11 loci, 10 and 9 loci could be identified by BN-cGAS and uGAS, respectively. Compared to uGAS, BN-cGAS identified one fewer locus (*ETFDH*) but identified two more (*ACSL1* for PC ae C42:5 and *PKD2L1* for lyso-PC a C16:1). It is interesting to note that for *ACSL1*, the effect of SNP rs4862429 on PC ae C42:5 was highly significant (*P* = 7e-11) with BN-cGAS but was not significant (*P* = 0.7) with uGAS; this outcome is to be expected under the model of unexpected pleiotropy.

**Table 1:** Eleven loci identified by BN-cGAS and uGAS on metabolites for which at least one one-reaction-step neighbor was available

							uGAS		cGAS				
Locus	SNP	Metabolite	chr:pos	Gene	effA/refA	EAF	Beta (se)	*P*- value GC	Beta (se)	*P*- value GC	N_cov_	Noise	Pleiotropic
***uGAS and cGAS***													
1	rs211718	C8	1:75879263	*ACADM*	T/C	0.3	−0.45 (0.034)	6.35E-39	−0.10 (0.012)	4.45E-17	1	0.92	−1.29
1	rs211718	C12	1:75879263	*ACADM*	T/C	0.3	−0.04 (0.036)	2.21E-01	0.20 (0.014)	4.07E-42	3	0.80	1.29
2	rs7705189	PC ae C42:5	5:131651257	*SLC22A4*	G/A	0.47	0.15 (0.034)	8.83E-06	0.06 (0.009)	9.63E-11	3	1.16	−0.83
2	rs419291	C5	5:131661254	*SLC22A4*	T/C	0.38	0.26 (0.035)	6.62E-14	0.17 (0.029)	1.40E-08	1	0.16	−0.40
3	rs9368564	PC aa C42:5	6:11168269	*ELOVL2*	G/A	0.25	−0.29 (0.039)	4.64E-14	−0.15 (0.024)	1.06E-10	3	0.45	−0.58
4	rs12356193	C0	10:61083359	*SLC16A9*	G/A	0.17	−0.51 (0.046)	4.93E-28	−0.42 (0.042)	8.83E-23	1	0.07	−0.17
5	rs174547	lyso-PC a C20:4	11:61327359	*FADS1*	C/T	0.7	0.61 (0.033)	2.12E-75	0.66 (0.024)	2.65E-169	1	0.29	0.07
6	rs2066938	C4	12:119644998	*ACADS*	G/A	0.27	0.73 (0.033)	1.07E-104	0.72 (0.031)	4.26E-116	1	0.05	0.00
7	rs10873201	PC ae C36:5	14:67036352	*PLEKHH1*	T/C	0.45	−0.26 (0.034)	6.34E-14	−0.21 (0.018)	5.72E-31	2	0.55	−0.18
7	rs1077989	PC ae C32:2	14:67045575	*PLEKHH1*	C/A	0.46	−0.30 (0.034)	9.22E-19	−0.06 (0.016)	5.39E-05	3	0.66	−1.34
8	rs4814176	PC ae C40:2	20:12907398	*SPTLC3*	T/C	0.36	0.24 (0.035)	5.60E-12	0.25 (0.023)	1.28E-25	4	0.35	0.02
***Only uGAS***													
9	rs8396	C10	4:159850267	*ETFDH*	C/T	0.71	0.26 (0.037)	1.32E-12	0.05 (0.010)	5.08E-07	2	1.09	−1.39
***Only cGAS***													
10	rs4862429	PC ae C42:5	4:186006834	*ACSL1*	T/C	0.31	0.02 (0.037)	6.63E-01	−0.06 (0.010)	7.01E-11	3	1.15	1.20
11	rs603424	Lyso-PC a C16:1	10:102065469	*PKD2L1*	A/G	0.8	0.23 (0.042)	4.83E-08	0.21 (0.031)	1.76E-11	1	0.26	−0.07

Notes: The best SNP-metabolite pair is shown for each locus. chr: pos, the physical position of the SNP; EAF, effect allele frequency; beta (se), the estimated effect and standard error of the SNP; effA/refA, effect allele/reference allele; *P*-value, the *P*-value of the additive model; Gene, the most likely (according to Data-driven Expression-Prioritized Integration for Complex Traits [DEPICT] software) associated gene in the region; N_cov_, the number of covariates used in cGAS; Noise/Pleiotropic, the values of noise and pleiotropic components of the log-ratio of cGAS and uGAS T^2^ statistics.

Next, to test whether using BN-cGAS increases the average power of the association analysis, we compared the BN-cGAS and uGAS chi-square test results for the loci listed in Table 1. Within a given locus, we compared the maximum test value. The average ratio of the maximum test statistic between BN-cGAS and uGAS was 1.47, indicating that, on average, BN-cGAS led to higher test statistic values. However, when we used a paired-sample Wilcoxon test to compare the best chi-square test results between BN-cGAS and uGAS, the difference between the two methods was not significant (*P* = 0.123) (see Supplementary Table S1A).

**Figure 1:**
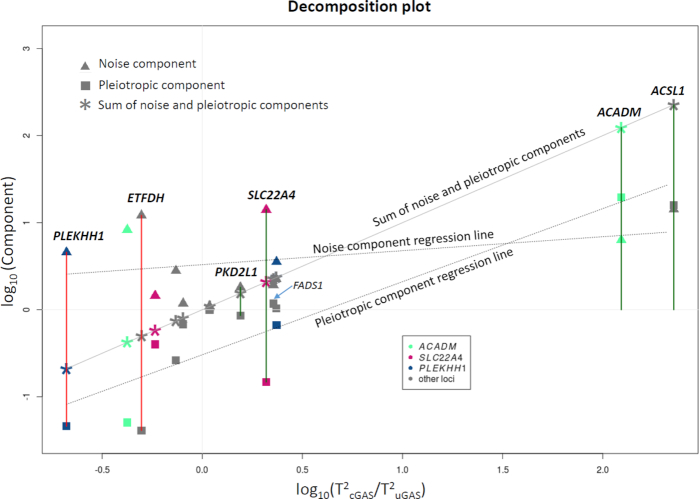
Decomposition of the log-T^2^ ratio for cGAS and uGAS into pleiotropic and noise components. Vertically grouped trios (each composed of a square, triangle, and asterisk) correspond to 1 of 14 associations in Table 1. The position of a trio on the *x*-axis corresponds to the log-ratio between conditional and univariate test statistic. On the *y*-axis, the asterisk corresponds to the log-ratio of cGAS and uGAS T^2^ statistics. The value of the pleiotropic component is depicted by a square, and the value of the noise component is depicted by a triangle. Each trio is shown in gray, except the trios representing the *ACADM, SLC22A4*, and *PLEKHH1* loci, for which we have two different associations. The three dotted lines correspond to the regression lines for the two components and their sum. The four dark-green vertical lines indicate the associations that were significant in the cGAS analysis but not in the uGAS analysis, and the two dark-red lines indicate the associations that were significant only in the uGAS analysis.

For the SNPs listed in Table 1, we then used Equation (2) to partition the log-ratio of the BN-cGAS and uGAS statistics values into “noise” and “pleiotropic” components. As shown in Fig. 1, the regression slope of the second (i.e., “pleiotropic”) component is considerably higher than the slope of the noise component; in other words, the ratio is determined primarily by the pleiotropic term in Equation (2). Moreover, with the exception of the *SLC22A4* locus, the SNP-trait pairs for which BN-cGAS had increased power are the pairs in which the second term in Equation (2) is either positive or close to zero. In contrast, in the SNP-trait pairs that were not identified using BN-cGAS, the “pleiotropic” term in Equation (2) had a strong negative contribution.

**Figure 2:**
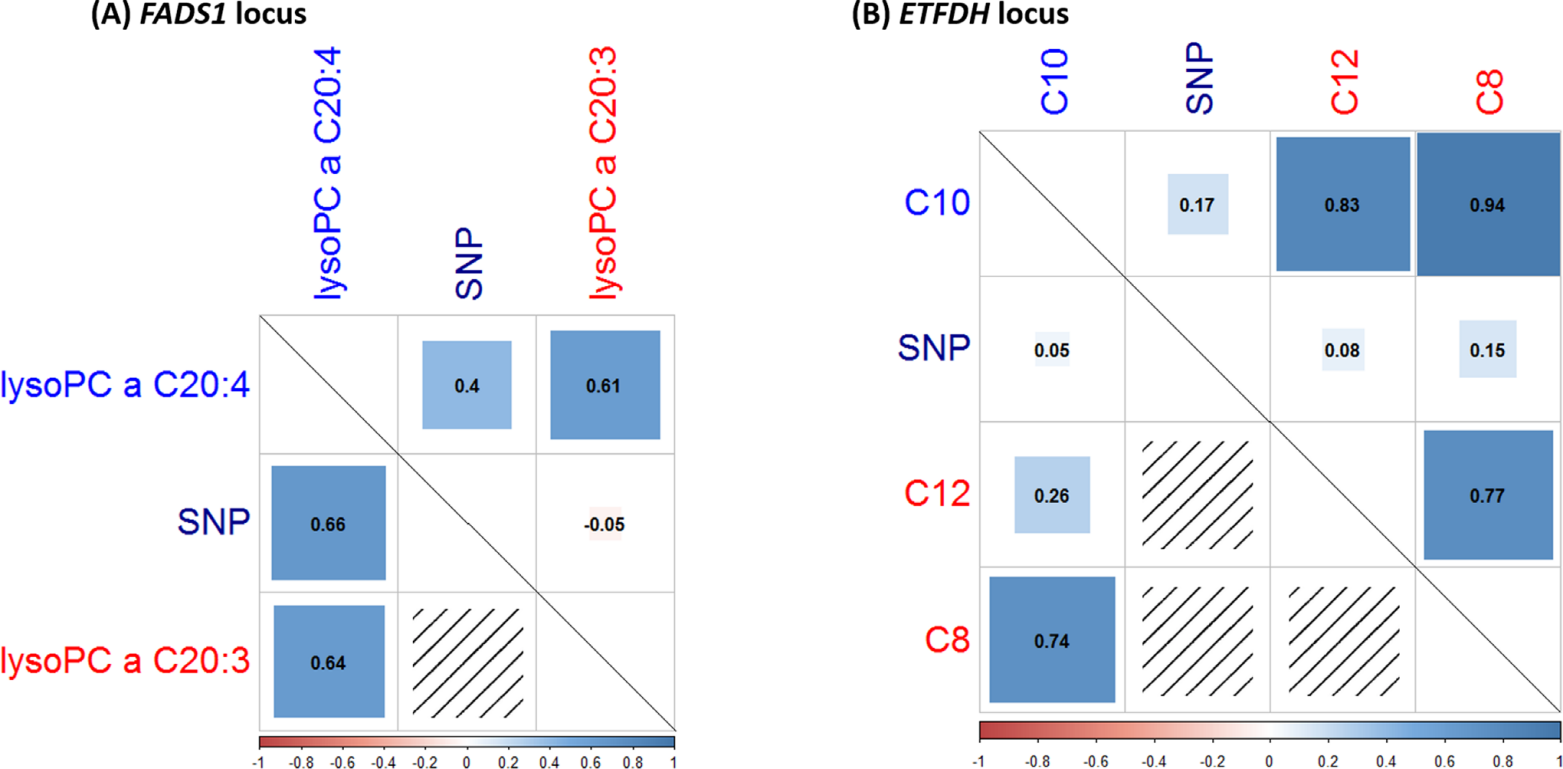
Matrix of correlations (above diagonal line) and the partial regression coefficients of the trait of interest on the SNP genotype and covariate(s) (the first column) for the *FADS1***(A)** and *ETFDH***(B)** loci. The result of the univariate analysis of regression of the corresponding traits onto SNPs is presented in Supplementary Table S1C. Names of traits used as covariates are in red. The number in a cell indicates the value of correlation (partial regression coefficient). The area of a square is proportional to the absolute value of correlation (partial regression coefficient); the effect magnitude is also reflected by a square's color (the scale provided at the bottom of the graph). The *FADS1* locus represents scenario in which the pleiotropic term in Equation (2) is strongly positive, while for *ETFDH* this term is negative.

Next, we investigated the variance-covariance structure of the loci with positive and negative pleiotropic terms. We therefore selected a locus in which the pleiotropic component's contribution to power was positive (rs174547 at *FADS1*) and a locus in which the pleiotropic component's contribution to power was negative (rs8396 at *ETFDH*). Figure 2 shows the corresponding correlations between the SNP, the trait, and the covariates involved, together with the partial coefficients for the conditional regression of the trait on the SNP and the covariates. With respect to *FADS1* (Fig. 2A), the correlations between the SNP and the trait (lyso-PC a C20:4) and between the SNP and the covariate (lyso-PC a C20:3) are in opposite directions, generating negative genetically induced covariance between lyso-PC a C20:4 and lyso-PC a C20:3. In contrast, the residual correlation between the trait and the covariate is positive. Therefore, the value of the partial regression coefficient between the SNP and lyso-PC a C20:4, conditional on lyso-PC a C20:3, is greater than that of the coefficient of regression without covariates.

With respect to the second example, *ETFDH* (Fig. 2B), we found that the conditional regression of C10 on rs8396 and two covariates (C8 and C12, two medium-chain acylcarnitines) led to a smaller SNP partial regression coefficient compared to an unconditional regression; this is because all of the terms in $\mathop \sum \limits_{i = 1}^k {\hat{\beta }_i}{\hat{\rho }_{gi}}/{\hat{\rho }_{yg}}$ are positive.

Although using a known biochemical network to select covariates has many advantages, it may be somewhat impractical and perhaps even harmful, as our biochemical knowledge is still relatively incomplete. Therefore, we explored the potential of performing a cGAS in which the covariates are selected using a data-driven approach (GGM-cGAS). The network of metabolites was reconstructed using GGMs based on partial correlations. For a given metabolite, we selected covariates based on significant partial correlations. Specifically, we used the following threshold as proposed previously [18]: a *P*-value ≤ (0.01/number of calculated partial correlations), which corresponds to a cutoff at *p* ≤ 8.83 × 10^−7^. The network used in our analysis is shown in Supplementary Fig. S1.

To compare GGM-cGAS with BN-cGAS, we used the same set of metabolites that we used for BN-cGAS to run our GGM-cGAS analysis; these results are presented in Supplementary Table S1B. We found 15 SNP-trait pairs clustered at 10 known loci (see Supplementary Note 2) that were detected by either GGM-cGAS or BN-cGAS. More covariates were included in the GGM-cGAS analysis (ranging from 1 to 18, with mean and median values of 7.6 covariates and 7 covariates, respectively) than in the BN-cGAS analysis. Thus, we predicted that GGM-cGAS would have relatively more power than BN-cGAS due to reduced noise (term 1 in Equation [2]); on the other hand, GGM-cGAS might lose power because of reduced occurrence of unexpected pleiotropy (term 2 in Equation [2]).

For the best SNP-trait pairs detected by GGM-cGAS or BN-cGAS, we computed the components in Equation (2) and compared these components using a paired-sample Wilcoxon test. We found that the noise component in Equation (2) was always larger for GGM-cGAS, with a mean difference of 0.29 (*P* = 6 × 10^−5^). Moreover, the second “pleiotropic” component in Equation (2) was generally smaller for GGM-cGAS than for BN-cGAS, with a mean difference of −0.47 (*P* = 0.015). Nevertheless, for 3 of 15 GGM-cGAS SNP-trait pairs, the pleiotropic component was positive. The average chi-square value was 25% smaller for GGM-cGAS than for BN-cGAS, indicating an average loss of power (although this loss was not significant; *P* = 0.5 based on a paired Wilcoxon test).

Next, we investigated further the potential of using cGAS under realistic conditions to a full extent by analyzing all 151 available metabolites using GGM-cGAS and comparing these results with the results of uGAS (Table 2 and Supplementary Fig. S2). In total, uGAS detected 15 loci at the genome-wide significance level *p* ≤ 5 × 10^−8^/151 (i.e., *P* < 3.3 × 10^−10^). On the other hand, GGM-cGAS identified 19 significant loci using the same threshold. As expected, the standard errors of the genetic effect estimates were smaller for GGM-cGAS than for uGAS (Table 2 and Supplementary Fig. S3). A total of 14 loci were detected by both uGAS and GGM-cGAS. GGM-cGAS failed to identify one locus that was identified by uGAS (C5:1-DC at rs2943644) but identified five loci that were missed by uGAS. Three of the five loci identified solely by GGM-cGAS affect amino acids, and the remaining two loci affect acylcarnitines. It is important to note that the loci identified by BN-cGAS (when we analyzed 105 metabolites) are a subset of the 19 loci that were identified by GGM-cGAS (when we used all 151 metabolites).

**Table 2:** Twenty loci identified by GGM-cGAS and uGAS

							uGAS		cGAS				
Locus	SNP	Metabolite	chr:pos	Gene	effA/refA	EAF	Beta (se)	*P-*value GC	Beta (se)	*P*- value GC	N_cov_	Noise	Pleiotropic
***uGAS and cGAS***													
1	rs211718	C6 (C4:1-DC)	1:75879263	*ACADM*	T/C	0.30	−0.48 (0.034)	3.31E-44	−0.13 (0.017)	1.21E-13	7	0.61	−1.16
1	rs7552404	C6 (C4:1-DC)	1:75908534	*ACADM*	G/A	0.30	−0.48 (0.034)	2.14E-44	−0.12 (0.017)	2.34E-13	7	0.61	−1.17
2	rs483180	Ser	1:120069028	*PHGDH*	G/C	0.30	−0.24 (0.037)	2.26E-11	−0.24 (0.028)	1.10E-17	2	0.24	−0.02
2	rs477992	Ser	1:120059099	*PHGDH*	A/G	0.70	0.24 (0.037)	3.50E-11	0.24 (0.028)	2.52E-18	2	0.24	0.00
3	rs2286963	C9	2:210768295	*ACADL*	G/T	0.63	−0.49 (0.032)	4.76E-52	−0.48 (0.027)	7.41E-73	3	0.16/	−0.01
4	rs8396	C10	4:159850267	*ETFDH*	C/T	0.71	0.26 (0.037)	1.32E-12	0.04 (0.010)	1.23E-05	8	1.11	−1.53
4	rs8396	C7-DC	4:159850267	*ETFDH*	C/T	0.71	−0.09 (0.037)	1.67E-02	−0.13 (0.019)	2.93E-11	8	0.56	0.33
5	rs419291	C5	5:131661254	*SLC22A4*	T/C	0.38	0.26 (0.035)	6.62E-14	0.17 (0.026)	2.25E-10	3	0.25	−0.40
5	rs270613	C5	5:131668482	*SLC22A4*	A/G	0.61	−0.26 (0.035)	7.48E-14	−0.17 (0.026)	8.24E-11	3	0.25	−0.38
6	rs9393903	PC aa C42:5	6:11150895	*ELOVL2*	A/G	0.75	0.29 (0.039)	9.13E-14	0.18 (0.020)	1.32E-19	6	0.56	−0.38
6	rs9368564	PC aa C42:5	6:11168269	*ELOVL2*	G/A	0.25	−0.29 (0.039)	4.64E-14	−0.19 (0.021)	3.04E-19	6	0.56	−0.40
7	rs816411	Ser	7:56138983	*PHKG1*	C/T	0.51	−0.22 (0.034)	1.53E-10	−0.19 (0.026)	4.83E-13	2	0.23	−0.12
7	rs1894832	Ser	7:56144740	*PHKG1*	C/T	0.51	0.21 (0.034)	2.33E-10	0.19 (0.026)	1.55E-13	2	0.23	−0.09
8	rs12356193	C0	10:61083359	*SLC16A9*	G/A	0.17	−0.51 (0.046)	4.93E-28	−0.27 (0.034)	1.03E-15	3	0.26	−0.53
9	rs174547	lyso-PC a C20:4	11:61327359	*FADS1*	C/T	0.70	0.61 (0.033)	2.12E-75	0.07 (0.011)	2.08E-10	9	0.98	−1.90
9	rs174556	PC ae C44:4	11:61337211	*FADS1*	T/C	0.27	0.09 (0.038)	1.61E-02	0.21 (0.014)	1.17E-48	3	0.84	0.73
10	rs2066938	C4	12:119644998	*ACADS*	G/A	0.27	0.73 (0.033)	1.07E-104	0.71 (0.024)	6.95E-189	2	0.28	−0.02
11	rs12879147	PC aa C28:1	14:63297349	*SYNE2*	A/G	0.85	−0.46 (0.050)	1.83E-19	−0.12 (0.019)	6.87E-11	14	0.86	−1.14
11	rs17101394	SM(OH) C14:1	14:63302139	*SYNE2*	A/G	0.83	−0.32 (0.050)	1.02E-10	−0.10 (0.011)	9.23E-18	7	1.30	−1.05
12	rs1077989	PC ae C36:5	14:67045575	*PLEKHH1*	C/A	0.46	−0.26 (0.034)	4.96E-14	−0.08 (0.010)	2.56E-15	10	1.05	−1.00
12	rs1077989	PC ae C32.2	14:67045575	*PLEKHH1*	C/A	0.46	−0.30 (0.034)	9.22E-19	−0.05 (0.016)	1.35E-03	6	0.67	−1.55
13	rs4814176	SM(OH).C22:1	20:12907398	*SPTLC3*	T/C	0.36	0.03 (0.035)	4.53E-01	−0.07 (0.009)	9.11E-17	10	1.22	0.87
13	rs4814176	SM(OH) C24:1	20:12907398	*SPTLC3*	T/C	0.36	0.24 (0.035)	4.29E-12	0.09 (0.013)	2.85E-11	9	0.86	−0.90
14	rs5746636	Pro	22:17276301	*PRODH*	T/G	0.24	−0.31 (0.039)	1.89E-15	−0.32 (0.034)	5.05E-21	2	0.11	0.03
***Only uGAS***													
15	rs2943644	C5:1-DC	2:226754586	*LOC646736*	C/T	0.68	0.32 (0.042)	3.99E-14	0.09 (0.022)	3.97E-05	5	0.56	−1.08
***Only cGAS***													
16	rs1374804	Gly	3:127391188	*ALDH1L1*	A/G	0.64	0.20 (0.036)	1.46E-08	0.21 (0.029)	3.65E-13	3	0.17	0.05
17	rs4862429	PC ae C42:5	4:186006834	*ACSL1*	T/C	0.31	0.02 (0.037)	6.63E-01	−0.06 (0.008)	1.15E-12	8	1.34	1.09
18	rs603424	C16:1	10:102065469	*PKD2L1*	A/G	0.80	0.16 (0.042)	9.00E-05	0.14 (0.018)	9.32E-14	9	0.71	−0.15
19	rs2657879	Gln	12:55151605	*GLS2*	G/A	0.21	−0.24 (0.042)	2.65E-08	−0.27 (0.030)	5.88E-18	5	0.29	0.10
20	rs17112944	C6:1	14:27179297	*LOC728755*	A/G	0.90	−0.28 (0.059)	1.98E-06	−0.21 (0.031)	3.74E-11	9	0.54	−0.26

Notes: The best SNP-metabolite pair is shown for each locus. chr: pos, the physical position of the SNP; EAF, effect allele frequency; beta (se), the estimated effect and standard error of the SNP; effA/refA, effect allele/reference allele; *P*-value, the *P*-value of the additive model; Gene, the most likely (according to Data-driven Expression-Prioritized Integration for Complex Traits [DEPICT] software) associated gene in the region; N_cov_, the number of covariates used in cGAS; Noise/Pleiotropic, the values of noise and pleiotropic components of the log-ratio of cGAS and uGAS T^2^ statistics.

Finally, we searched the available literature for the loci listed in Table 2 (see Supplementary Note 2 for details). From the 20 loci that we report here, 15 were found to be significant at the genome-wide level in a recent large (n = 7,478) meta-analysis of Biocrates metabolomics data reported by Draisma et al. [7]. Some of the metabolites analyzed in our study were not analyzed by Draisma et al. [7]. Nevertheless, for 11 of these 15 loci, we observed a significant association for the same SNP-metabolite pair; for 3 loci, the strongest association was with a metabolite in the same class; and for 1 locus, the strongest association was with a metabolite from a different lipid class (see Supplementary Table S2). For the other five loci that were not significant in the study by Draisma et al. [7], we determined whether these five loci were significant and replicated in a study by Tsepilov et al. [19]. It should be noted that Tsepilov et al. analyzed the ratios of metabolites and also used the KORA F4 dataset in their discovery stage, although they used another cohort (TwinsUK) for replication. Of these five loci, two were also significant in the study by Tsepilov et al. [19]. Moreover, for both of these loci, the metabolite analyzed in our study was included in the ratios analyzed by Tsepilov et al. One of the five loci was associated with the same trait in two other studies [20, 21]. Finally, we found no prior published evidence of any association with metabolites for rs2943644 (*LOC646736*) or rs17112944 (*LOC728755*). Taking into account that this association was not found in (much) bigger meta-analysis, we conclude the observed associations with rs17112944 and rs2943644 as likely false positives, and these two loci were excluded from further consideration.

## Discussion

We report a new “trait-centric” approach for analyzing genetic determinants of multivariate “omics” traits by performing a network-based cGAS analysis. In the context of metabolomics, for each trait we selected a set of other metabolites to be used as covariates in our genetic association analysis. The selection of covariates can be either mechanistic (e.g., based on known biological relationships between traits of interest) or data driven (e.g., based on partial correlations). Importantly, this approach can use either individual-level or summary-level data. First, we mathematically compared the power of conditional and standard single-trait genetic association analyses (uGAS), and we identified scenarios in which these analyses are expected to produce different results. Next, we applied cGAS to 151 metabolomics traits (Biocrates panel) in a large (n = 1,784 individuals) population-based KORA cohort.

We found that the log-ratio between the cGAS and uGAS test statistic can be decomposed in a “noise” component (which depends on residual variance of the trait and is always positive) and a “pleiotropic” component. The pleiotropic component is negative in cases in which genetically induced covariance (between the trait of interest and the trait used as the covariate) and the residual covariance have the same sign (i.e., act in the same direction). The pleiotropic component is positive in cases in which the genetically induced covariance and residual covariance act in opposite directions.

Should one expect that genetically induced covariance and residual covariance act in the same or opposite directions? In essence, this is a question about the architecture of pleiotropy: is a pleiotropic genetic variant expected to induce the same covariance as would be induced by non-genetic mechanisms? It has been reasoned that in randomly bred populations, the genetic correlations are expected to arise primarily from pleiotropic gene action [22]. In such populations, a study and comparison of genetic and environmental correlations, while unable to provide single-variant resolution, may provide a general notion of what may be expected for consistency/anti-consistency between genetic and residual covariance. Based on published literature, Cheverud [23] and Roff [24] concluded that genetic and environmental correlations normally have both the same sign and the same magnitude. This pattern is particularly clear for morphologic traits, as opposed to life-history traits (see [25] for review and additional references). These observations are consistent with recent studies of genetic correlations between complex human polygenic traits (see [26]).

Consequently, for complex traits, one may expect that the sign of the pleiotropic component of the log-ratio between the cGAS and uGAS tests (individual summands in the second term of the Equation [2]) is generally negative. It should be noted, though, that a negative sign for the pleiotropic component does not necessarily indicate higher power of the uGAS, as the noise component (the first term in Equation [2]) may still dominate the log-ratio between the cGAS and uGAS tests. This will happen, e.g., when $\hat{\rho }_{cg}^{}$ (the effect of the genotype on the covariate) is small while $\hat{\beta }_{yc}^{}$ (partial residual regression between the trait and covariate) is relatively large, thereby reducing $\hat{\sigma }_c^2$.

Nevertheless, in the case of metabolomic traits, genetic and environmental sources do not necessarily generate consistent covariance. Moreover, for a given locus that affects the activity of an enzyme involved in a biochemical reaction, the unexpected inconsistency between genetically induced covariance and residual covariance may not be so unexpected after all. Indeed, consider an allele associated with an increased activity of an enzyme that converts substrate A into product B. One would expect that the levels of A and B are positively correlated. One would also expect that the allele is positively correlated with the level of product B and negatively correlated with the level of substrate A. This is precisely the scenario that yields a positive value for the second term in Equation (1), thus providing an additional increase in power above and beyond the power provided by the first term in Equation (1) (noise reduction).

Our empirical investigation of real data on the genetic association between the genome and metabolites confirmed the existence of both scenarios. An extreme example of concordance between genetic covariance and residual covariance is provided by the effects of rs8396 on C10, with C8 and C12 used as covariates (see Fig. 2B). The *ETFDH* gene, which was prioritized by Data-driven Expression-Prioritized Integration for Complex Traits (DEPICT) software (see Materials and Methods section) as the best candidate in this region (with a false-discovery rate <5%), encodes the enzyme electron transfer flavoprotein (ETF) dehydrogenase, which plays a role in mitochondrial fatty acid oxidation. During this process, the acyl group is transferred from a long-chain acylcarnitine to a long-chain acetyl-CoA, which is then catabolized. ETF dehydrogenase participates in the catabolic process by transferring electrons from acyl-CoA dehydrogenase to the oxidative phosphorylation pathway. Thus, the *ETFDH* gene should affect all forms of long-chain acylcarnitines in the same way, and we can expect that the pleotropic effect of this gene on the acylcarnitines in our example (C8, C10, C12, etc.) will be unidirectional. The presence of unidirectional genetic effects and the positive correlation between these acylcarnitines makes the second term in Equation (2) negative, which determines that, in this situation, uGAS has more power than cGAS.

An empirical example of discordance between genetically induced covariance and residual covariance is provided by the effects of the SNP rs174547 on lyso-PC a C20:4, with lyso-PC a C20:3 used as a covariate. This SNP exhibits opposite correlations with lyso-PC a C20:4 and lyso-PC a C20:3, resulting in negative genetically induced covariance between these traits. At the same time, the residual correlation between these traits is positive, resulting in a steep increase in the power of conditional analysis. In this region, the *FADS1/2/3* gene cluster is an attractive candidate, providing the detected model with biological relevance. The *FADS1* gene encodes the enzyme fatty acid desaturase 1, whereas the two traits differ by only one double bond. Thus, this example mimics perfectly the biochemical scenario in which we would expect a conditional analysis to have increased power.

The trait-centric methods considered here provide an attractive framework to identify and study direct genetic effects on a trait of interest. Conditional analysis is an attractive option in cases in which we wish to clearly interpret the results in terms of the effect of the genotype on a particular trait. Such specific interpretation may be important when comparing genetic association results obtained for our trait of interest with results obtained for other traits (e.g., using the methods described in [27–29]). It should be noted, though, that a trait-centric approach is not intended to maximize the power of identifying genes that affect metabolomics as a whole. Such a gene-centric view would favor analysis using joint, and not conditional, modeling of sets of traits. Such an approach can maintain power across a wide range of scenarios, including the scenario of concordance between genetically induced and residual covariance [13]. In this gene-centric framework, other formulations of conditional analysis have also been proposed [30] in order to specifically increase power of gene identification by selecting covariates that, using our terminology, affect the “noise reduction” component of the model while avoiding the problems associated with the pleiotropic component.

The proper selection of sets of biologically related traits is extremely important for the conditional genetic association analysis method described here, as well as for multivariate methods that model the joint effects of genotype on an ensemble of traits. Here, we considered two alternative approaches—knowledge based and data driven—to finding the networks of related traits, with a subnetwork centered around a trait of interest used as the analyzed set. In principle, in the context of analyzed metabolomics data, the knowledge-based network approach has slightly higher power in the context of trait-centric genetic association analysis. However, we believe that our analysis revealed that both approaches are suboptimal. The knowledge-based network reconstruction has many advantages, but it may be somewhat unpractical, as our biochemical knowledge is still relatively incomplete. Secondly, by reconstructing the network while relying only on current knowledge, we may be missing new knowledge that may be revealed by the data. Finally, by including neighbors that are based only on biochemical information, we may miss covariance induced by technical confounders; adjusting for this may increase the power of analysis [30]. Learning the network from the same data that were used for genetic analysis has the disadvantages of potentially ignoring existing knowledge and being sensitive to sample size. Finally, we note that the total observed correlation between metabolites is determined by the balance between genetic and environmental sources of covariance. It is possible to imagine a situation in which total correlation is smaller than one or more of its components, and our analysis provides examples of such a situation. We may speculate that, ideally, one should use a method that allows one to combine prior knowledge and new information obtained from the data, thereby allowing the simultaneous learning of the structure of dependencies between different metabolites and between the metabolites and the genome. Such learning from the data while allowing for the incorporation of previous knowledge (e.g., biochemical relations between traits) might be achieved (e.g., by applying a machine-learning approach that allows for differential shrinkage). It is also important to note that the proper application of such an approach would require the availability of vast samples of data, thereby allowing for separate training, validation, verification, testing, and replication of detected dependencies and associations.

## Materials and Methods

### KORA study

The KORA study (Cooperative Health Research in the region of Augsburg) is a series of population-based studies in the region of Augsburg in southern Germany [31]. KORA F4 is a follow-up survey (conducted from 2006 through 2008) of the baseline KORA S4 survey, which was conducted from 1999 through 2001. All study protocols were approved by the ethics committee of the Bavarian Medical Chamber, and all participants provided written informed consent.

The concentration of 163 metabolites were measured in 3,061 serum samples obtained from KORA F4 participants using flow injection electrospray ionization tandem mass spectrometry and the AbsoluteIDQ p150 Kit (Biocrates Life Sciences AG, Innsbruck, Austria) [32]. After applying quality-control screening, 151 metabolite measurements were used in our analysis. Details regarding the methods and quality control of the metabolite measurements, as well as details regarding the metabolite nomenclature, have been published previously [32]. The nomenclature for the metabolites in this study is provided in Supplementary Table S3.

Genotyping was performed using the Affymetrix 6.0 SNP array (534,174 SNP markers after quality control), with further imputation using HapMap2 (release 22) as a reference panel, resulting in 1,717,498 SNPs (for details, see ref. [33]). Both the metabolite concentrations and genotype were available for 1,785 participants in the KORA F4 study.

### Statistical analysis

Partial correlation coefficients and their *P*-values were calculated using the “ppcor” package [34] in R. Graphical representations were generated using the “ggm” [35] package in R. Consistent with previous studies [18], we considered a partial regression coefficient to be significant at *P* < 0.01/(151*150/2) (i.e., *P* < 8.83 × 10^−7^).

For the GWAS analysis, we used OmicABEL software [36]. Prior to GWAS, all traits were first adjusted for the participant's sex, age, and batch effect; subsequently, the residual traits were transformed using an inverse-normal transformation [37]. The genotypes from the KORA F4 cohort were used. Only SNPs that had a call rate ≥0.95, R^2^≥0.3, Hardy–Weinberg equilibrium p≥10^−6^, and , minor allele frequency (MAF) ≥0.1 (1,717,498 SNPs in total) were included in the analysis. The genomic control method was used to correct for any possible inflation of the test statistics. The genomic control [38] lambda value for all traits was between 1.00 and 1.03.

In a specific analysis (i.e., cGAS or uGAS), we defined independent loci as groups of genome-wide significant associations that were separated by at least 500 kb or were located on different chromosomes. The strongest association (i.e., the association with the lowest *P*-value) was selected to represent this locus. The cGAS and uGAS results were considered to reflect different loci if the strongest associations were in loci that were separated by at least 500 kb. The threshold for the genome-wide significance for 151 traits was set to *P* = 5 × 10^−8^/151 (i.e., *P* = 3.31 × 10^−10^).

When partitioning the log(cGAS/uGAS) test statistics into the noise and pleiotropic components (see Equation (2) and Fig. 1), we used all known loci that were significant in either the cGAS or uGAS analysis (see Table 1). If a locus included two SNPs associated with different traits, we included both associations during partitioning. If a locus included two SNPs associated with the same trait, to be conservative, we included only the SNP with the lower uGAS *P*-value during partitioning. After partitioning, we determined whether the value of the pleiotropic and noise components were statistically different using the paired-samples Wilcoxon test. For comparing the chi-square test results for the two methods, for each locus we selected the largest chi-square value for selected SNP among all analyzed traits. If a locus had two SNPs, we selected for each method only the largest chi-square value.

The code for BN-cGAS and GGM-cGAS analyses, and the code for producing the summary tables and graphs, was implemented in R and is available as a workflow from CodeOcean [39], a cloud-based computational reproducibility platform.

### *In silico* functional annotation

We conducted functional annotation for our findings. To prioritize genes in associated regions, gene set enrichment, and tissue/cell-type enrichment analyses, we used DEPICT software [40] (release 140721) with the following settings: flag_loci = 1; flag_genes = 1; flag_genesets = 1; flag_tissues = 1; param_ncores = 2; and further manual annotation (h37 assembly). All 27 SNPs (clustered in 20 loci) identified by cGAS or uGAS (see Table 2) were included in the analysis. If more than one gene was annotated for a SNP by DEPICT, we selected the gene with the lowest nominal DEPICT *P*-value. In most cases, the results of manual annotation matched the annotation results using DEPICT annotation (see Supplementary Note 2). In addition, we looked up each SNP using the Phenoscanner [41] database to check whether it was previously reported to be associated with metabolic traits at *P* < 5 × 10^−8^ and proxy r^2^ <0.7.

## Availability of supporting data

The code produced in relation to this work and all summary statistics and association data that are necessary to reproduce our results are stored in the GigaDB database [42] and on the CodeOcean platform as a reproducible workflow [39]. The informed consent given by the KORA study participants does not cover the posting of participant-level phenotype or genotype data in public databases. However, the KORA data are available upon request from KORA-gen (https://www.helmholtz-muenchen.de/en/kora/index.html). Requests can be submitted online and are subject to approval by the KORA board.

## Additional files

**Supplementary Note 1:** cGAS using summary level data

**Supplementary Note 2:** Literature search for loci identified by cGAS and uGAS

**Supplementary Table S1:** BN- cGAS and GGM- cGAS for 105 metabolites

**Supplementary Table S2:** GGM-cGAS and uGAS for 151 metabolites

**Supplementary Table S3:** List of metabolites measured using the AbsoluteIDQ p150 Kit

**Supplementary Figure S1:** Partial correlations network

**Supplementary Figure S2:** Manhattan plots for cGAS and uGAS for 151 metabolites

**Supplementary Figure S3:** Comparison of effect estimates and their standard errors for SNPs from Table 2

## Abbreviations

BN: biochemical network; BN-cGAS: cGAS based on biochemical networks; cGAS: conditional GWAS; DEPICT: Data-driven Expression-Prioritized Integration for Complex Traits; ETF: electron transfer flavoprotein; GGM-cGAS: Gaussian graphical modeling cGAS based on partial correlations network; GWAS: genome-wide association study; SNP: single-nucleotide polymorphism; uGAS: univariate GWAS (trait-by-trait); KORA study: Cooperative Health Research in the region of Augsburg; MAF - minor allele frequency.

## Competing interests

Y.S.A. is the founder and co-owner of PolyOmica, a private research organization that specializes in computational and statistical (gen)omics.

## Funding

The KORA study was initiated and financed by the Helmholtz Center Munich—German Research Center for Environmental Health, which is funded by the German Federal Ministry of Education and Research and by the State of Bavaria. The KORA study was supported by the Munich Center of Health Sciences (MC-Health), Ludwig Maximilian University of Munich, as part of the LMUinnovativ project.

This work was supported by the European Union FP7 framework project Pain-Omics (grant 602736).

S.Z.S. was supported by the Russian Ministry of Science and Education under the 5–100 Excellence Programme. Y.S.A.was supported by the Federal Agency of Scientific Organisations via the Institute of Cytology and Genetics (project 0324-2018-0017). Y.A.T. was supported by the Federal Agency of Scientific Organisations via the Institute of Cytology and Genetics (project 0324-2018-0017) and by the Russian Foundation for Basic Research (project 16-04-00360).

## Author contributions

Y.A.T., C.G., and Y.S.A. designed and supervised the study; C.P., J.A., K.G., and R.W.-S. collected the data; C.G. and K.S. contributed data for the analysis; Y.A.T., O.O.Z., and S.Z.S. analyzed the data; Y.A.T., Y.S.A., C.G., O.O.Z., J.K., and K.S. discussed and interpreted the results; Y.A.T., O.O.Z., C.G., and Y.S.A. wrote the manuscript. All authors contributed to and approve the final version of the manuscript.

## Supplementary Material

giga-d-17-00337_original_submission.pdf

giga-d-17-00337_revision_1.pdf

giga-d-17-00337_revision_2.pdf

giga-d-17-00337_revision_3.pdf

response_to_reviewer_comments_original_submission.pdf

response_to_reviewer_comments_revision_1.pdf

response_to_reviewer_comments_revision_2.pdf

reviewer_1_report_(original_submission) -- Simina Boca 1/22/2018 Reviewed

reviewer_1_report_revision_1 -- Simina Boca 5/25/2018 Reviewed

reviewer_2_report_(original_submission) -- Tim Ebbels 2/2/2018 Reviewed

Supplemental Files

## References

[1] Visscher PM, Brown MA, McCarthy MI, Five years of GWAS discovery. Am J Hum Genet. 2012;90:7–24. 22243964 10.1016/j.ajhg.2011.11.029 PMC3257326

[2] Ritchie MD, Holzinger ER, Li R Methods of integrating data to uncover genotype–phenotype interactions. Nat Rev Genet. 2015;16:85–97. 25582081 10.1038/nrg3868

[3] van der Sijde MR, Ng A, Fu J Systems genetics: from GWAS to disease pathways. Biochim Biophys Acta - Mol Basis Dis. 2014;1842:1903–9. 10.1016/j.bbadis.2014.04.025 24798234

[4] Hicks AA, Pramstaller PP, Johansson A, Genetic determinants of circulating sphingolipid concentrations in European populations. PLoS Genet. 2009;5:e1000672. 19798445 10.1371/journal.pgen.1000672 PMC2745562

[5] Suhre K, Shin S-Y, Petersen A-K Human metabolic individuality in biomedical and pharmaceutical research. Nature. 2011;477:54–60. 21886157 10.1038/nature10354 PMC3832838

[6] Inouye M, Ripatti S, Kettunen J Novel loci for metabolic networks and multi-tissue expression studies reveal genes for atherosclerosis. PLoS Genet. 2012;8:e1002907. 22916037 10.1371/journal.pgen.1002907 PMC3420921

[7] Draisma HHM, Pool R, Kobl M, Genome-wide association study identifies novel genetic variants contributing to variation in blood metabolite levels. Nat Commun. England 2015;6:7208. 26068415 10.1038/ncomms8208 PMC4745136

[8] Kettunen J, Demirkan A, Würtz P Genome-wide study for circulating metabolites identifies 62 loci and reveals novel systemic effects of LPA. Nat Commun. 2016;7:11122. 27005778 10.1038/ncomms11122 PMC4814583

[9] Cichonska A, Rousu J, Marttinen P metaCCA: summary statistics-based multivariate meta-analysis of genome-wide association studies using canonical correlation analysis. Bioinformatics. 2016;32:1981–9. 27153689 10.1093/bioinformatics/btw052 PMC4920109

[10] Stephens M A unified framework for association analysis with multiple related phenotypes. PLoS One. 2013;8:e65245. 23861737 10.1371/journal.pone.0065245 PMC3702528

[11] O'Reilly PF, Hoggart CJ, Pomyen Y, MultiPhen: joint model of multiple phenotypes can increase discovery in GWAS. PLoS One. 2012;7:e34861. 22567092 10.1371/journal.pone.0034861 PMC3342314

[12] Galesloot TE, van Steen K, Kiemeney LALM, A comparison of multivariate genome-wide association methods. PLoS One. 2014;9:e95923. 24763738 10.1371/journal.pone.0095923 PMC3999149

[13] Shen X, Klarić L, Sharapov S Multivariate discovery and replication of five novel loci associated with immunoglobulin G N-glycosylation. Nat Commun. 2017;8:447. 28878392 10.1038/s41467-017-00453-3 PMC5587582

[14] Schaid DJ, Tong X, Larrabee B Statistical methods for testing genetic pleiotropy. Genetics. 2016;204:483–97. 27527515 10.1534/genetics.116.189308 PMC5068841

[15] Deng Y, Pan W Conditional analysis of multiple quantitative traits based on marginal GWAS summary statistics. Genet Epidemiol. 2017;41:427–36. 28464407 10.1002/gepi.22046 PMC5536980

[16] Cox DR, Hinkley DV Theoretical Statistics. London: Chapman Hall; 1974;1:511.

[17] Smith GD, Ebrahim S “Mendelian randomization”: can genetic epidemiology contribute to understanding environmental determinants of disease?. Int J Epidemiol. 2003;32:1–22. 12689998 10.1093/ije/dyg070

[18] Krumsiek J, Suhre K, Illig T, Gaussian graphical modeling reconstructs pathway reactions from high-throughput metabolomics data. BMC Syst Biol. BioMed Central Ltd; 2011;5:21. 21281499 10.1186/1752-0509-5-21 PMC3224437

[19] Tsepilov YA, Shin S-Y, Soranzo N Nonadditive effects of genes in human metabolomics. Genetics. 2015;200:707–18. 25977471 10.1534/genetics.115.175760 PMC4512538

[20] Xie W, Wood AR, Lyssenko V Genetic variants associated with glycine metabolism and their role in insulin sensitivity and type 2 diabetes. Diabetes. 2013;62:2141–50. 23378610 10.2337/db12-0876 PMC3661655

[21] Shin S-Y, Fauman EB, Petersen A-K, An atlas of genetic influences on human blood metabolites. Nat Genet. 2014;46:543–50. 24816252 10.1038/ng.2982 PMC4064254

[22] Falconer DS, Mackay TFC Introduction to Quantitative Genetics. 4th ed. Pearson; 1996 ISBN:978-0582243026

[23] Cheverud JM A comparison of genetic and phenotypic correlations. Evolution. 1988;42:958–68. 28581166 10.1111/j.1558-5646.1988.tb02514.x

[24] Roff DA The estimation of genetic correlations from phenotypic correlations: a test of Cheverud's conjecture. Heredity (Edinb). 1995;74:481–90.

[25] Lynch M, Walsh B, et al Genetics and Analysis of Quantitative Traits. Sunderland, MA: Sinauer; 1998.

[26] Bulik-Sullivan B, Finucane HK, Anttila V An atlas of genetic correlations across human diseases and traits. Nat Genet. Nature Publishing Group; 2015;47:1236–41. 26414676 10.1038/ng.3406 PMC4797329

[27] Zhu Z, Zhang F, Hu H, Integration of summary data from GWAS and eQTL studies predicts complex trait gene targets. Nat Genet. 2016;48:481–7. 27019110 10.1038/ng.3538

[28] Pickrell JK, Berisa T, Liu JZ Detection and interpretation of shared genetic influences on 42 human traits. Nat Genet. 2016, 78;709–717. 10.1038/ng.3570 PMC5207801 27182965

[29] Giambartolomei C, Vukcevic D, Schadt EE, Bayesian test for colocalisation between pairs of genetic association studies using summary statistics. PLoS Genet. 2014;10:e1004383. 24830394 10.1371/journal.pgen.1004383 PMC4022491

[30] Aschard H, Guillemot V, Vilhjalmsson B Covariate selection for association screening in multiphenotype genetic studies. Nat Genet. 2017;49:1789–95. 29038595 10.1038/ng.3975 PMC5797835

[31] Wichmann H-E, Gieger C, Illig T KORA-gen–resource for population genetics, controls and a broad spectrum of disease phenotypes. Gesundheitswesen. 2005;67(Suppl 1):S26–30. 16032514 10.1055/s-2005-858226

[32] Illig T, Gieger C, Zhai G A genome-wide perspective of genetic variation in human metabolism. Nat Genet. 2010;42:137–41. 20037589 10.1038/ng.507 PMC3773904

[33] Kolz M, Johnson T, Sanna S Meta-analysis of 28 141 individuals identifies common variants within five new loci that influence uric acid concentrations. PLoS Genet. 2009;5:e1000504. 19503597 10.1371/journal.pgen.1000504 PMC2683940

[34] Kim S ppcor: an R package for a fast calculation to semi-partial correlation coefficients. Commun Stat Appl Methods. 2015;22:665–74. 26688802 10.5351/CSAM.2015.22.6.665 PMC4681537

[35] Marchetti GM Independencies induced from a graphical Markov model after marginalization and conditioning: the R Package ggm. J Stat Softw. 2006;15, 1–15.. http://www.jstatsoft.org/v15/i06/.

[36] Fabregat-Traver D, Sharapov SZ, Hayward C High-performance mixed models based genome-wide association analysis with omicABEL software. F1000Research. 2014;3:200. 25717363 10.12688/f1000research.4867.1 PMC4329600

[37] Beasley TM, Erickson S, Allison DB Rank-based inverse normal transformations are increasingly used, but are they merited?. Behav Genet. 2009;39:580–95. 19526352 10.1007/s10519-009-9281-0 PMC2921808

[38] Devlin B, Roeder K Genomic control for association studies. Biometrics. 1999;55:997–1004. 11315092 10.1111/j.0006-341x.1999.00997.x

[39] Tsepilov Y, Sharapov S, Aulchenko Y A network-based conditional genetic association analysis of the human metabolome [Source Code], CodeOcean, 2018; https://doi.org/10.24433/CO.3b5ea77b-859a-4db9-af44-b8b6aeb88664.v2. 10.1093/gigascience/giy137 PMC6287100 30496450

[40] Pers TH, Karjalainen JM, Chan Y Biological interpretation of genome-wide association studies using predicted gene functions. Nat Commun. 2015;6:5890. 25597830 10.1038/ncomms6890 PMC4420238

[41] Staley JR, Blackshaw J, Kamat MA, PhenoScanner: a database of human genotype–phenotype associations. Bioinformatics. 2016;32:3207–9. 27318201 10.1093/bioinformatics/btw373 PMC5048068

[42] Tsepilov YA, Sharapov SZ, Zaytseva OO Supporting data for “A network-based conditional genetic association analysis of the human metabolome.”. GigaScience Database. 2018 http://dx.doi.org/10.5524/100507. 10.1093/gigascience/giy137 PMC6287100 30496450

