# Correction to: A network-based conditional genetic association analysis of the human metabolome

**DOI:** 10.1093/gigascience/giz162

**Published:** 2019-12-30

**Authors:** Y A Tsepilov, S Z Sharapov, O O Zaytseva, J Krumsiek, C Prehn, J Adamski, G Kastenmuller, R Wang-Sattler, K Strauch, C Gieger, Y S Aulchenko

**Affiliations:** 1 Institute of Cytology and Genetics SB RAS, Novosibirsk, Lavrentieva Ave. 10, 630090, Russia; 2 Natural Scince Department, Novosibirsk State University, Novosibirsk, Pirogova Str. 1, 630090, Russia; 3 Institute of Computational Biology, Helmholtz Center Munich - German Research Center for Environmental Health, Neuherberg, Ingolstadter Landtrasse 1, 85764, Germany; 4 Institute of Experimental Genetics, Genome Analysis Center, Helmholtz Center Munich - German Research Center for Environmental Health, Neuherberg, Ingolstadter Landtrasse 1, 85764, Germany; 5 Institute of Experimental Genetics, Life and Food Science Center Weihenstephan, Technical University of Munich, Freising-Weihenstephan, Arcisstrasse 21, 80333, Germany; 6 German Center for Diabetes Research, Helmholtz Center Munich - German Research Center for Environmental Health, Neuherberg, Ingolstadter Landtrasse 1, 85764, Germany; 7 Institute of Bioinformatics and Systems Biology, Helmholtz Center Munich - German Research Center for Environmental Health, Neuherberg, Ingolstadter Landtrasse 1, 85764, Germany; 8 Research Unit of Molecular Epidemiology, Helmholtz Center Munich - German Research Center for Environmental Health, Neuherberg, Ingolstadter Landtrasse 1, 85764, Germany; 9 Institute of Epidemiology II, Helmholtz Center Munich - German Research Center for Environmental Health, Neuherberg, Ingolstadter Landtrasse 1, 85764, Germany; 10 Institute of Genetic Epidemiology, Helmholtz Center Munich - German Research Center for Environmental Health, Neuherberg, Ingolstadter Landtrasse 1, 85764, Germany; 11 Chair of Genetic Epidemiology, IBE, Faculty of Medicine, LMU Munich, Munich, Butenandstrasse 5, 81377, Germany; 12 PolyOmica,’s-Hertogenbosch, Het Vlaggeschip 61, 5237 PA, The Netherlands

In the original version of the article “A network-based conditional genetic association analysis of the human metabolome” by Y.A. Tsepilov et al. [1], there was a typographical error in the surname of the fourth author (Jan Krumsek) and a typo in Acknowledgments in the surname of Athina Spilopoulou. These typos have been corrected, and the authors apologize for the mistakes.
