# Supplementary material for: A network-based conditional genetic association analysis of the human metabolome
Source: Gigascience. 2018 Nov 29;7(12):giy137. doi: 10.1093/gigascience/giy137 (PMC6287100; doi:10.1093/gigascience/giy137)
Supplement: giga-d-17-00337_revision_3.pdf [file giy137_giga-d-17-00337_revision_3.pdf]

GigaScience

A network-based conditional genetic association analysis of the human metabolome

--Manuscript Draft--

**Manuscript Number:**	GIGA-D-17-00337R3
**Full Title:**	A network-based conditional genetic association analysis of the human metabolome
**Article Type:**	Technical Note
**Funding Information:**	the European Union FP7 framework project Pain-Omics (602736) — Dr. Christian Gieger<br>Ministry of Education and Science of the Russian Federation (the 5-100 Excellence Programme) — Mr. Sodbo Zh. Sharapov<br>the Federal Agency of Scientific Organisations via the Institute of Cytology and Genetics (0324-2018-0017) — Dr. Yakov A. Tsepilov<br>the Federal Agency of Scientific Organisations via the Institute of Cytology and Genetics (0324-2018-0017) — Prof. Yurii S. Aulchenko
**Abstract:**	Background: Genome-wide association studies have identified hundreds of loci that influence a wide variety of complex human traits; however, little is known regarding the biological mechanism of action of these loci. The recent accumulation of functional genomics (“omics”), including metabolomics data, has created new opportunities for studying the functional role of specific changes in the genome. Functional genomic data are characterized by their high dimensionality, the presence of (strong) statistical dependency between traits, and—potentially—complex genetic control. Therefore, the analysis of such data requires specific statistical genetics methods.<br>Results: To facilitate our understanding of the genetic control of omics phenotypes, we propose a trait-centered, network-based conditional genetic association (cGAS) approach for identifying the direct effects of genetic variants on omics-based traits. For each trait of interest, we selected from a biological network a set of other traits to be used as covariates in the cGAS. The network can be reconstructed either from biological pathway databases (a mechanistic approach) or directly from the data, using a Gaussian Graphical Model applied to the metabolome (a data-driven approach). We derived mathematical expressions which allow comparison of the power of univariate analyses with conditional genetic association analyses. We then tested our approach using data from a population-based KORA study (n=1784 subjects, 1.7 million SNPs) with measured data for 151 metabolites.<br>Conclusions: We found that compared to single-trait analysis, performing a genetic association analysis that includes biologically relevant covariates can either gain or lose power, depending on specific pleiotropic scenarios, for which we provide empirical examples. In the context of analyzed metabolomics data, the mechanistic network approach had more power compared to the data-driven approach. Nevertheless, we believe that our analysis shows that neither a prior-knowledge-only approach nor a phenotypic-data-only approach is optimal, and we discuss possibilities for improvement.
**Corresponding Author:**	Yurii Aulchenko<br>Institute of Cytology and Genetics SB RAS<br>Novosibirsk, RUSSIAN FEDERATION
**Corresponding Author Secondary Information:**	
**Corresponding Author's Institution:**	Institute of Cytology and Genetics SB RAS
**Corresponding Author's Secondary Institution:**	
**First Author:**	Yakov A. Tsepilov, Ph.D.
**First Author Secondary Information:**	

Powered by Editorial Manager® and ProduXion Manager® from Aries Systems Corporation

**Order of Authors:**	Yakov A. Tsepilov, Ph.D.
	Sodbo Zh. Sharapov
	Olga O. Zaytseva, Ph.D.
	Jan Krumsek, Ph.D.
	Cornelia Prehn, Ph.D.
	Jerzy Adamski, Ph.D.
	Gabi Kastenmüller, Ph.D.
	Rui Wang-Sattler, Ph.D.
	Konstantin Strauch, Ph.D.
	Christian Gieger, Ph.D.
	Yurii S. Aulchenko, Ph.D.
**Order of Authors Secondary Information:**	
**Response to Reviewers:**	Dear Hans,<br><br>Please find attached our revised manuscript (in .docx format). We have added citations to “Availability of Data and Materials” section and to reference list. We also have removed highlighting in red and changed affiliation #11.<br><br>Yours Sincerely. also on behalf of other authors,<br>prof. Yurii Aulchenko and dr. Yakov Tsepilov
**Additional Information:**	
**Question**	**Response**
Are you submitting this manuscript to a special series or article collection?	No
**Experimental design and statistics**<br><br>Full details of the experimental design and statistical methods used should be given in the Methods section, as detailed in our Minimum Standards Reporting Checklist. Information essential to interpreting the data presented should be made available in the figure legends.<br><br>Have you included all the information requested in your manuscript?	Yes
**Resources**<br><br>A description of all resources used, including antibodies, cell lines, animals and software tools, with enough information to allow them to be uniquely	Yes

identified, should be included in the Methods section. Authors are strongly encouraged to cite Research Resource Identifiers (RRIDs) for antibodies, model organisms and tools, where possible.<br><br>Have you included the information requested as detailed in our Minimum Standards Reporting Checklist?	
**Availability of data and materials**<br><br>All datasets and code on which the conclusions of the paper rely must be either included in your submission or deposited in publicly available repositories (where available and ethically appropriate), referencing such data using a unique identifier in the references and in the "Availability of Data and Materials" section of your manuscript.<br><br>Have you have met the above requirement as detailed in our Minimum Standards Reporting Checklist?	Yes

Manuscript
Click here to view linked References
Click here to access/download;Manuscript;20181105_cGWAS_manuscript_

A network-based conditional genetic association analysis of the human metabolome

Y.A. Tsepilov[1,2], S.Z. Sharapov[2], O.O. Zaytseva[1,2], J. Krumsek[3], C. Prehn[4], J. Adamski[4,5,6], G. Kastenmüller[7], R. Wang-Sattler[6,8,9], K. Strauch[10,11], C. Gieger[6,8,9], Y.S. Aulchenko[1,2,12*]

1 Institute of Cytology and Genetics SB RAS, Novosibirsk, Russia
2 Novosibirsk State University, Novosibirsk, Russia
3 Institute of Computational Biology, Helmholtz Center Munich - German Research Center for Environmental Health, Neuherberg, Germany
4 Institute of Experimental Genetics, Genome Analysis Center, Helmholtz Center Munich - German Research Center for Environmental Health, Neuherberg, Germany
5 Institute of Experimental Genetics, Life and Food Science Center Weihenstephan, Technical University of Munich, Freising-Weihenstephan, Germany
6 German Center for Diabetes Research, Neuherberg, Germany
7 Institute of Bioinformatics and Systems Biology, Helmholtz Center Munich - German Research Center for Environmental Health, Neuherberg, Germany
8 Research Unit of Molecular Epidemiology, Helmholtz Center Munich - German Research Center for Environmental Health, Neuherberg, Germany
9 Institute of Epidemiology II, Helmholtz Center Munich - German Research Center for Environmental Health, Neuherberg, Germany
10 Institute of Genetic Epidemiology, Helmholtz Center Munich - German Research Center for Environmental Health, Neuherberg, Germany
11 Chair of Genetic Epidemiology, IBE, Faculty of Medicine, LMU Munich, 81377 Munich, Germany
12 PolyOmica, 's-Hertogenbosch, The Netherlands

* Correspondence to

Yurii S. Aulchenko

Institute of Cytology and Genetics SB RAS, 630090 Novosibirsk, Russia

yurii@bionet.nsc.ru

Keywords: genome-wide association study; multivariate model; metabolomics; conditional analysis; pleiotropy

Abstract

Background: Genome-wide association studies have identified hundreds of loci that influence a wide variety of complex human traits; however, little is known regarding the biological mechanism of action of these loci. The recent accumulation of functional genomics ("omics"), including metabolomics data, has created new opportunities for studying the functional role of specific changes in the genome. Functional genomic data are characterized by their high dimensionality, the presence of (strong) statistical dependency between traits, and—potentially—complex genetic control. Therefore, the analysis of such data requires specific statistical genetics methods.

Results: To facilitate our understanding of the genetic control of omics phenotypes, we propose a trait-centered, network-based conditional genetic association (cGAS) approach for identifying the direct effects of genetic variants on omics-based traits. For each trait of interest, we selected from a biological network a set of other traits to be used as covariates in the cGAS. The network can be reconstructed either from biological pathway databases (a mechanistic approach) or directly from the data, using a Gaussian Graphical Model applied to the metabolome (a data-driven approach). We derived mathematical expressions which allow comparison of the power of univariate analyses with conditional genetic association analyses. We then tested our approach using data from a population-based KORA study (n=1784 subjects, 1.7 million SNPs) with measured data for 151 metabolites.

Conclusions: We found that compared to single-trait analysis, performing a genetic association analysis that includes biologically relevant covariates can either gain or lose power, depending on specific pleiotropic scenarios, for which we provide empirical examples. In the context of analyzed metabolomics data, the mechanistic network approach had more power compared to the data-driven approach. Nevertheless, we believe that our analysis shows that neither a prior-knowledge-only approach nor a phenotypic-data-only approach is optimal, and we discuss possibilities for improvement.

Short abstract

We propose a trait-centric network-based conditional approach for performing a genetic association analysis of multivariate omics phenotypes. This approach can incorporate existing biological knowledge regarding biological pathways obtained from external sources and is designed to specifically test for direct genetic effects. We applied this approach to existing metabolomics data and found that it may have more power by having increased accuracy of genetic effect estimates in the presence of specific “counterintuitive” pleiotropic scenarios in which locus-specific genetically induced and residual covariance are opposite, but it may lose power when genetically induced and residual covariance have a concordant sign. We provide empirical examples of different pleiotropic scenarios that we observed in metabolomics, and we discuss possible additional applications for this approach.

Background

Genome-wide association studies (GWAS) are a highly popular method for identifying alleles that affect complex traits in humans, including the risk of common diseases. In the past decade, GWASs have enabled the identification of thousands of loci, significantly increasing our understanding of the genetic basis underlying the control of complex human traits [1]. On the other hand, this has had only a limited impact on the development of biomarkers and therapeutic agents; in most cases, any association found using GWAS approach can only serve as a starting point for future research, rather than providing a direct answer to the question of the genetic region's precise biological function. The recent accumulation of functional genomics (or "omics" for short) data—including information regarding the levels of gene expression (the transcriptome), metabolites (the metabolome), proteins (the proteome), and glycosylation (the glycome)—can provide new insight into the functional role of specific changes in the genome [2,3].

Metabolomics is an emerging field that has been studied extensively in the past decade. A number of GWASs of metabolites have been performed using various platforms [4–8], revealing literally dozens of loci associated with variations in various lipid species, amino acids, and other small molecules. Linking the variants that underlie these variations in metabolomics with various diseases can provide functional insight into the many disease-related associations that were reported in previous studies, including cardiovascular and kidney disease, type 2 diabetes, cancer, gout, venous thromboembolism, and Crohn's disease [5].

However, analyzing metabolomics data requires specialized statistical methods due to their characteristically high dimensionality and the presence of statistical dependencies that reflect biological relationships between different variables. Conventional univariate GWAS (uGAS) approaches ignore any possible dependencies between different omics traits, which can confound the biological interpretation of the results and may lead to a loss of statistical power. On the other hand, utilizing multivariate phenotype information increases the statistical power of the association tests compared to univariate analysis [9–12]. Despite a large number of methodological studies, however, only a few empirical multivariate GWASs have been published using data for humans. We recently demonstrated [13] that using a multivariate analysis can substantially increase the power of locus identification in the context of human *N*-glycomics; indeed, not only did our multivariate analysis double the number of loci identified in the analysis sample, but also all five novel loci were strongly replicated. With respect to metabolomics, Inouye et al. [6] performed a multivariate GWAS on 130 metabolites (grouped in 11 sets) measured in approximately 6600 individuals. They found that multivariate analysis doubled the number of loci detected in this sample; seven of these additional loci discovered were novel loci that had not been identified

previously in other GWAS analyses of related traits. While no replication of novel loci was performed by Inouye et al., we compared the authors' results with a recently published univariate GWAS of metabolomics derived from a cohort containing nearly 25,000 individuals [8]. We found that three of the seven SNPs reported by Inouye et al. have a p-value < 5×10^{-11} for at least one metabolite (i.e., are significant at the genome-wide level after Bonferroni correction for 130 analyses). These findings provide empirical evidence supporting the value of using multivariate methods to analyze the genomics of metabolic traits, at least in the context of locus discovery.

It should be noted that these multivariate methods and tests were developed by statistical geneticists to specifically increase the power of gene identification. In such "gene-centric" tests, the model that includes the effects of genotype on multiple traits is contrasted with the null model in which the gene has no effect on any trait analyzed. Although useful and powerful for genetic mapping, this approach may have limited interpretability in a context in which one is interested in the genetic control and biology of specific trait or a subset of traits (the "trait-centered" view). Several statistical methods have been suggested to address the question of which specific traits are affected in an analyzed ensemble (see for example [10,14]). One such method is based on conditional analysis [15], in which a "target trait" is analyzed as a genotype-dependent variable and related traits are included in the regression model as covariates. Such a modeling approach allows—at least in theory—one to rule out indirect genetic effects (e.g., effects that are in fact solely mediated through some other trait) and study only the genetic effects that directly affect the trait of interest.

Here, we present a statistical model in which a given trait depends on a genetic polymorphism and in which a number of related traits are included in the model as covariates. In this model, the relationship between the genotype and the trait of interest is our primary focus. Analyzing such a model allows us to identify the direct effect of genetics on the trait of interest. Mathematically, the model is equivalent to the model used by Deng and Pan [15]. We first compare this conditional genetic association (cGAS) approach with the standard model in which a trait of interest depends solely on genotype, without other traits used as covariates (i.e., the univariate genetic association—or uGAS—model). We do so by mathematically deriving expressions that allow us to examine the relative power of the uGAS and cGAS approaches, and we identify the situations in which these models are expected to yield different results.

As might be expected—and as demonstrated here—the choice of covariates plays a critical role in conditional analyses. First, we used the assumption that the covariates (i.e., biologically relevant traits) are known. Second, we explored the problem of selecting appropriate covariates, and we tested the approaches by performing a proof-of-principle study using metabolomics data consisting of 151 metabolites (Biocrates assay) obtained from the KORA F4 study (n=1785

individuals). Specifically, we selected covariates based on existing knowledge from metabolite biochemical networks (BN-cGAS) and using a data-driven approach based on Gaussian Graphical Modeling (GGM-cGAS). Finally, we compare and discuss the obtained results, and we discuss possible applications for this analysis based on biologically and/or statistically relevant traits.

Results

The power of performing a conditional analysis of genetic associations

We start with the theoretical substantiation and identification of specific scenarios in which adjusting for biologically relevant covariates can modify the power of an association analysis.

Let us consider a trait of interest, y, covariate c, and genotype g. We can formulate this problem in terms of a linear regression as follows: $y = \mu + \beta_g * g + \beta_c * c + e$, where β_g and β_c are the effects of the genotype and covariate, respectively, and e is the residual noise. Without a loss of generality, we assume that all random variables in this equation are distributed with a mean of zero and a standard deviation of 1, making (partial) regression coefficients equal to (partial) correlation coefficients. Given these assumptions made, the joint distribution of y, g, and c can be specified using a set of three correlation coefficients, ρ_{yg} (the correlation between the trait and the genotype), ρ_{cg} (the correlation between the covariate and the genotype), and ρ_{yc} (the correlation between the trait and the covariate). To test the association between y and g, we use the Wald test, which is defined as the square of the ratio between the effect estimate and its standard error, with the latter estimated under the alternative hypothesis (see [16]). The value of the "univariate" Wald test statistic is calculated as $T_u^2 = \frac{n\,\hat{\rho}_{yg}^2}{\hat{\sigma}_u^2}$, where n is the sample size and $\hat{\sigma}_u^2 = 1 - \hat{\rho}_{yg}^2$ is the estimated residual variance of y. For the conditional test, the Wald test is $T_c^2 = \frac{n\,\hat{\beta}_g^2}{\hat{\sigma}_c^2}$, where $\hat{\beta}_g$ is the estimated partial correlation between the trait y and the genotype g (estimated from the conditional model) and $\hat{\sigma}_c^2$ is the estimated residual variance of y. Note that under the null hypothesis, when n is large, both T_u^2 and T_c^2 are well approximated by chi-square distribution with one degree of freedom. For genetic association studies, n is thousands or orders or magnitude more.

For the conditional model, $\hat{\beta}_g = \hat{\rho}_{yg} - \hat{\beta}_c\ \hat{\rho}_{cg}$; thus, we can rewrite $T_c^2 = n(\hat{\rho}_{yg} - \hat{\beta}_c\ \hat{\rho}_{cg})^2/\hat{\sigma}_c^2$. Consequently, the log-ratio of the conditional and univariate test statistics can be partitioned into two components:

$$\log\left(\frac{T_c^2}{T_u^2}\right) = \log\left(\frac{\hat{\sigma}_u^2}{\hat{\sigma}_c^2}\right) + \log\left(\left[1 - \frac{\hat{\beta}_c\ \hat{\rho}_{cg}}{\hat{\rho}_{yg}}\right]^2\right) \qquad (1)$$

Because the first term in Eq. (1) is dependent only upon residual variances of the two models, we call this term the "noise" component. The second term depends upon the correlations between traits and between the traits and the genotype; we call this term the "pleiotropic" component. Because the noise component ($\hat{\sigma}_u^2/\hat{\sigma}_c^2$) is always ≥1, any possible decrease in the ratio between univariate and conditional tests is determined by the sign and the magnitude of the term

$\hat{\beta}_c\ \hat{\rho}_{cg}/\hat{\rho}_{yg}$. If this term is negative, there will always be an increase in the power of the conditional analysis.

We can re-write $\hat{\beta}_c\ \hat{\rho}_{cg}/\hat{\rho}_{yg}$ as $\hat{\beta}_c\hat{\rho}^*_{yc}$, where $\hat{\rho}^*_{yc} = \hat{\rho}_{cg}/\hat{\rho}_{yg}$ is the component of the correlation between trait *y* and covariate *c*, which is induced by the variation in the genotype *g*. This quantity takes a central place in a Mendelian randomization analysis, which uses a genetic variation to anchor the causality arrow and consequently infers a causal relation between various traits (see for example [17]). Note that whereas $\hat{\rho}^*_{yc}$ reflects the covariance between the trait and the covariate induced by the effect of the genotype, $\hat{\beta}_c$ is conditional on the genotype and is related to the residual sources of covariance between *y* and *c*.

In general, the genetically induced covariance and the residual covariance are expected to have a concordant sign (see Discussion for details and relevant references). Thus, we conclude somewhat surprisingly that when genotype-induced and environmental correlations are similar in sign (i.e., both are positive or both are negative), the product $\hat{\beta}_c\hat{\rho}^*_{yc}$ is positive and the contribution of the second term in Eq. (1) to the relative power is negative. Note that the contribution of the first term in Eq. (1) is always positive; therefore, even if $\hat{\beta}_c\hat{\rho}^*_{yc}$ is positive, the power of a conditional analysis may still be higher than the power of a univariate analysis. In contrast, an "unexpected" product (in which the signs are different and hence $\hat{\beta}_c\hat{\rho}^*_{yc}$ is negative) contributes positively to the relative power of the conditional model. Note that in such a situation, the power of a conditional analysis will always be higher than the power of a univariate analysis.

We can readily extend Eq. (1) to a situation in which *k* covariates are included in the conditional model. Denoting the estimated coefficients of correlation between *g* and covariate *i* as $\hat{\rho}_{gi}$ and the estimated partial correlation between *y* and covariate *i* as $\hat{\beta}_i$ yields the following equation:

$$\log\left(\frac{T_c^2}{T_u^2}\right) = \log\left(\frac{\hat{\sigma}_u^2}{\hat{\sigma}_c^2}\right) + \log\left(\left[1 - \frac{1}{\hat{\rho}_{yg}}\sum_{i=1}^{k}\hat{\beta}_i\hat{\rho}_{gi}\right]^2\right) \qquad (2)$$

When appropriate covariates are selected, performing cGAS using individual-level data becomes rather trivial and can be achieved using standard statistical and software tools in which one estimates the effects of a SNP and covariates. However, cGAS becomes somewhat less trivial if one chooses to use summary-level univariate GWAS data such as data available from previously published studies. The formalization of cGAS in terms of summary univariate GWAS statistics is described in **Supplementary Note 1.** Here, we used methods based on analyzing summary-level data.

Network-based selection of covariates

The ability to select appropriate covariates is extremely important, as it can have direct implications regarding the outcome of the analysis. If the biological/biochemical relationships between traits of interest are known and are summarized in a database(s), this knowledge can be used directly, for example by using all direct neighbors as covariates. We refer to this approach as a biochemical-network driven cGAS (BN-cGAS). Alternatively, the network can be reconstructed in a hypothesis-free, empirical manner from the data, for example using a Gaussian Graphical Model (GGM) [18]. We refer to this approach as a GGM-cGAS.

We compared cGAS and uGAS by performing a genome-wide analysis of genetic effects using summary-level data obtained from the KORA F4 study. This study included 151 metabolites measured in 1784 individuals using the Biocrates assay and imputed at 1,717,498 SNPs.

First, we examined the potential of using cGAS when the covariates are selected based on a known biochemical network (i.e., BN-cGAS). Thus, our analysis was restricted to a subset of 105 metabolites for which at least the one-reaction-step immediate biochemical neighbors are known [18]. This biochemical network incorporates only lipid metabolites, and the pathway reactions cover two groups of pathways: (1) fatty acid biosynthesis reactions, which apply to the metabolite classes lyso-PC, diacyl-PC, acyl-alkyl-PC, and sphingomyelins; and (2) β-oxidation reactions that reflect fatty acid degradation and apply to acylcarnitines. The β-oxidation model consists of a linear chain of C2 degradation steps (C10 to C8 to C6, etc.). The number of covariates ranged from 1 to 4, with mean and median values of 2.48 covariates and 2 covariates, respectively.

Table 1 lists the 11 loci that were significant in either BN-cGAS or uGAS and fell into known associated regions (see **Supplementary Note 2**). Of these 11 loci, ten and nine loci could be identified by BN-cGAS and uGAS, respectively. Compared to uGAS, BN-cGAS identified one fewer locus (*ETFDH*), but identified two more (*ACSL1* for PC ae C42:5 and *PKD2L1* for lyso-PC a C16:1). It is interesting to note that for *ACSL1*, the effect of SNP rs4862429 on PC ae C42:5 was highly significant (p=7e-11) with BN-cGAS, but was not significant (p=0.7) with uGAS; this outcome is to be expected under the model of unexpected pleiotropy.

Next, to test whether using BN-cGAS increases the average power of the association analysis, we compared the BN-cGAS and uGAS chi-square test results for the loci listed in **Table 1**. Within a given locus, we compared the maximum test value. The average ratio of the maximum test statistic between BN-cGAS and uGAS was 1.47, indicating that on average, BN-cGAS led to higher test statistic values. However, when we used a paired-sample Wilcoxon test to compare the best chi-square test results between BN-cGAS and uGAS, the difference between the two methods was not significant (p=0.123) (see Supplementary Table S1A).

For the SNPs listed in **Table 1**, we then used Eq. (2) to partition the log-ratio of the BN-cGAS and uGAS statistics values into "noise" and "pleiotropic" components. As shown in **Figure 1**, the regression slope of the second (i.e., "pleiotropic") component is considerably higher than the slope of the noise component; in other words, the ratio is determined primarily by the pleiotropic term in Eq. (2). Moreover, with the exception of the *SLC22A4* locus, the SNP-trait pairs for which BN-cGAS had increased power are the pairs in which the second term in Eq. (2) is either positive or close to zero. In contrast, in the SNP-trait pairs that were not identified using BN-cGAS, the "pleiotropic" term in Eq. (2) had a strong negative contribution.

Next, we investigated the variance-covariance structure of the loci with positive and negative pleiotropic terms. We therefore selected a locus in which the pleiotropic component's contribution to power was positive (rs174547 at *FADS1*) and a locus in which the pleiotropic component's contribution to power was negative (rs8396 at *ETFDH*). **Figure 2** shows the corresponding correlations between the SNP, the trait, and the covariates involved, together with the partial coefficients for the conditional regression of the trait on the SNP and the covariates. With respect to *FADS1* (**Figure 2A**), the correlations between the SNP and the trait (lyso-PC a C20:4) and between the SNP and the covariate (lyso-PC a C20:3) are in opposite directions, generating negative genetically induced covariance between lyso-PC a C20:4 and lyso-PC a C20:3. In contrast, the residual correlation between the trait and the covariate is positive. Therefore, the value of the partial regression coefficient between the SNP and lyso-PC a C20:4, conditional on lyso-PC a C20:3, is greater than that of the coefficient of regression without covariates.

With respect to the second example, *ETFDH* (**Figure 2B**), we found that the conditional regression of C10 on rs8396 and two covariates (C8 and C12, two medium-chain acylcarnitines) led to a smaller SNP partial regression coefficient compared to an unconditional regression; this is because all of the terms in $\sum_{i=1}^{k} \hat{\beta}_i \hat{\rho}_{gi} / \hat{\rho}_{yg}$ are positive.

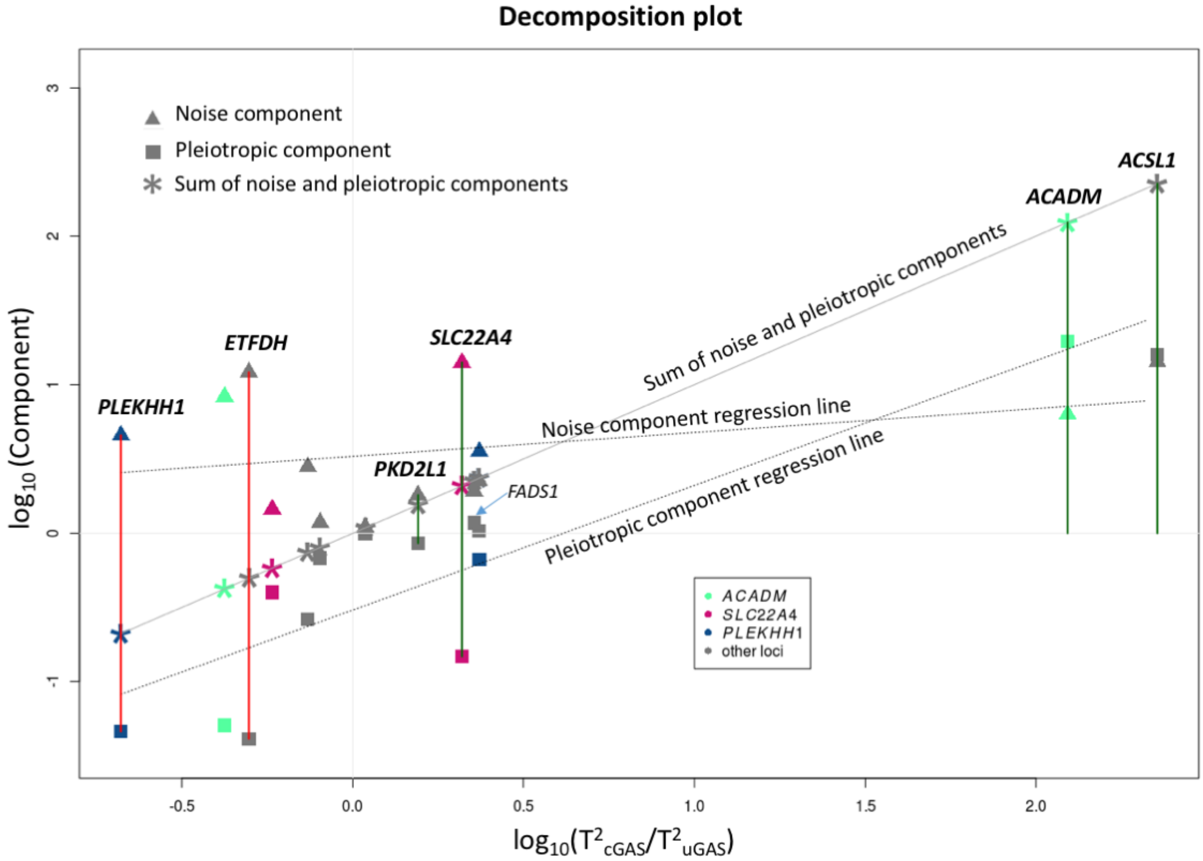

Figure 1. Decomposition of the log-T^2 ratio for cGAS and uGAS into pleiotropic and noise components. Vertically grouped trios (each composed of a square, triangle, and asterisk) correspond to one of fourteen associations in Table 1. The position of a trio on the *x*-axis corresponds to the log-ratio between conditional and univariate test statistic. On the *y*-axis, the asterisk corresponds to the log-ratio of cGAS and uGAS T^2 statistics. The value of the pleiotropic component is depicted by a square, and the value of the noise component is depicted by a triangle. Each trio is shown in gray, except the trios representing the *ACADM*, *SLC22A4*, and *PLEKHH1* loci, for which we have two different associations. The three dotted lines correspond to the regression lines for the two components and their sum. The four dark-green vertical lines indicate the associations that were significant in the cGAS analysis but not in the uGAS analysis, and the two dark-red lines indicates the associations that were significant only in the uGAS analysis.

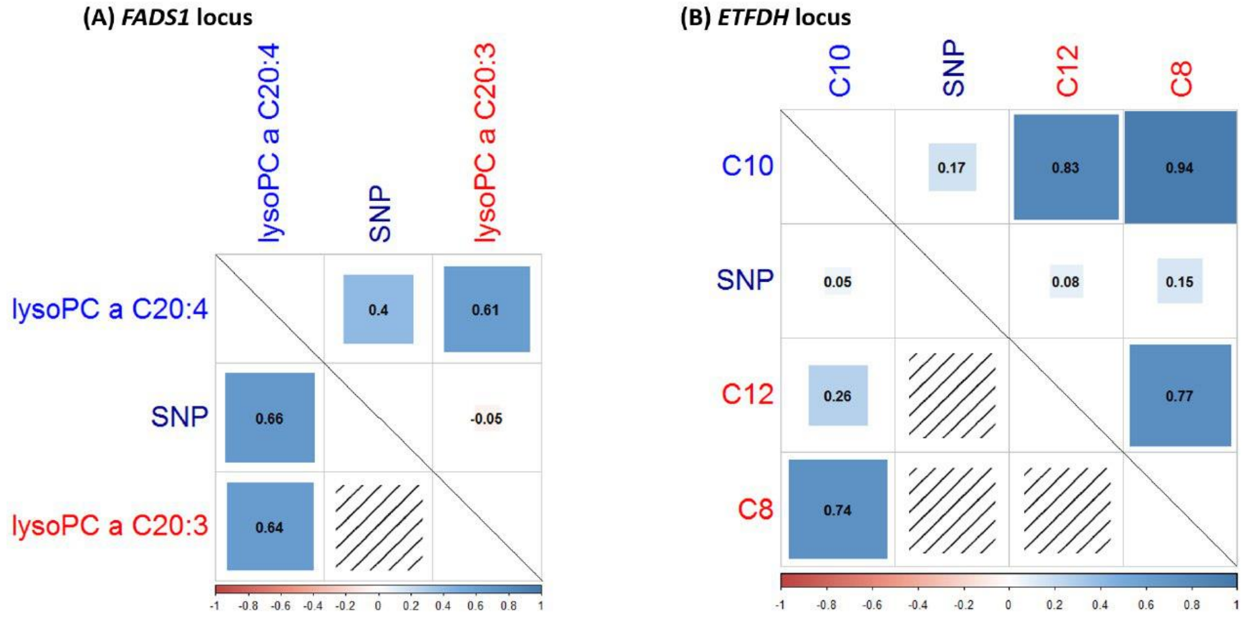

Figure 2. Matrix of correlations (above diagonal line) and the partial regression coefficients of the trait of interest on the SNP genotype and covariate(s) (the first column) for the *FADS1* (A) and *ETFDH* (B) loci. The result of the univariate analysis of regression of the corresponding traits onto SNPs is presented in Supplementary Table S1C. Names of traits used as covariates are in red. The number in a cell indicates the value of correlation (partial regression coefficient). The area of a square is proportional to the absolute value of correlation (partial regression coefficient); the effect magnitude is also reflected by square's color (the scale provided at the bottom of the graph). The *FADS1* locus represents scenario in which the pleiotropic term in Eq. (2) is strongly positive, while for *ETFDH* this term is negative.

Although using a known biochemical network to select covariates has many advantages, it may be somewhat unpractical and perhaps even harmful, as our biochemical knowledge is still relatively incomplete. Therefore, we explored the potential of performing a cGAS in which the covariates are selected using a data-driven approach (GGM-cGAS). The network of metabolites was reconstructed using Gaussian Graphical Models based on partial correlations. For a given metabolite, we selected covariates based on significant partial correlations. Specifically, we used the following threshold as proposed previously [18]: a p-value $\leq$ (0.01/number of calculated partial correlations), which corresponds to a cut-off at $p \leq 8.83 \times 10^{-7}$. The network used in our analysis is shown in **Supplementary Figure S1**.

To compare GGM-cGAS with BN-cGAS, we used the same set of metabolites that we used for BN-cGAS to run our GGM-cGAS analysis; these results are presented in **Supplementary Table S1B**. We found 15 SNP-trait pairs clustered at 10 known loci (see Supplementary Note 2) that were detected by either GGM-cGAS or BN-cGAS. More covariates were included in the GGM-cGAS analysis (ranging from 1 to 18, with mean and median values of 7.6 covariates and 7 covariates, respectively) than in the BN-cGAS analysis. Thus, we predicted that GGM-cGAS would have relatively more power than BN-cGAS due to reduced noise (term 1 in Eq. (2)); on the

other hand, GGM-cGAS might lose power because of reduced occurrence of unexpected pleiotropy (term 2 in Eq. (2)).

For the best SNP-trait pairs detected by GGM-cGAS or BN-cGAS, we computed the components in Eq. (2) and compared these components using a paired-sample Wilcoxon test. We found that the noise component in Eq. (2) was always larger for GGM-cGAS, with a mean difference of 0.29 (p=6 × 10^{-5}). Moreover, the second "pleiotropic" component in Eq. (2) was generally smaller for GGM-cGAS than for BN-cGAS, with a mean difference of -0.47 (p=0.015); nevertheless, for three out of 15 GGM-cGAS SNP-trait pairs, the pleiotropic component was positive. The average chi-square value was 25% smaller for GGM-cGAS than for BN-cGAS, indicating an average loss of power (although this loss was not significant; p=0.5 based on a paired Wilcoxon test).

Next, we investigated further the potential of using cGAS under realistic conditions to a full extent by analyzing all 151 available metabolites using GGM-cGAS and comparing these results with the results of uGAS (**Table 2** and **Supplementary Figure S2)**. In total, uGAS detected 15 loci at the genome-wide significance level $p \leq 5 \times 10^{-8}/151$ (i.e., $p < 3.3 \times 10^{-10}$). On the other hand, GGM-cGAS identified 19 significant loci using the same threshold. As expected, the standard errors of the genetic effect estimates were smaller for GGM-cGAS than for uGAS (**Table 2** and **Supplementary Figure S3**). A total of 14 loci were detected by both uGAS and GGM-cGAS. GGM-cGAS failed to identify one locus that was identified by uGAS (C5:1-DC at rs2943644), but identified five loci that were missed by uGAS. Three of the five loci identified solely by GGM-cGAS affect amino acids, and the remaining two loci affect acylcarnitines. It is important to note that the loci identified by BN-cGAS (when we analyzed 105 metabolites) are a subset of the 19 loci that were identified by GGM-cGAS (when we used all 151 metabolites).

Finally, we searched the available literature for the loci listed in **Table 2** (see **Supplementary Note 2** for details). From the 20 loci that we report here, 15 were found to be significant at the genome-wide level in a recent large (n=7478) meta-analysis of Biocrates metabolomics data reported by Draisma et al. [7]. Some of the metabolites analyzed in our study were not analyzed by Draisma et al. [7]; nevertheless, for 11 out of these 15 loci, we observed a significant association for the same SNP-metabolite pair; for three loci, the strongest association was with a metabolite in the same class, and for one locus the strongest association was with a metabolite from a different lipid class (see **Supplementary Table S2**). For the other five loci that were not significant in the study by Draisma et al. [7], we determined whether these five loci were significant and replicated in a study by Tsepilov et al. [19]. It should be noted that Tsepilov et al. analyzed the ratios of metabolites and also used the KORA F4 data set in their discovery stage, although they used another cohort (TwinsUK) for replication. Of these five loci, two were also

significant in the study by Tsepilov et al. [19]; moreover, for both of these loci the metabolite analyzed in our study was included in the ratios analyzed by Tsepilov et al. One of the five loci was associated with the same trait in two other studies [20,21]. Finally, we found no prior published evidence of any association with metabolites for rs2943644 (*LOC646736*) or rs17112944 (*LOC728755*). Taking into account that this association was not found in (much) bigger meta-analysis, we conclude the observed associations with rs17112944 and rs2943644 as likely false positives, and these two loci were excluded from further consideration.

Table 1. Eleven loci identified by BN-cGAS and uGAS on metabolites for which at least one one-reaction-step neighbor was available.

							uGAS		*cGAS*				
Locus	**SNP**	**Metabolite**	**chr:pos**	**Gene**	**effA/refA**	**EAF**	**beta (se)**	**P-value GC**	**Beta (se)**	***P*-value GC**	**N_{cov}**	**Noise**	**Pleiotropic**
						uGAS & cGAS							
1	rs211718	C8	1:75879263	*ACADM*	T/C	0.3	-0.45(0.034)	6.35E-39	-0.10(0.012)	4.45E-17	1	0.92	-1.29
1	rs211718	C12	1:75879263	*ACADM*	T/C	0.3	-0.04(0.036)	2.21E-01	0.20(0.014)	4.07E-42	3	0.80	1.29
2	rs7705189	PC ae C42:5	5:131651257	*SLC22A4*	G/A	0.47	0.15(0.034)	8.83E-06	0.06(0.009)	9.63E-11	3	1.16	-0.83
2	rs419291	C5	5:131661254	*SLC22A4*	T/C	0.38	0.26(0.035)	6.62E-14	0.17(0.029)	1.40E-08	1	0.16	-0.40
3	rs9368564	PC aa C42:5	6:11168269	*ELOVL2*	G/A	0.25	-0.29(0.039)	4.64E-14	-0.15(0.024)	1.06E-10	3	0.45	-0.58
4	rs12356193	C0	10:61083359	*SLC16A9*	G/A	0.17	-0.51(0.046)	4.93E-28	-0.42(0.042)	8.83E-23	1	0.07	-0.17
5	rs174547	lyso-PC a C20:4	11:61327359	*FADS1*	C/T	0.7	0.61(0.033)	2.12E-75	0.66(0.024)	2.65E-169	1	0.29	0.07
6	rs2066938	C4	12:119644998	*ACADS*	G/A	0.27	0.73(0.033)	1.07E-104	0.72(0.031)	4.26E-116	1	0.05	0.00
7	rs10873201	PC ae C36:5	14:67036352	*PLEKHH1*	T/C	0.45	-0.26(0.034)	6.34E-14	-0.21(0.018)	5.72E-31	2	0.55	-0.18
7	rs1077989	PC ae C32:2	14:67045575	*PLEKHH1*	C/A	0.46	-0.30(0.034)	9.22E-19	-0.06(0.016)	5.39E-05	3	0.66	-1.34
8	rs4814176	PC ae C40:2	20:12907398	*SPTLC3*	T/C	0.36	0.24(0.035)	5.60E-12	0.25(0.023)	1.28E-25	4	0.35	0.02
						Only uGAS							
9	rs8396	C10	4:159850267	*ETFDH*	C/T	0.71	0.26(0.037)	1.32E-12	0.05(0.010)	5.08E-07	2	1.09	-1.39
						Only cGAS							
10	rs4862429	PC ae C42:5	4:186006834	*ACSL1*	T/C	0.31	0.02(0.037)	6.63E-01	-0.06(0.010)	7.01E-11	3	1.15	1.20
11	rs603424	Lyso-PC a C16:1	10:102065469	*PKD2L1*	A/G	0.8	0.23(0.042)	4.83E-08	0.21(0.031)	1.76E-11	1	0.26	-0.07

Notes: The best SNP-metabolite pair is shown for each locus. chr:pos refers to the physical position of the SNP; EAF, effect allele frequency; beta (se), the estimated effect and standard error of the SNP; effA/refA, effect allele/reference allele; *P*-value, the *p*-value of the additive model; Gene, the most likely (according to DEPICT) associated gene in the region; N_{cov}, the number of covariates used in cGAS; Noise/Pleiotropic, the values of noise and pleiotropic components of the log-ratio of cGAS and uGAS T^2 statistics.

Table 2. Twenty loci identified by GGM-cGAS and uGAS.

							uGAS		cGAS				
**LOCUS**	**SNP**	**Metabolite**	**chr:pos**	**Gene**	**effA/refA**	**EAF**	**beta (se)**	**P-value GC**	**beta (se)**	**P-value GC**	**N_{cov}**	**Noise**	**Pleiotropic**
						uGAS & cGAS							
**1**	rs211718	C6 (C4:1-DC)	1:75879263	*ACADM*	T/C	0.30	-0.48(0.034)	3.31E-44	-0.13(0.017)	1.21E-13	7	0.61	-1.16
**1**	rs7552404	C6 (C4:1-DC)	1:75908534	*ACADM*	G/A	0.30	-0.48(0.034)	2.14E-44	-0.12(0.017)	2.34E-13	7	0.61	-1.17
**2**	rs483180	Ser	1:120069028	*PHGDH*	G/C	0.30	-0.24(0.037)	2.26E-11	-0.24(0.028)	1.10E-17	2	0.24	-0.02
**2**	rs477992	Ser	1:120059099	*PHGDH*	A/G	0.70	0.24(0.037)	3.50E-11	0.24(0.028)	2.52E-18	2	0.24	0.00
**3**	rs2286963	C9	2:210768295	*ACADL*	G/T	0.63	-0.49(0.032)	4.76E-52	-0.48(0.027)	7.41E-73	3	0.16/	-0.01
**4**	rs8396	C10	4:159850267	*ETFDH*	C/T	0.71	0.26(0.037)	1.32E-12	0.04(0.010)	1.23E-05	8	1.11	-1.53
**4**	rs8396	C7-DC	4:159850267	*ETFDH*	C/T	0.71	-0.09(0.037)	1.67E-02	-0.13(0.019)	2.93E-11	8	0.56	0.33
**5**	rs419291	C5	5:131661254	*SLC22A4*	T/C	0.38	0.26(0.035)	6.62E-14	0.17(0.026)	2.25E-10	3	0.25	-0.40
**5**	rs270613	C5	5:131668482	*SLC22A4*	A/G	0.61	-0.26(0.035)	7.48E-14	-0.17(0.026)	8.24E-11	3	0.25	-0.38
**6**	rs9393903	PC aa C42:5	6:11150895	*ELOVL2*	A/G	0.75	0.29(0.039)	9.13E-14	0.18(0.020)	1.32E-19	6	0.56	-0.38
**6**	rs9368564	PC aa C42:5	6:11168269	*ELOVL2*	G/A	0.25	-0.29(0.039)	4.64E-14	-0.19(0.021)	3.04E-19	6	0.56	-0.40
**7**	rs816411	Ser	7:56138983	*PHKG1*	C/T	0.51	-0.22(0.034)	1.53E-10	-0.19(0.026)	4.83E-13	2	0.23	-0.12
**7**	rs1894832	Ser	7:56144740	*PHKG1*	C/T	0.51	0.21(0.034)	2.33E-10	0.19(0.026)	1.55E-13	2	0.23	-0.09
**8**	rs12356193	C0	10:61083359	*SLC16A9*	G/A	0.17	-0.51(0.046)	4.93E-28	-0.27(0.034)	1.03E-15	3	0.26	-0.53
**9**	rs174547	lyso-PC a C20:4	11:61327359	*FADS1*	C/T	0.70	0.61(0.033)	2.12E-75	0.07(0.011)	2.08E-10	9	0.98	-1.90
**9**	rs174556	PC ae C44:4	11:61337211	*FADS1*	T/C	0.27	0.09(0.038)	1.61E-02	0.21(0.014)	1.17E-48	3	0.84	0.73
**10**	rs2066938	C4	12:119644998	*ACADS*	G/A	0.27	0.73(0.033)	1.07E-104	0.71(0.024)	6.95E-189	2	0.28	-0.02
**11**	rs12879147	PC aa C28:1	14:63297349	*SYNE2*	A/G	0.85	-0.46(0.050)	1.83E-19	-0.12(0.019)	6.87E-11	14	0.86	-1.14
**11**	rs17101394	SM(OH) C14:1	14:63302139	*SYNE2*	A/G	0.83	-0.32(0.050)	1.02E-10	-0.10(0.011)	9.23E-18	7	1.30	-1.05
**12**	rs1077989	PC ae C36:5	14:67045575	*PLEKHH1*	C/A	0.46	-0.26(0.034)	4.96E-14	-0.08(0.010)	2.56E-15	10	1.05	-1.00
**12**	rs1077989	PC ae C32.2	14:67045575	*PLEKHH1*	C/A	0.46	-0.30(0.034)	9.22E-19	-0.05(0.016)	1.35E-03	6	0.67	-1.55
**13**	rs4814176	SM(OH).C22:1	20:12907398	*SPTLC3*	T/C	0.36	0.03(0.035)	4.53E-01	-0.07(0.009)	9.11E-17	10	1.22	0.87
**13**	rs4814176	SM(OH) C24:1	20:12907398	*SPTLC3*	T/C	0.36	0.24(0.035)	4.29E-12	0.09(0.013)	2.85E-11	9	0.86	-0.90
**14**	rs5746636	Pro	22:17276301	*PRODH*	T/G	0.24	-0.31(0.039)	1.89E-15	-0.32(0.034)	5.05E-21	2	0.11	0.03
						Only uGAS							
**15**	rs2943644	C5:1-DC	2:226754586	*LOC646736*	C/T	0.68	0.32(0.042)	3.99E-14	0.09(0.022)	3.97E-05	5	0.56	-1.08

						Only cGAS							
**16**	rs1374804	Gly	3:127391188	*ALDH1L1*	A/G	0.64	0.20(0.036)	1.46E-08	0.21(0.029)	3.65E-13	3	0.17	0.05
**17**	rs4862429	PC ae C42:5	4:186006834	*ACSL1*	T/C	0.31	0.02(0.037)	6.63E-01	-0.06(0.008)	1.15E-12	8	1.34	1.09
**18**	rs603424	C16:1	10:102065469	*PKD2L1*	A/G	0.80	0.16(0.042)	9.00E-05	0.14(0.018)	9.32E-14	9	0.71	-0.15
**19**	rs2657879	Gln	12:55151605	*GLS2*	G/A	0.21	-0.24(0.042)	2.65E-08	-0.27(0.030)	5.88E-18	5	0.29	0.10
**20**	rs17112944	C6:1	14:27179297	*LOC728755*	A/G	0.90	-0.28(0.059)	1.98E-06	-0.21(0.031)	3.74E-11	9	0.54	-0.26

Notes: The best SNP-metabolite pair is shown for each locus. chr:pos refers to the physical position of the SNP; EAF, effect allele frequency; beta (se), the estimated effect and standard error of the SNP; effA/refA, effect allele/reference allele; *P*-value, the *p*-value of the additive model; Gene, the most likely (according to DEPICT) associated gene in the region; N_{cov}, the number of covariates used in cGAS; Noise/Pleiotropic, the values of noise and pleiotropic components of the log-ratio of cGAS and uGAS T^2 statistics.

Discussion

We report a new "trait-centric" approach for analyzing genetic determinants of multivariate "omics" traits by performing a network-based conditional genetic association analysis (cGAS). In the context of metabolomics, for each trait we selected a set of other metabolites to be used as covariates in our genetic association analysis. The selection of covariates can be either mechanistic (e.g., based on known biological relationships between traits of interest) or data-driven (e.g., based on partial correlations). Importantly, this approach can use either individual-level or summary-level data. We first mathematically compared the power of conditional and standard single-trait genetic association analyses (univariate genetic association, uGAS), and we identified scenarios in which these analyses are expected to produce different results; next, we applied cGAS to 151 metabolomics traits (Biocrates panel) in a large (n=1784 individuals) population-based KORA cohort.

We found that the log-ratio between the cGAS and uGAS test statistic can be decomposed in a "noise" component (which depends on residual variance of the trait and is always positive) and a "pleiotropic" component. The pleiotropic component is negative in cases in which genetically induced covariance (between the trait of interest and the trait used as the covariate) and the residual covariance have the same sign (i.e., act in the same direction). The pleiotropic component is positive in cases in which the genetically induced covariance and residual covariance act in opposite directions.

Should one expect that genetically induced and residual covariance act in the same or opposite directions? In essence, this is a question about the architecture of pleiotropy: is a pleiotropic genetic variant expected to induce the same covariance as would be induced by non-genetic mechanisms? It has been reasoned that in randomly bred populations, the genetic correlations are expected to arise primarily from pleiotropic gene action [22]. In such populations, a study and comparison of genetic and environmental correlations—while unable to provide single-variant resolution—may provide a general notion of what may be expected for consistency/anti-consistency between genetic and residual covariance. Based on published literature, Cheverud [23] and Roff [24] concluded that genetic and environmental correlations normally have both the same sign and the same magnitude. This pattern is particularly clear for morphologic traits, as opposed to life-history traits (see [25] for review and additional references). These observations are consistent with recent studies of genetic correlations between complex human polygenic traits (see [26]).

Consequently, for complex traits, one may expect that the sign of the pleiotropic component of the log-ratio between the cGAS and uGAS tests (individual summands in the second term of the equation (2)) is generally negative. It should be noted, though, that a negative sign for the pleiotropic component does not necessarily indicate higher power of the uGAS, as the noise component (the first term in equation (2)) may still dominate the log-ratio between the cGAS and uGAS tests. This will happen, for example, when $\hat{\rho}_{cg}$ (the effect of the genotype on the covariate) is small while $\hat{\beta}_{yc}$ (partial residual regression between the trait and covariate) is relatively large, thereby reducing $\hat{\sigma}_c^2$.

Nevertheless, in the case of metabolomic traits, genetic and environmental sources do not necessarily generate consistent covariance. Moreover, for a given locus that affects the activity of an enzyme involved in a biochemical reaction, the unexpected inconsistency between genetically induced covariance and residual covariance may not be so unexpected after all. Indeed, consider an allele associated with an increased activity of an enzyme that converts substrate A into product B. One would expect that the levels of A and B are positively correlated; one would also expect that the allele is positively correlated with the level of product B and negatively correlated with the level of substrate A. This is precisely the scenario that yields a positive value for the second term in Eq. (1), thus providing an additional increase in power above and beyond the power provided by the first term in Eq. (1) (noise reduction).

Our empirical investigation of real data on the genetic association between the genome and metabolites confirmed the existence of both scenarios. An extreme example of concordance between genetic covariance and residual covariance is provided by the effects of rs8396 on C10, with C8 and C12 used as covariates (see Figure 2B). The *ETFDH* gene, which was prioritized by DEPICT software (see Materials and Methods) as the best candidate in this region (with a false-discover rate <5%), encodes the enzyme electron transfer flavoprotein (ETF) dehydrogenase, which plays a role in mitochondrial fatty acid oxidation. During this process, the acyl group is transferred from a long chain acylcarnitine to a long-chain acetyl-CoA, which is then catabolized. ETF dehydrogenase participates in the catabolic process by transferring electrons from acyl-CoA dehydrogenase to the oxidative phosphorylation pathway. Thus, the *ETFDH* gene should affect all forms of long-chain acylcarnitines in the same way, and we can expect that the pleotropic effect of this gene on the acylcarnitines in our example (C8, C10, C12, etc.) will be unidirectional. The presence of unidirectional genetic effects and the positive correlation between these acylcarnitines makes the second term in Eq. (2) negative, which determines that—in this situation—univariate GAS has more power than cGAS.

An empirical example of discordance between genetically induced covariance and residual covariance is provided by the effects of the SNP rs174547 on lyso-PC a C20:4, with lyso-PC a

C20:3 used as a covariate. This SNP exhibits opposite correlations with lyso-PC a C20:4 and lyso-PC a C20:3, resulting in negative genetically induced covariance between these traits. At the same time, the residual correlation between these traits is positive, resulting in steep increase in the power of conditional analysis. In this region, the *FADS1/2/3* gene cluster is an attractive candidate, providing the detected model with biological relevance. The *FADS1* gene encodes the enzyme fatty acid desaturase 1, whereas the two traits differ by only one double bond. Thus, this example mimics perfectly the biochemical scenario in which we would expect a conditional analysis to have increased power.

The trait-centric methods considered here provide an attractive framework to identify and study direct genetic effects on a trait of interest. Conditional analysis is an attractive option in cases in which we wish to clearly interpret the results in terms of the effect of the genotype on a particular trait. Such specific interpretation may be important when comparing genetic association results obtained for our trait of interest with results obtained for other traits (e.g., using the methods described in [27–29]). It should be noted, though, that a trait-centric approach is not intended to maximize the power of identifying genes that affect metabolomics as a whole. Such a gene-centric view would favor analysis using joint—and not conditional—modeling of sets of traits. Such an approach can maintain power across a wide range of scenarios, including the scenario of concordance between genetically induced and residual covariance [13]. In this gene-centric framework, other formulations of conditional analysis have also been proposed [30] in order to specifically increase power of gene identification by selecting covariates that—using our terminology—affect the “noise reduction” component of the model while avoiding the problems associated with the pleiotropic component.

The proper selection of sets of biologically related traits is extremely important for the conditional genetic association analysis method described here, as well as for multivariate methods that model the joint effects of genotype on an ensemble of traits. Here, we considered two alternative approaches—knowledge based and data-driven—to finding the networks of related traits, with a subnetwork centered around a trait of interest used as the analyzed set. In principle, in the context of analyzed metabolomics data, the knowledge-based network approach has slightly higher power in the context of trait-centric genetic association analysis. However, we believe that our analysis revealed that both approaches are suboptimal. The knowledge-based network reconstruction has many advantages, but it may be somewhat unpractical, as our biochemical knowledge is still relatively incomplete. Secondly, by reconstructing the network while relying only on current knowledge, we may be missing new knowledge that may be revealed by the data. Finally, by including neighbors that are based only on biochemical information, we may miss covariance induced by technical confounders; adjusting for this may increase the power of analysis

[30]. Learning the network from the same data that were used for genetic analysis has the disadvantages of potentially ignoring existing knowledge and being sensitive to sample size. Finally, we note that the total observed correlation between metabolites is determined by the balance between genetic and environmental sources of covariance; it is possible to imagine a situation in which total correlation is smaller than one or more of its components, and our analysis provides examples of such a situation. We may speculate that—ideally—one should use a method that allows one to combine prior knowledge and new information obtained from the data, thereby allowing the simultaneous learning of the structure of dependencies between different metabolites and between the metabolites and the genome. Such learning from the data while allowing for the incorporation of previous knowledge (e.g., biochemical relations between traits) might be achieved (for example, by applying a machine-learning approach that allows for differential shrinkage). It is also important to note that the proper application of such an approach would require the availability of vast samples of data, thereby allowing for separate training, validation, verification, testing, and replication of detected dependencies and associations.

Materials and Methods

KORA study

The KORA study (Cooperative Health Research in the region of Augsburg) is a series of population-based studies in the region of Augsburg in Southern Germany [31]. KORA F4 is a follow-up survey (conducted from 2006 through 2008) of the baseline KORA S4 survey, which was conducted from 1999 through 2001. All study protocols were approved by the ethics committee of the Bavarian Medical Chamber, and all participants provided written informed consent.

The concentration of 163 metabolites were measured in 3061 serum samples obtained from KORA F4 participants using flow injection electrospray ionization tandem mass spectrometry and the AbsoluteIDQ p150 Kit (Biocrates Life Sciences AG, Innsbruck, Austria) [32]. After applying quality control screening, a total of 151 metabolite measurements were used in our analysis. Details regarding the methods and quality control of the metabolite measurements, as well as details regarding the metabolite nomenclature, have been published previously [32]. The nomenclature for the metabolites in this study is provided in **Supplementary Table S3**.

Genotyping was performed using the Affymetrix 6.0 SNP array (534,174 SNP markers after quality control), with further imputation using HapMap2 (release 22) as a reference panel, resulting in a total of 1,717,498 SNPs (for details, see ref. [33]). Both the metabolite concentrations and genotype were available for 1785 participants in the KORA F4 study.

Statistical analysis

Partial correlation coefficients and their *p*-values were calculated using the "ppcor" package [34] in R. Graphical representations were generated using the "ggm" [35] package in R. Consistent with previous studies [18], we considered a partial regression coefficient to be significant at $p < 0.01/(151*150/2)$ (i.e., $p<8.83 \times 10^{-7}$).

For the GWAS analysis, we used OmicABEL software [36]. Prior to GWAS, all traits were first adjusted for the participant's sex, age, and batch effect; subsequently, the residual traits were transformed using an inverse-normal transformation [37]. The genotypes from the KORA F4 cohort were used. Only SNPs that had a call rate ≥0.95, $R^2 \geq 0.3$, Hardy–Weinberg equilibrium (HWE) $p \geq 10^{-6}$, and MAF ≥0.1 (1,717,498 SNPs in total) were included in the analysis. The genomic control method was used to correct for any possible inflation of the test statistics. The genomic control [38] lambda value for all traits was between 1.00 and 1.03.

In a specific analysis (i.e., cGAS or uGAS), we defined independent loci as groups of genome-wide significant associations that were separated by at least 500 kb or were located on

different chromosomes. The strongest association (i.e., the association with the lowest *p*-value) was selected to represent this locus. The cGAS and uGAS results were considered to reflect different loci if the strongest associations were in loci that were separated by at least 500 kb. The threshold for the genome-wide significance for 151 traits was set to $p=5 \times 10^{-8}/151$ (i.e., $p=3.31 \times 10^{-10}$).

When partitioning the log(cGAS/uGAS) test statistics into the noise and pleiotropic components (see Eq. (2) and **Figure 1**), we used all known loci that were significant in either the cGAS or uGAS analysis (see Table 1). If a locus included two SNPs associated with different traits, we included both associations during partitioning. If a locus included two SNPs associated with the same trait, to be conservative we included only the SNP with the lower uGAS *p*-value during partitioning. After partitioning, we determined whether the value of the pleiotropic and noise components were statistically different using the paired-samples Wilcoxon test. For comparing the chi-square test results for the two methods, for each locus we selected the largest chi-square value for selected SNP among all analyzed traits. If locus had two SNPs we selected for each method only the largest chi-square value.

The code for BN-cGAS and GGM-cGAS analyses, and the code for producing the summary tables and graphs, was implemented in R and is available as a workflow from CodeOcean, a cloud-based computational reproducibility platform.

In silico functional annotation

We conducted functional annotation for our findings. To prioritize genes in associated regions, gene set enrichment, and tissue/cell-type enrichment analyses, we used DEPICT (Data-driven Expression-Prioritized Integration for Complex Traits) software [39] (release 140721) with the following settings: flag_loci = 1; flag_genes = 1; flag_genesets = 1; flag_tissues = 1; param_ncores = 2; and further manual annotation (h37 assembly). All 27 SNPs (clustered in 20 loci) identified by cGAS or uGAS (see **Table 2**) were included in the analysis. If more than one gene was annotated for a SNP by DEPICT, we selected the gene with the lowest nominal DEPICT P-value. In most cases, the results of manual annotation matched the annotation results using DEPICT annotation (see **Supplementary Note 2**). In addition, we looked up each SNP using the Phenoscanner [40] database to check whether it was previously reported to be associated with metabolic traits at $p<5 \times 10^{-8}$ and proxy $r^2 <0.7$.

Additional Files

Supplementary Note 1 – cGAS using summary level data

Supplementary Note 2 – Literature search for loci identified by cGAS and uGAS

Supplementary Tables

- Supplementary Table S1 – BN- cGAS and GGM- cGAS for 105 metabolites
- Supplementary Table S2 – GGM-cGAS and uGAS for 151 metabolites
- Supplementary Table S3 - List of metabolites measured using the AbsoluteIDQ p150 Kit

Supplementary Figures

- Supplementary Figure S1 – Partial correlations network
- Supplementary Figure S2 – Manhattan plots for cGAS and uGAS for 151 metabolites
- Supplementary Figure S3 – Comparison of effect estimates and their standard errors for SNPs from Table 2

Abbreviations

GWAS – genome-wide association study

cGAS – conditional GWAS

uGAS – univariate GWAS (trait-by-trait)

BN-cGAS – cGAS based on biochemical networks

GGM-cGAS – Gaussian Graphical Modeling cGAS based on partial correlations network

Acknowledgments

We thank Athina Spilopoulou and Felix Agakov for helpful discussions. We also thank Alexander Zlobin and Alexander Grishenko for help preparing the tables and figures in the manuscript, and we thank Sophie Molnos for help with data management. We thank the reviewers - Simina Boca and Tim Ebbels—for their very helpful input.

Funding

The KORA study was initiated and financed by the Helmholtz Center Munich – German Research Center for Environmental Health, which is funded by the German Federal Ministry of Education and Research (BMBF) and by the State of Bavaria. Furthermore, the KORA study was supported by the Munich Center of Health Sciences (MC-Health), Ludwig Maximilian University of Munich, as part of the LMUinnovativ project.

This work was supported by the European Union FP7 framework project Pain-Omics (grant

number 602736).

SS was supported by the Russian Ministry of Science and Education under the 5-100 Excellence Programme. YA and YT were supported by the Federal Agency of Scientific Organisations via the Institute of Cytology and Genetics (project number 0324-2018-0017).

Authors Contributions

YT, CG, and YA designed and supervised the study; PC, CP, JA, KG, and RW-S collected the data; CG and KS contributed data for the analysis; YT, OZ, and SS analyzed the data; YT, YA, CG, OZ, JK, and KS discussed and interpreted the results; YT, OZ, CG, and YA wrote the manuscript. All authors contributed to and approve the final version of the manuscript.

Availability of Data and Materials

The code produced in relation to this work and all summary statistics and association data that are necessary to reproduce our results are stored in the GigaDB database [41] and on the CodeOcean platform as a reproducible workflow [42]. The informed consent given by the KORA study participants does not cover the posting of participant-level phenotype or genotype data in public databases. However, the KORA data are available upon request from KORA-gen (https://www.helmholtz-muenchen.de/en/kora/index.html). Requests can be submitted online and are subject to approval by the KORA board.

Competing Interests

Y. Aulchenko is the founder and co-owner of PolyOmica, a private research organization that specializes in computational and statistical (gen)omics.

References

1. Visscher PM, Brown MA, McCarthy MI, Yang J. Five Years of GWAS Discovery. Am J Hum Genet [Internet]. 2012;90:7–24. Available from: http://linkinghub.elsevier.com/retrieve/pii/S0002929711005337

2. Ritchie MD, Holzinger ER, Li R, Pendergrass SA, Kim D. Methods of integrating data to uncover genotype–phenotype interactions. Nat Rev Genet [Internet]. 2015;16:85–97. Available from: http://www.nature.com/doifinder/10.1038/nrg3868

3. van der Sijde MR, Ng A, Fu J. Systems genetics: From GWAS to disease pathways. Biochim Biophys Acta - Mol Basis Dis [Internet]. 2014;1842:1903–9. Available from: http://linkinghub.elsevier.com/retrieve/pii/S0925443914001124

4. Hicks AA, Pramstaller PP, Johansson A, Vitart V, Rudan I, Ugocsai P, et al. Genetic determinants of circulating sphingolipid concentrations in European populations. PLoS Genet [Internet]. 2009 [cited 2013 Dec 19];5:e1000672. Available from: http://www.pubmedcentral.nih.gov/articlerender.fcgi?artid=2745562&tool=pmcentrez&rendertype=abstract

5. Suhre K, Shin S-Y, Petersen A-K, Mohney RP, Meredith D, Wägele B, et al. Human metabolic individuality in biomedical and pharmaceutical research. Nature [Internet]. 2011 [cited 2013 Dec 19];477:54–60. Available from: http://www.pubmedcentral.nih.gov/articlerender.fcgi?artid=3832838&tool=pmcentrez&rendertype=abstract

6. Inouye M, Ripatti S, Kettunen J, Lyytikäinen L-P, Oksala N, Laurila P-P, et al. Novel Loci for metabolic networks and multi-tissue expression studies reveal genes for atherosclerosis. Visscher PM, editor. PLoS Genet [Internet]. 2012;8:e1002907. Available from: http://dx.plos.org/10.1371/journal.pgen.1002907

7. Draisma HHM, Pool R, Kobl M, Jansen R, Petersen A-K, Vaarhorst AAM, et al. Genome-wide association study identifies novel genetic variants contributing to variation in blood metabolite levels. Nat Commun [Internet]. England; 2015;6:7208. Available from: http://www.ncbi.nlm.nih.gov/pubmed/26068415

8. Kettunen J, Demirkan A, Würtz P, Draisma HHMM, Haller T, Rawal R, et al. Genome-wide study for circulating metabolites identifies 62 loci and reveals novel systemic effects of LPA. Nat Commun [Internet]. 2016;7:11122. Available from: http://www.ncbi.nlm.nih.gov/pubmed/27005778%5Cnhttp://www.nature.com/doifinder/10.1038/ncomms11122

9. Cichonska A, Rousu J, Marttinen P, Kangas AJ, Soininen P, Lehtimäki T, et al. metaCCA: summary statistics-based multivariate meta-analysis of genome-wide association studies using canonical correlation analysis. Bioinformatics [Internet]. 2016;32:1981–9. Available from: http://www.ncbi.nlm.nih.gov/pubmed/27153689

10. Stephens M. A unified framework for association analysis with multiple related phenotypes. PLoS One [Internet]. 2013;8:e65245. Available from: http://www.ncbi.nlm.nih.gov/pubmed/23861737

11. O'Reilly PF, Hoggart CJ, Pomyen Y, Calboli FCF, Elliott P, Jarvelin M-R, et al. MultiPhen: joint model of multiple phenotypes can increase discovery in GWAS. PLoS One [Internet]. 2012 [cited 2014 Sep 20];7:e34861. Available from: http://www.pubmedcentral.nih.gov/articlerender.fcgi?artid=3342314&tool=pmcentrez&rendertype=abstract

12. Galesloot TE, van Steen K, Kiemeney LALM, Janss LL, Vermeulen SH. A comparison of multivariate genome-wide association methods. PLoS One [Internet]. 2014 [cited 2014 Sep 20];9:e95923. Available from: http://www.ncbi.nlm.nih.gov/pubmed/24763738

13. Shen X, Klarić L, Sharapov S, Mangino M, Ning Z, Wu D, et al. Multivariate discovery and replication of five novel loci associated with Immunoglobulin G N-glycosylation. Nat Commun [Internet]. 2017;8:447. Available from: http://www.nature.com/articles/s41467-017-00453-3

14. Schaid DJ, Tong X, Larrabee B, Kennedy RB, Poland GA, Sinnwell JP. Statistical Methods for Testing Genetic Pleiotropy. Genetics [Internet]. 2016;204:483–97. Available from: http://www.genetics.org/cgi/doi/10.1534/genetics.116.189308

15. Deng Y, Pan W. Conditional analysis of multiple quantitative traits based on marginal GWAS summary statistics. Genet Epidemiol [Internet]. 2017;41:427–36. Available from: http://www.ncbi.nlm.nih.gov/pubmed/28464407

16. Cox DR, Hinkley D V. Theoretical statistics. 1974. London, Chapman Hall. 1:511.

17. Smith GD, Ebrahim S. "Mendelian randomization": can genetic epidemiology contribute to understanding environmental determinants of disease? Int J Epidemiol [Internet]. 2003;32:1–22. Available from: http://www.ncbi.nlm.nih.gov/pubmed/12689998

18. Krumsiek J, Suhre K, Illig T, Adamski J, Theis FJ. Gaussian graphical modeling reconstructs pathway reactions from high-throughput metabolomics data. BMC Syst Biol [Internet]. BioMed Central Ltd; 2011 [cited 2013 May 23];5:21. Available from: http://www.pubmedcentral.nih.gov/articlerender.fcgi?artid=3224437&tool=pmcentrez&rendertype=abstract

19. Tsepilov YA, Shin S-Y, Soranzo N, Spector TD, Prehn C, Adamski J, et al. Nonadditive Effects of Genes in Human Metabolomics. Genetics [Internet]. 2015;200:707–18. Available from: http://www.genetics.org/cgi/doi/10.1534/genetics.115.175760

20. Xie W, Wood AR, Lyssenko V, Weedon MN, Knowles JW, Alkayyali S, et al. Genetic variants associated with glycine metabolism and their role in insulin sensitivity and type 2 diabetes. Diabetes [Internet]. 2013;62:2141–50. Available from: http://www.ncbi.nlm.nih.gov/pubmed/23378610

21. Shin S-Y, Fauman EB, Petersen A-K, Krumsiek J, Santos R, Huang J, et al. An atlas of genetic influences on human blood metabolites. Nat Genet [Internet]. 2014 [cited 2014 May 12];46:543–50. Available from: http://www.nature.com/doifinder/10.1038/ng.2982

22. Falconer DS, Mackay TFC. Introduction to Quantitative Genetics (4th Edition) [Internet]. 4th ed. Pearson; 1996. Available from: http://www.amazon.com/exec/obidos/redirect?tag=citeulike07-20&path=ASIN/0582243025

23. Cheverud JM. A COMPARISON OF GENETIC AND PHENOTYPIC CORRELATIONS. Evolution [Internet]. 1988;42:958–68. Available from: http://www.ncbi.nlm.nih.gov/pubmed/28581166

24. Roff DA. The estimation of genetic correlations from phenotypic correlations: a test of Cheverud's conjecture. Heredity (Edinb) [Internet]. 1995;74:481–90. Available from: http://www.nature.com/articles/hdy199568

25. Lynch M, Walsh B, others. Genetics and analysis of quantitative traits. Sinauer Sunderland, MA; 1998.

26. Bulik-Sullivan B, Finucane HK, Anttila V, Gusev A, Day FR, Loh P-R, et al. An atlas of genetic correlations across human diseases and traits. Nat Genet. Nature Publishing Group; 2015;47:1236–41.

27. Zhu Z, Zhang F, Hu H, Bakshi A, Robinson MR, Powell JE, et al. Integration of summary data from GWAS and eQTL studies predicts complex trait gene targets. Nat Genet [Internet]. 2016;48:481–7. Available from: http://www.nature.com/doifinder/10.1038/ng.3538%5Cnhttp://www.ncbi.nlm.nih.gov/pubmed/27019110

28. Pickrell JK, Berisa T, Liu JZ, Ségurel L, Tung JY, Hinds DA. Detection and interpretation of shared genetic influences on 42 human traits. Nat Genet. 2016;019885.

29. Giambartolomei C, Vukcevic D, Schadt EE, Franke L, Hingorani AD, Wallace C, et al. Bayesian Test for Colocalisation between Pairs of Genetic Association Studies Using Summary Statistics. Williams SM, editor. PLoS Genet [Internet]. 2014;10:e1004383. Available from: http://dx.plos.org/10.1371/journal.pgen.1004383

30. Aschard H, Guillemot V, Vilhjalmsson B, Patel CJ, Skurnik D, Ye CJ, et al. Covariate selection for association screening in multiphenotype genetic studies. Nat Genet [Internet]. 2017;49:1789–

95. Available from: http://www.nature.com/doifinder/10.1038/ng.3975
31. Wichmann H-E, Gieger C, Illig T. KORA-gen--resource for population genetics, controls and a broad spectrum of disease phenotypes. Gesundheitswesen [Internet]. 2005 [cited 2013 Jun 6];67 Suppl 1:S26-30. Available from: http://www.ncbi.nlm.nih.gov/pubmed/16032514
32. Illig T, Gieger C, Zhai G, Römisch-Margl W, Wang-Sattler R, Prehn C, et al. A genome-wide perspective of genetic variation in human metabolism. Nat Genet [Internet]. Nature Publishing Group; 2010 [cited 2013 May 23];42:137–41. Available from: http://www.ncbi.nlm.nih.gov/pubmed/20037589
33. Kolz M, Johnson T, Sanna S, Teumer A, Vitart V, Perola M, et al. Meta-analysis of 28,141 individuals identifies common variants within five new loci that influence uric acid concentrations. PLoS Genet [Internet]. 2009 [cited 2013 May 30];5:e1000504. Available from: http://www.pubmedcentral.nih.gov/articlerender.fcgi?artid=2683940&tool=pmcentrez&rendertype=abstract
34. Kim S. ppcor: An R Package for a Fast Calculation to Semi-partial Correlation Coefficients. Commun Stat Appl Methods [Internet]. 2015;22:665–74. Available from: http://www.csam.or.kr/journal/view.html?doi=10.5351/CSAM.2015.22.6.665
35. Marchetti GM. Independencies Induced from a Graphical Markov Model after Marginalization and Conditioning: The R Package ggm. J Stat Softw [Internet]. 2006;15. Available from: http://www.jstatsoft.org/v15/i06/
36. Fabregat-Traver D, Sharapov SZ, Hayward C, Rudan I, Campbell H, Aulchenko Y, et al. High-Performance Mixed Models Based Genome-Wide Association Analysis with omicABEL software. F1000Research [Internet]. 2014;3:200. Available from: http://f1000research.com/articles/3-200/v1
37. Beasley TM, Erickson S, Allison DB. Rank-based inverse normal transformations are increasingly used, but are they merited? Behav Genet [Internet]. 2009 [cited 2013 Nov 7];39:580–95. Available from: http://www.pubmedcentral.nih.gov/articlerender.fcgi?artid=2921808&tool=pmcentrez&rendertype=abstract
38. Devlin B, Roeder K. Genomic control for association studies. Biometrics [Internet]. 1999 [cited 2013 Jun 5];55:997–1004. Available from: http://www.ncbi.nlm.nih.gov/pubmed/11315092
39. Pers TH, Karjalainen JM, Chan Y, Westra H-J, Wood AR, Yang J, et al. Biological interpretation of genome-wide association studies using predicted gene functions. Nat Commun [Internet]. 2015;6:5890. Available from: http://www.ncbi.nlm.nih.gov/pubmed/25597830
40. Staley JR, Blackshaw J, Kamat MA, Ellis S, Surendran P, Sun BB, et al. PhenoScanner: a database of human genotype–phenotype associations. Bioinformatics [Internet]. 2016;32:3207–9. Available from: http://bioinformatics.oxfordjournals.org/lookup/doi/10.1093/bioinformatics/btw373
41. Tsepilov YA, Sharapov SZ, Zaytseva OO, Krumsek J, Prehn C, Adamski J, et al. Supporting data for "A network-based conditional genetic association analysis of the human metabolome". GigaScience Database 2018. http://dx.doi.org/10.5524/100507
42. Tsepilov Y, Sharapov S, Aulchenko Y A network-based conditional genetic association analysis of the human metabolome [Source Code]. https://doi.org/10.24433/CO.3b5ea77b-859a-4db9-af44-b8b6aeb88664.v2

Figure 1

Click here to access/download;Figure;F1.png

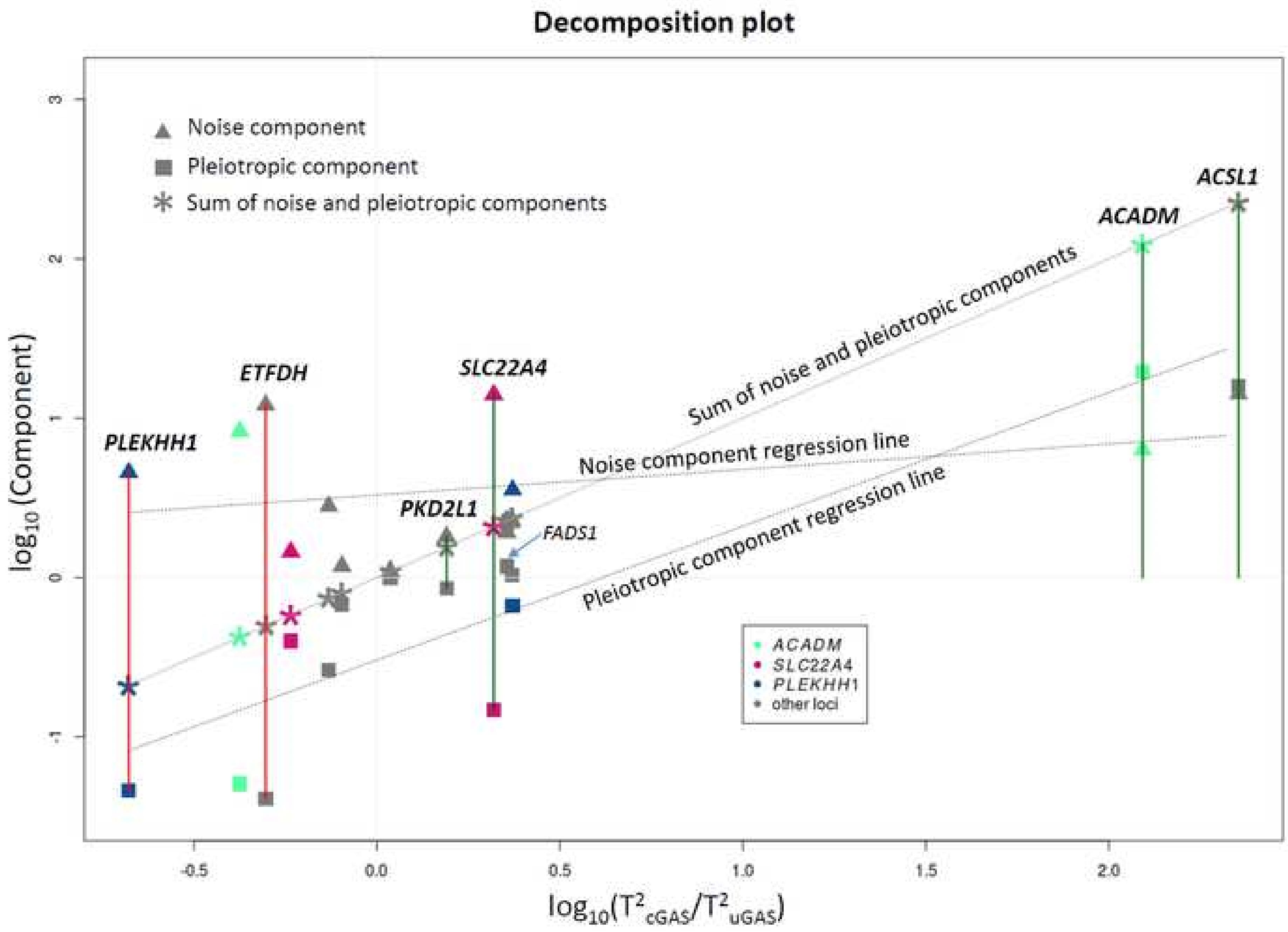

Figure 2

Click here to access/download;Figure;F2.png

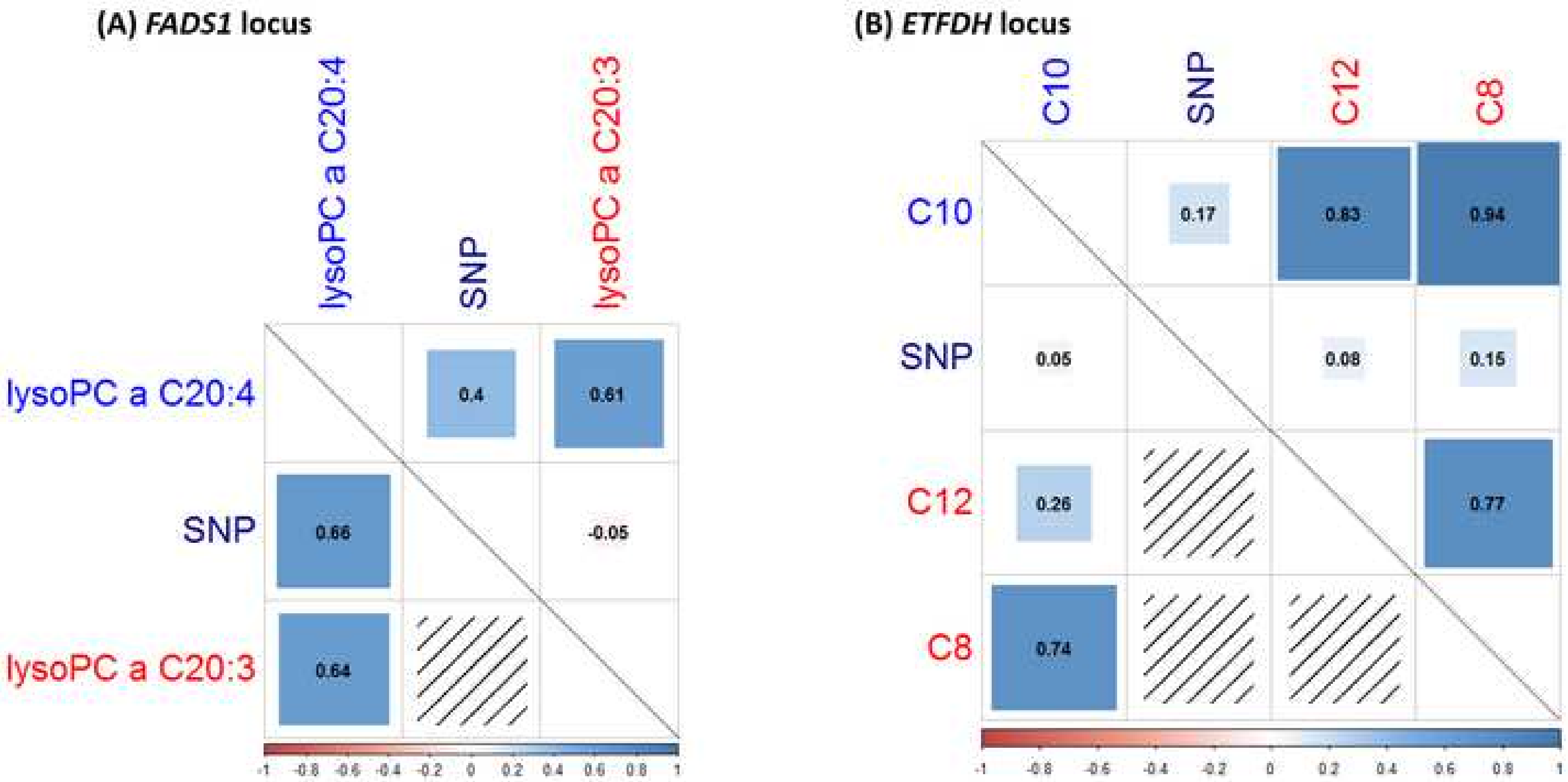

SF

Click here to access/download
Supplementary Material
Supplementary Figures.docx

SN1

Click here to access/download
Supplementary Material
Supplementary Note 1.docx

SN2

Click here to access/download

Supplementary Material

Supplementary Note 2.docx

ST1

Click here to access/download
Supplementary Material
Supplementary Table 1.xlsx

ST2

Click here to access/download
Supplementary Material
Supplementary Table 2.xlsx

ST3

Click here to access/download
Supplementary Material
Supplementary Table 3.docx
