# Supplementary material for: A network-based conditional genetic association analysis of the human metabolome
Source: Gigascience. 2018 Nov 29;7(12):giy137. doi: 10.1093/gigascience/giy137 (PMC6287100; doi:10.1093/gigascience/giy137)
Supplement: Supplemental Files [file giy137_supplemental_files.zip › Supplementary Figures.docx]

**Supplementary Figure S1.**


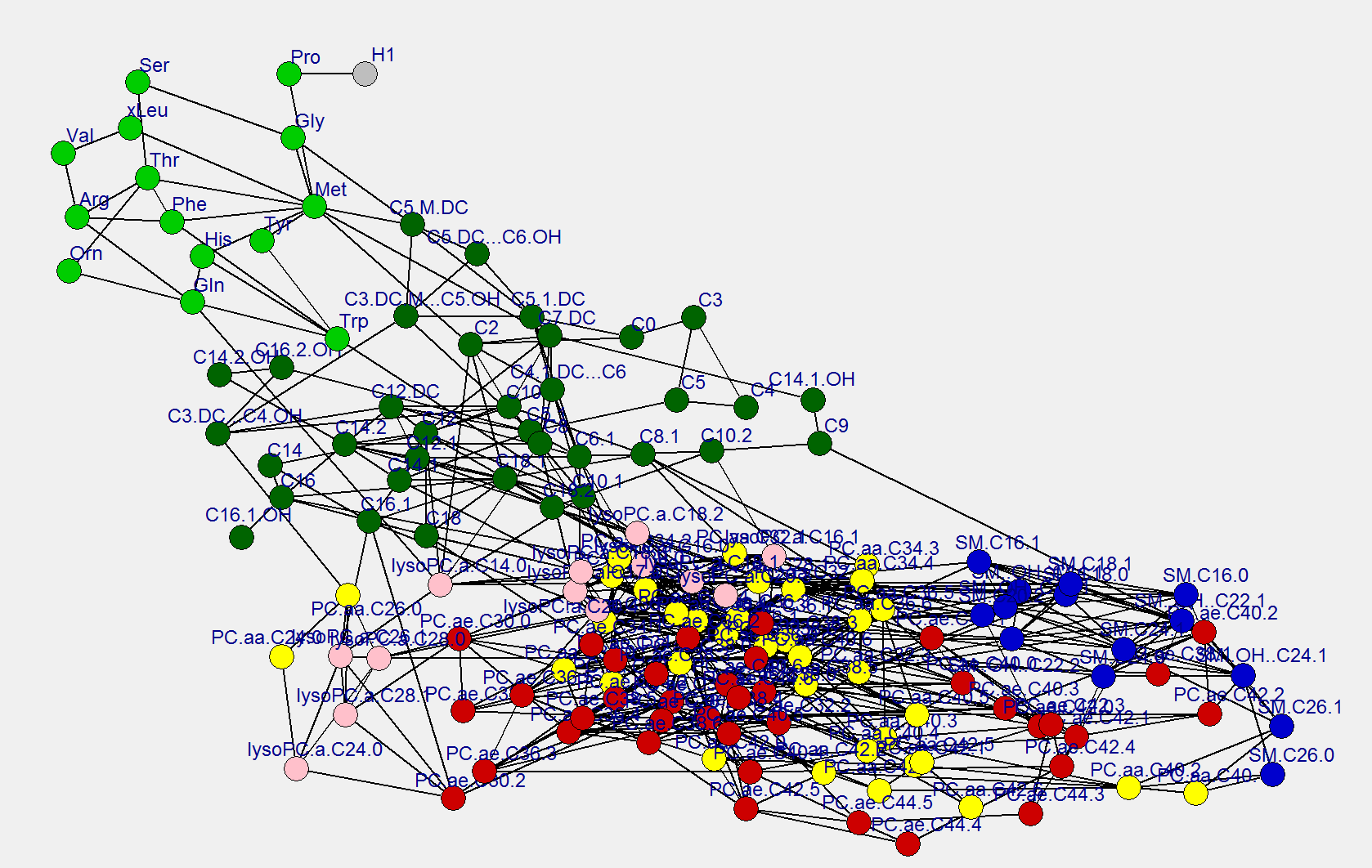
**Supplementary Figure S1. GGM network of 151 metabolites identified using Biocrates.** Each node represents a single metabolite. The color of each symbol represents the class of metabolite as follows: red, PC.ae; blue, SM; yellow, PC.aa; dark green, AC; light green, AA; gray, H; and pink, lyso-PC.

**Supplementary Figure S2.**

**
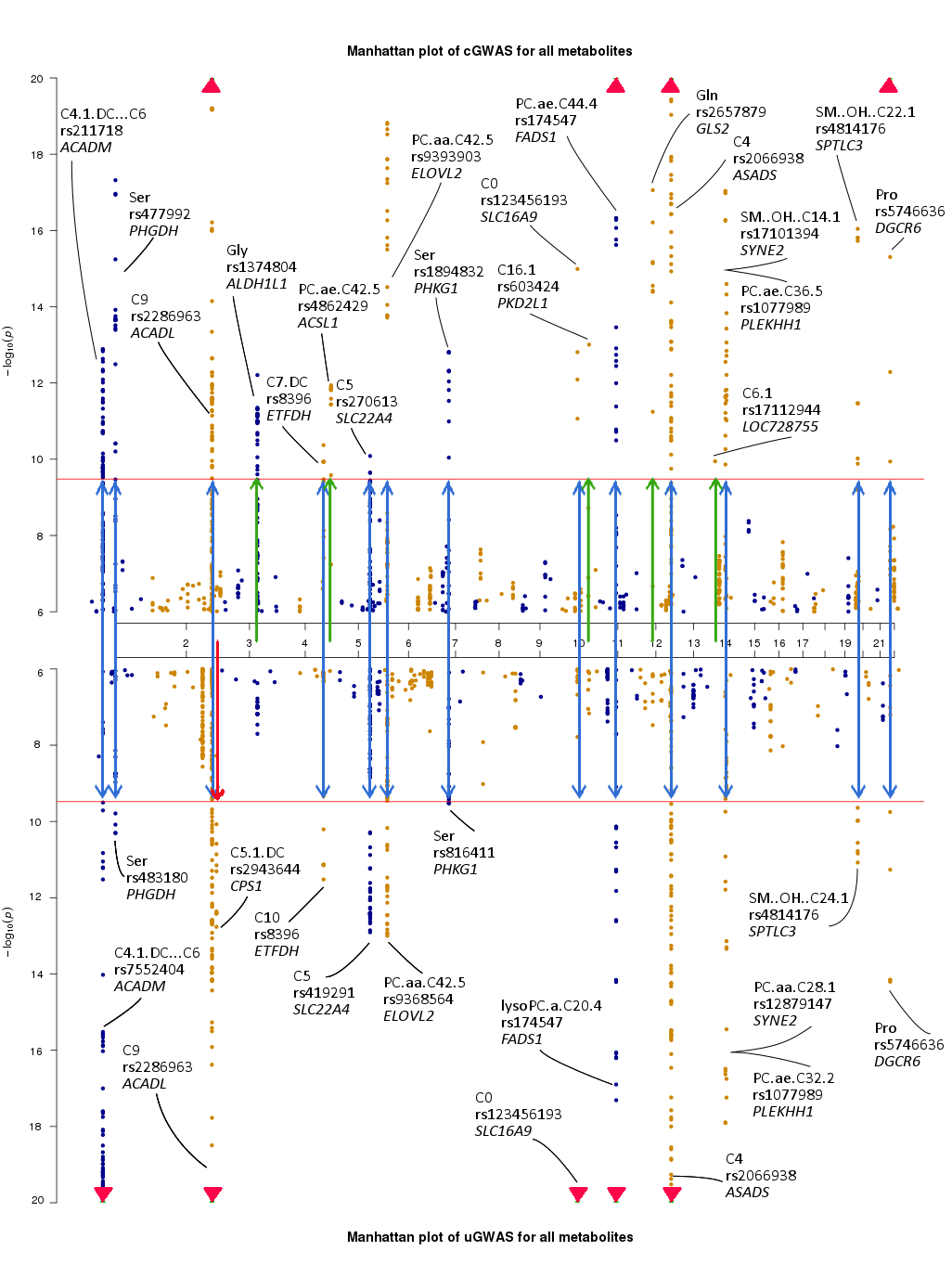
**

**Supplementary Figure S2. Miami plot depicting the cGAS (upper part) and uGAS (lower part) results.** All plots were restricted to maximal -log_10_ *p*-value of 20, and all data points above this value are represented by a red triangle. The horizontal red lines represent the genome-wide significance level threshold (5E-8/151). The blue/orange colors correspond to SNPs on different chromosomes. The blue double arrows indicate loci that were significantly associated with at least one metabolite using both cGAS and uGAS; the green arrows indicate loci detected only by cGAS, and the red arrow indicates the locus that was detected only by uGAS. For each locus, the annotation is provided specifying the strongest associated metabolite and SNP, as well as the name of the most likely candidate gene (prioritized using DEPICT software).

**Supplementary Figure S3.**


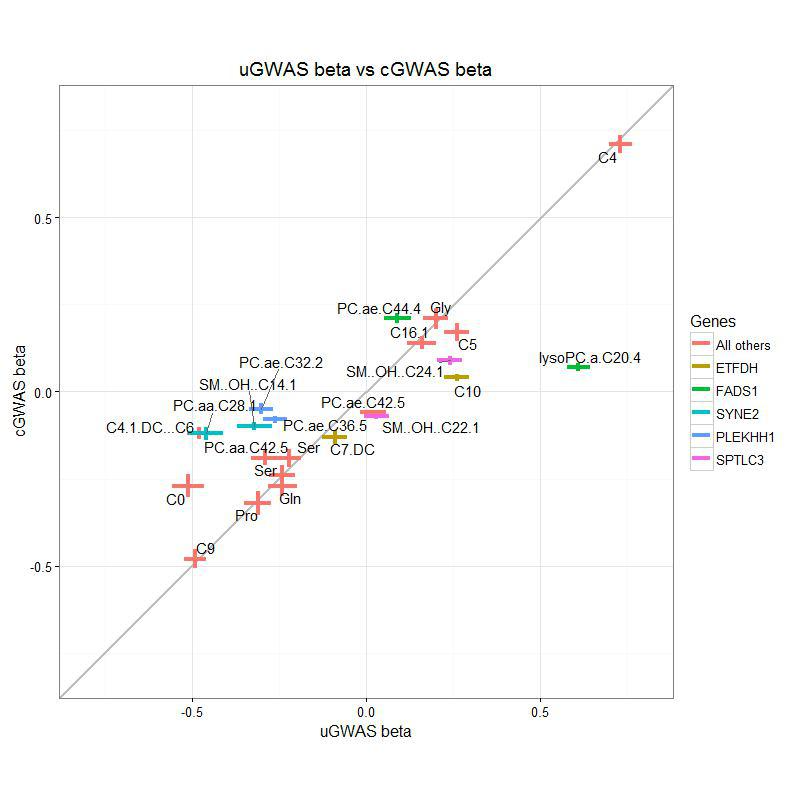


**Supplementary Figure S3. Comparison of genetic effect estimates between uGAS and GGM-cGAS.** Each locus-tagging SNP-trait pair is represented as a dot with whiskers that correspond to the standard error of the estimate obtained from GGM-cGAS (vertical whiskers) and uGAS (horizontal whiskers). Five loci (*ETFDH, FADS1, PLEKHH1, SPTLC3, SYNE2*) in which the strongest trait-SNP association pair differs between the two methods are presented as two dots of the same color.
