# Supplementary material for: A network-based conditional genetic association analysis of the human metabolome
Source: Gigascience. 2018 Nov 29;7(12):giy137. doi: 10.1093/gigascience/giy137 (PMC6287100; doi:10.1093/gigascience/giy137)
Supplement: Supplemental Files [file giy137_supplemental_files.zip › Supplementary Note 1.docx]

**Supplementary Note 1.**

**Summary level conditional analysis and implementation.**

Consider a regression model

$y=Xb+e$ (1)

where *y* is an *n* × 1 vector of phenotypes, with *n* being the sample size, *X* is the design *n* x (*m*+1) matrix (which may be partitioned into the column-vector of genotype *g*, and sub-matrix of *k* covariates, *C*, X=[g|C]), *b* is a vector of joint effects, and *e* is the residual noise.

This problem has a well-known least-squares solution:

$b={(X'X)}^{-1}X^{'}y$ and $var\left( b \right)={\sigma_{j}^{2}(X'X)}^{-1}$ (2)

where $\sigma_{j}^{2}$ is the residual variance of *y*. It should be noted that

$X^{'}X=M*(n-k-2)$, where *M* is the covariance matrix of *X,* and $X^{'}y=cov\left( X,y \right)*\left( n-1 \right)$, where $cov\left( X,y \right)$ is the vector of covariances between all predictors (*X*) and *y*. Thus, this solution can be rewritten in terms of the covariance matrix (*X* and *y* are not necessarily centered) as follows:

$b=M^{-1}\times cov(X,y)$; $\sigma_{j}^{2}=var\left( y \right)-b\times cov\left( X,y \right)$ and $var\left( b \right)=diag(\frac{{\sigma_{j}^{2}*M}^{-1}}{n-k-2})$ (3)

Thus, if we know the variance-covariance matrix $M$ and $cov\left( X,y \right)$, we have a solution to the problem in Eq. (1). If we have access to the results of univariate GWAS analyses for all involved traits, we can estimate and/or approximate these variables, which consist of several parts: covariance between the genotype and each trait, the covariance between all traits, and the variance of these variables.

The covariance between genotype *g* and trait-of-interest *C_i_* (or *y_i_*) can be estimated using the well-known solution:

$cov\left( g,C_{i} \right)=\beta_{g,C_{i}}*var(g)$ (4)

where $\beta_{g,C_{i}}$ is effect of genotype on the trait *C_i_* from a univariate GWAS. Variance of *g* can be estimated directly from the data, or from a reference sample, or it can be approximated under Hardy-Weinberg equilibrium assumption using $var\left( g \right)=2p(1-p)$, where *p* is the allele frequency.

The covariance matrix between all traits (all *C* and *y*) can also be estimated from univariate GWAS statistics using methods described by Stephens et al. [1], which uses Z-statistics from GWAS for a large number of non-associated SNPs and approximates the correlation between traits as the correlation between Z‑statistics for these traits.

Assuming the above, we can then estimate all parts of matrix *M* and $cov\left( X,y \right)$. This gives us the solution to problem (3).

In this study, we used variances of genotypes estimated directly from the data and a variance-covariance matrix between all phenotypes estimated from the data. Because this study was based on one cohort, all solutions are exact (i.e., they do not differ from conventional analysis of individual-level data). In principle, using the described assumptions, this method can be easily applied to the results of a GWAS meta-analysis of omics-based data.

**REFERENCES:**

[1] Stephens, M. (2013). A unified framework for association analysis with multiple related phenotypes. PloS One, 8(7), e65245. http://doi.org/10.1371/journal.pone.0065245
