# Supplementary material for: A network-based conditional genetic association analysis of the human metabolome
Source: Gigascience. 2018 Nov 29;7(12):giy137. doi: 10.1093/gigascience/giy137 (PMC6287100; doi:10.1093/gigascience/giy137)
Supplement: Supplemental Files [file giy137_supplemental_files.zip › Supplementary Note 2.docx]

**Supplementary Note 2.**

**Functional annotation and manual gene prioritization**

We conducted a functional annotation of the novel loci. We independently performed manual gene prioritization for all genes in the associated region by its likely functional role and prioritization using DEPICT software (a software package for prioritizing genes in associated regions, gene set enrichment, and tissue/cell-type enrichment analyses). All 27 SNPs clustered in the 20 loci identified by cGAS and uGAS (see Table 2 in the main text) were included in our analysis. If several genes were proposed for a SNP by DEPICT, we selected the gene with the lowest nominal DEPICT P-value. In most cases, the results of our manual annotation matched the results of DEPICT annotation. All results are presented in Table below, including references (PMID) for published associations. For the new loci detected by cGAS and for all loci in which we manual annotation differed from DEPICT, we gave the annotation with an explanation of the possible underling biological mechanism.

**Twenty loci found identified using cGAS and uGAS.** The best SNP-metabolite pair is shown for each locus. chr:pos corresponds to the physical position of the SNP; EAF: effect allele frequency; beta (se): the estimated effect and standard error of the SNP; effA/refA: effect allele/reference allele; *P*-value: the *p*-value of the additive model; Gene, the most likely associated gene in the region (according to DEPICT); Consensus gene, the best candidate gene based on manual expert annotation; Ncvrts, number of covariates for cGAS.

|  |  |  |  |  |  |  |  |  | **uGAS** | | **cGAS** | | |  |  |  |
| --- | --- | --- | --- | --- | --- | --- | --- | --- | --- | --- | --- | --- | --- | --- | --- | --- |
| **Locus** | **SNP** | **Metabolite** | **chr:pos** | **Gene *** | **Consensus gene** | **effA/refA** | **PMID for previous publication**** | **EAF** | **beta (se)** | ***P*-value** | **beta (se)** | ***P*-value** | **Ncvrts** |  |  |  |
|  |  | **uGAS & cGAS** | | | | | | | | | | | |  |  |  |
| 1 | rs211718 | C4.1.DC...C6 | 1:75,879,263 | *ACADM* | *ACADM* | T/C | 24816252] | 0.30 | -0.48 (0.034) | 4.64E-42 | -0.13 (0.017) | 2.00E-13 | 7 |  |  |  |
| 1 | rs7552404 | C4.1.DC...C6 | 1:75,908,534 | *ACADM* | *ACADM* | G/A | [24816252] | 0.30 | -0.48 (0.034) | 3.10E-42 | -0.12 (0.017) | 3.25E-13 | 7 |  |  |  |
| 2 | rs483180 | Ser | 1:120,069,028 | *PHGDH* | *PHGDH* | G/C | [24816252] [21886157] | 0.30 | -0.24 (0.037) | 3.34E-11 | -0.24 (0.028) | 1.50E-17 | 2 |  |  |  |
| 2 | rs477992 | Ser | 1:120,059,099 | *PHGDH* | *PHGDH* | A/G | [24816252] [21886157] | 0.70 | 0.24 (0.037) | 5.15E-11 | 0.24 (0.028) | 5.82E-18 | 2 |  |  |  |
| 3 | rs2286963 | C9 | 2:210,768,295 | *ACADL* | *ACADL* | G/T | [20037589] | 0.63 | -0.49 (0.032) | 1.10E-49 | -0.48 (0.027) | 1.48E-67 | 3 |  |  |  |
| 4 | rs8396 | C10 | 4:159,850,267 | *ETFDH* | *ETFDH* | C/T | [24816252] | 0.71 | 0.26 (0.037) | 2.02E-12 | 0.04 (0.010) | 1.49E-05 | 8 |  |  |  |
| 4 | rs8396 | C7.DC | 4:159,850,267 | *ETFDH* | *ETFDH* | C/T | [20037589] | 0.71 | -0.09 (0.037) | 1.67E-02 | -0.13 (0.020) | 3.29E-11 | 8 |  |  |  |
| 5 | rs419291 | C5 | 5:131,661,254 | *SLC22A4* | *SLC22A4* | T/C | [24816252] | 0.38 | 0.26 (0.035) | 7.03E-14 | 0.17 (0.026) | 2.28E-10 | 3 |  |  |  |
| 5 | rs270613 | C5 | 5:131,668,482 | *SLC22A4* | *SLC22A4* | A/G | [24816252] | 0.61 | -0.26 (0.035) | 7.93E-14 | -0.17 (0.026) | 8.48E-11 | 3 |  |  |  |
| 6 | rs9393903 | PC.aa.C42.5 | 6:11,150,895 | *ELOVL2* | *ELOVL2* | A/G | [20037589] | 0.75 | 0.29 (0.039) | 2.19E-13 | 0.18 (0.020) | 4.51E-19 | 6 |  |  |  |
| 6 | rs9368564 | PC.aa.C42.5 | 6:11,168,269 | *ELOVL2* | *ELOVL2* | G/A | [20037589] | 0.25 | -0.29 (0.039) | 1.14E-13 | -0.19 (0.021) | 7.84E-19 | 6 |  |  |  |
| 7 | rs816411 | Ser | 7:56,138,983 | *PHKG1* | *PHKG1* | C/T | [24816252] | 0.51 | -0.22 (0.034) | 2.15E-10 | -0.19 (0.026) | 5.16E-13 | 2 |  |  |  |
| 7 | rs1894832 | Ser | 7:56,144,740 | *PHKG1* | *PHKG1* | C/T | [24816252] | 0.51 | 0.21 (0.034) | 3.23E-10 | 0.19 (0.026) | 1.69E-13 | 2 |  |  |  |
| 8 | rs12356193 | C0 | 10:61,083,359 | *SLC16A9* | *SLC16A9* | G/A | [20037589, 24816252] | 0.17 | -0.51 (0.046) | 1.84E-27 | -0.27 (0.034) | 9.72E-16 | 3 |  |  |  |
| 9 | rs174547 | lysoPC.a.C20.4 | 11:61,327,359 | *FADS2 (FADS1)* | *FADS1* | C/T | [27005778, 24823311] | 0.70 | 0.61 (0.033) | 1.44E-69 | 0.07 (0.011) | 1.41E-10 | 9 |  |  |  |
| 9 | rs174556 | PC.ae.C44.4 | 11:61,337,211 | *FADS2 (FADS1)* | *FADS1* | T/C | [22359512] | 0.27 | 0.09 (0.038) | 1.55E-02 | 0.21 (0.014) | 3.16E-46 | 3 |  |  |  |
| 10 | rs2066938 | C4 | 12:119,644,998 | *ACADS* | *ACADS* | G/A | [24816252] | 0.27 | 0.73 (0.033) | 5.87E-94 | 0.71 (0.025) | 1.31E-151 | 2 |  |  |  |
| 11 | rs12879147 | PC.aa.C28.1 | 14:63,297,349 | *SYNE2* | *SYNE2* | A/G | [22359512,19798445  ] | 0.85 | -0.46 (0.050) | 2.07E-19 | -0.12 (0.019) | 5.94E-11 | 14 |  |  |  |
| 11 | rs17101394 | SM..OH..C14.1 | 14:63,302,139 | *SYNE2* | *SYNE2* | A/G | [22359512,19798445  ] | 0.83 | -0.32 (0.050) | 1.00E-10 | -0.10 (0.011) | 1.17E-17 | 7 |  |  |  |
| 12 | rs1077989 | PC.ae.C36.5 | 14:67,045,575 | *TMEM229B (PLEKHH1)* | *PLEKHH1* | C/A | [20037589] | 0.46 | -0.26 (0.034) | 3.42E-14 | -0.08 (0.010) | 8.25E-16 | 10 |  |  |  |
| 12 | rs1077989 | PC.ae.C32.2 | 14:67,045,575 | *TMEM229B (PLEKHH1)* | *PLEKHH1* | C/A | [20037589] | 0.46 | -0.30 (0.034) | 2.23E-18 | -0.05 (0.016) | 1.31E-03 | 6 |  |  |  |
| 13 | rs4814176 | SM..OH..C22.1 | 20:12,907,398 | *RP5-1069C8.2 lincRNA (SPTLC3)* | *SPTLC3* | T/C | [20037589] | 0.36 | 0.03 (0.035) | 4.51E-01 | -0.07 (0.009) | 1.10E-16 | 10 |  |  |  |
| 13 | rs4814176 | SM..OH..C24.1 | 20:12,907,398 | *RP5-1069C8.2 lincRNA (SPTLC3)* | *SPTLC3* | T/C | [20037589] | 0.36 | 0.24 (0.035) | 5.40E-12 | 0.09 (0.013) | 3.04E-11 | 9 |  |  |  |
| 14 | rs5746636 | Pro | 22:17,276,301 | *PRODH (DGCR6)* | *PRODH* | T/G | [24816252] | 0.24 | -0.31 (0.039) | 3.00E-15 | -0.32 (0.034) | 1.91E-20 | 2 |  |  |  |
|  |  | **Only uGAS** | | | | | | | | | | | |  |  | **Only uGAS** |
| 15 | rs2943644 | C5.1.DC | 2:226,754,586 | *AC068138.1 lincRNA, LOC646736* | *LOC646736* | C/T | - | 0.68 | 0.32 (0.042) | 5.14E-14 | 0.09 (0.022) | 3.58E-05 | 5 |  |  |  |
|  |  | **Only cGAS** | | | | | | | | | | | |  |  | **Only cGAS** |
| 16 | rs1374804 | Gly | 3:127,391,188 | *ALDH1L1-AS2 (ALDH1L1)* | *ALDH1L1* | A/G | [24816252, 27005778] | 0.64 | 0.20 (0.036) | 1.88E-08 | 0.21 (0.030) | 8.08E-13 | 3 |  |  |  |
| 17 | rs4862429 | PC.ae.C42.5 | 4:186,006,834 | *ACSL1* | *ACSL1* | T/C | [20037589] | 0.31 | 0.02 (0.037) | 6.62E-01 | -0.06 (0.008) | 1.25E-12 | 8 |  |  |  |
| 18 | rs603424 | C16.1 | 10:102,065,469 | *PKD2L1* | *PKD2L1* | A/G | [21886157] | 0.80 | 0.16 (0.042) | 9.51E-05 | 0.14 (0.018) | 1.32E-13 | 9 |  |  |  |
| 19 | rs2657879 | Gln | 12:55,151,605 | *GLS2* | *GLS2* | G/A | [27005778] | 0.21 | -0.24 (0.042) | 2.82E-08 | -0.27 (0.031) | 9.37E-18 | 5 |  |  |  |
| 20 | rs17112944 | C6.1 | 14:27,179,297 | *LINC00645 (LOC728755)* | *LOC728755* | A/G | - | 0.90 | -0.28 (0.059) | 2.09E-06 | -0.21 (0.032) | 1.38E-10 | 9 |  |  |  |

* SNPs were annotated using DEPICT software; the results of manual annotation are shown in parentheses when they differ from the result obtained using DEPICT.

** We tested each SNP with Phenoscanner [1] if that SNP was associated with metabolic traits with a *p*-value <5E-8 in the previously published association studies

# Annotation

**rs1077989**

For the region tagged by rs1077989, which is associated with phosphatidylcholine acyl‐alkyl C32:2, *TMEM229B* was prioritized by DEPICT. The other potential candidate in this region is *PLEKHH1*. Both genes have an obscure function. *PLEKHH1* encodes a protein containing a pleckstrin homology domain, and this gene was previous reported to be associated with plasma [phospholipid levels](https://www.ebi.ac.uk/gwas/search?query=Phospholipid%20levels%20(plasma))[2]; in addition, the corresponding protein has been proposed to participate in protein-lipid interactions and to affect membrane structure [3]. Although rs1077989 was reported by Demirkan et al. to be connected with *PLEKHH1* [2], the gene mapped to this SNP by NCBI is *TMEM229B*, which encodes for a transmembrane protein with an unknown function.

**rs4814176**

DEPICT software mapped two long intergenic non-coding RNA (lincRNA) genes, *AC068138.1* and *RP5-1069C8.2*, to rs2943644 (associated with glutaconylcarnitine levels) and rs4814176 (associated with hydroxysphingomyeline C24:1 levels), respectively. LincRNAs are believed to regulate protein synthesis by interacting with the mRNA. Some mRNAs are interact with *AC068138.1* lincRNA [4], although we cannot connect them directly with the studied metabolic traits. Thus, we used the nearest coding region, *LOC646736*.

**rs5746636**

The *DGCR6* gene was mapped manually for this SNP, and the *PRODH* gene was annotated by DEPICT. *DGCR6* has been suggested to play a role in DiGeorge syndrome [5]. The *PRODH* gene encodes proline dehydrogenase (oxidase) 1, an enzyme directly involved in the degradation pathway of the amino acid proline. Thus, the *PRODH* gene seems to be a more logical choice in this case, as rs5746636 was found to be associated with proline concentration in our study.

**rs17104556**

The SNP rs17104556 was found to be associated with phosphatidylcholine acyl‐alkyl C44:4 concentration by cGAS. In the set of SNPs discovered using the uGAS method (see Main Text Table 1), manual annotation and annotation with DEPICT were similar. However, because gene prioritization using DEPICT depends on the set of SNPs being analyzed, a somewhat different annotation was observed for the SNP set discovered using cGAS (see Main Text Table 2). Manually, we mapped the *FADS1* gene and the DEPICT-proposed FADS2 gene (the *p*-values for the *FADS1* and *FADS2* genes in the cGAS SNP set were similar: 7.42E-4 and 2.48E-4, respectively). These genes encode fatty acid desaturase 1 and fatty acid desaturase 2, respectively. Both genes are located in a cluster of *FADS* genes on chromosome 11 and participate in the same metabolic pathway of fatty acids, and both genes were previously reported to be associated with plasma cholesterol levels [6] and phospholipid levels [7].

**New SNPs detected only by cGAS**

Biological annotation of the hits detected by cGAS is particularly interesting.

**rs17112944**

This SNP was found to be associated with the concentration of hexenoylcarnitine. Manually, the uncharacterized genetic locus LOC728755 was mapped for this association, whereas DEPICT software mapped lincRNA 645, which overlaps with the above-mentioned locus.

**rs4862429**

This SNP is associated with phosphatidylcholine diacyl C42:5. DEPICT software mapped the MIR3945 host gene, which encodes a long non-coding RNA. Manually, we mapped the *ACSL1* gene for this hit, which encodes acyl-CoA synthetase long-chain family member 1. This enzyme activates long-chain and very-long-chain fatty acids to form acyl-CoA, a key step in fatty acid metabolism. Gain-of-function and loss-of-function studies indicate that this enzyme’s activity is important for channeling fatty acids into different pathways for synthesizing complex lipids synthesis [8].

**rs1374804**

This SNP is associated with glycine levels. Manually, the *ALDH1L1* gene was mapped for this association, and *ALDH1L1* antisense RNA 2 was proposed by DEPICT. *ALDH1L1* encodes the L1 member of the aldehyde dehydrogenase 1 family, which catalyzes the reduction of NADP+ to for NADPH and is responsible for formate oxidation *in vivo*. Previously, an association was found between a polymorphism in the *ALDH1L1* gene and the glycine-to-serine ratio [9].

**rs603424**

This SNP is associated with hexadecenoylcarnitine C16:1 levels, and the *PKD2L1* gene was mapped both manually and with DEPICT software to this association. This gene encodes polycystic kidney disease 2-like protein 1, also known as transient receptor potential polycystic 3 (TRPP3), which functions as a calcium-regulated nonselective cation channel and is believed to be involved in cell-cell and cell-matrix interactions. Previously, a polymorphism in this gene was found to be associated with plasma phospholipid [2], palmitic (C16:0) acid, and palmitoleic acid (16:1n-7) levels [10].

**rs2657879**

This SNP is associated with glutamine levels, and the *GLS2* gene was mapped both manually and with DEPICT software to this association. This gene encodes glutaminase 2, a mitochondrial enzyme that catalyzes the hydrolysis of glutamine. Previous studies reported an association between a polymorphism in this gene and the plasma glutamine-to-histidine ratio in humans [11].

**REFERENCES:**

1. Staley JR, Blackshaw J, Kamat MA, Ellis S, Surendran P, Sun BB, et al. PhenoScanner: a database of human genotype–phenotype associations. Bioinformatics. 2016;32: 3207–3209. doi:10.1093/bioinformatics/btw373

2. Demirkan A, van Duijn CM, Ugocsai P, Isaacs A, Pramstaller PP, Liebisch G, et al. Genome-wide association study identifies novel loci associated with circulating phospho- and sphingolipid concentrations. PLoS Genet. 2012;8: e1002490. doi:10.1371/journal.pgen.1002490

3. Ma AD, Brass LF, Abrams CS. Pleckstrin Associates with Plasma Membranes and Induces the Formation of Membrane Projections: Requirements for Phosphorylation and the NH 2 -terminal PH Domain. J Cell Biol. 1997;136: 1071–1079. doi:10.1083/jcb.136.5.1071

4. Imig J, Brunschweiger A, Brümmer A, Guennewig B, Mittal N, Kishore S, et al. miR-CLIP capture of a miRNA targetome uncovers a lincRNA H19-miR-106a interaction. Nat Chem Biol. Nature Publishing Group, a division of Macmillan Publishers Limited. All Rights Reserved.; 2015;11: 107–14. doi:10.1038/nchembio.1713

5. Gao W, Higaki T, Eguchi-Ishimae M, Iwabuki H, Wu Z, Yamamoto E, et al. DGCR6 at the proximal part of the DiGeorge critical region is involved in conotruncal heart defects. Hum Genome Var. Nature Publishing Group; 2015;2: 15004. doi:10.1038/hgv.2015.4

6. Teslovich TM, Musunuru K, Smith A V, Edmondson AC, Stylianou IM, Koseki M, et al. Biological, clinical and population relevance of 95 loci for blood lipids. Nature. Nature Publishing Group; 2010;466: 707–13. doi:10.1038/nature09270

7. Lemaitre RN, Tanaka T, Tang W, Manichaikul A, Foy M, Kabagambe EK, et al. Genetic loci associated with plasma phospholipid n-3 fatty acids: a meta-analysis of genome-wide association studies from the CHARGE Consortium. PLoS Genet. Public Library of Science; 2011;7: e1002193. doi:10.1371/journal.pgen.1002193

8. Mashek DG, Li LO, Coleman RA. Long-chain acyl-CoA synthetases and fatty acid channeling. Future Lipidol. 2007;2: 465–476. doi:10.2217/17460875.2.4.465

9. Xie W, Wood AR, Lyssenko V, Weedon MN, Knowles JW, Alkayyali S, et al. Genetic variants associated with glycine metabolism and their role in insulin sensitivity and type 2 diabetes. Diabetes. 2013;62: 2141–50. doi:10.2337/db12-0876

10. Wu JHY, Lemaitre RN, Manichaikul A, Guan W, Tanaka T, Foy M, et al. Genome-wide association study identifies novel loci associated with concentrations of four plasma phospholipid fatty acids in the de novo lipogenesis pathway: results from the Cohorts for Heart and Aging Research in Genomic Epidemiology (CHARGE) consortiu. Circ Cardiovasc Genet. NIH Public Access; 2013;6: 171–83. doi:10.1161/CIRCGENETICS.112.964619

11. Shin S-Y, Fauman EB, Petersen A-K, Krumsiek J, Santos R, Huang J, et al. An atlas of genetic influences on human blood metabolites. Nat Genet. 2014;46: 543–50. doi:10.1038/ng.2982
