# Supplementary material for: A network-based conditional genetic association analysis of the human metabolome
Source: Gigascience. 2018 Nov 29;7(12):giy137. doi: 10.1093/gigascience/giy137 (PMC6287100; doi:10.1093/gigascience/giy137)
Supplement: Supplemental Files [file giy137_supplemental_files.zip › Supplementary Table 3.docx]

**Supplemental table 3: List of metabolites measured with the AbsoluteIDQ® p150 Kit**

**GAC, Helmholtz Zentrum München**

| **Acylcarnitines (41)** | | | | | |
| --- | --- | --- | --- | --- | --- |
| C0 | Carnitine | | C10:1 | Decenoylcarnitine | |
| C2 | Acetylcarnitine | | C10:2 | Decadienylcarnitine | |
| C3 | Propionylcarnitine | | C12 | Dodecanoylcarnitine | |
| C3:1 | Propenoylcarnitine | | C12:1 | Dodecenoylcarnitine | |
| C3-OH | Hydroxypropionylcarnitine | | C12-DC | Dodecanedioylcarnitine | |
| C4 | Butyrylcarnitine | | C14 | Tetradecanoylcarnitine | |
| C4:1 | Butenoylcarnitine | | C14:1 | Tetradecenoylcarnitine | |
| C4-OH (C3-DC) | Hydroxybutyrylcarnitine | | C14:1-OH | Hydroxytetradecenoylcarnitine | |
| C5 | Valerylcarnitine | | C14:2 | Tetradecadienylcarnitine | |
| C5:1 | Tiglylcarnitine | | C14:2-OH | Hydroxytetradecadienylcarnitine | |
| C5:1-DC | Glutaconylcarnitine | | C16 | Hexadecanoylcarnitine | |
| C5-DC (C6-OH) | Glutarylcarnitine  (Hydroxyhexanoylcarnitine) | | C16:1 | Hexadecenoylcarnitine | |
| C5-M-DC | Methylglutarylcarnitine | | C16:1-OH | Hydroxyhexadecenoylcarnitine | |
| C5-OH (C3-DC-M) | Hydroxyvalerylcarnitine  (Methylmalonylcarnitine) | | C16:2 | Hexadecadienylcarnitine | |
| C6 (C4:1-DC) | Hexanoylcarnitine  (Fumarylcarnitine) | | C16:2-OH | Hydroxyhexadecadienylcarnitine | |
| C6:1 | Hexenoylcarnitine | | C16-OH | Hydroxyhexadecanoylcarnitine | |
| C7-DC | Pimelylcarnitine | | C18 | Octadecanoylcarnitine | |
| C8 | Octanoylcarnitine | | C18:1 | Octadecenoylcarnitine | |
| C8:1 | Octenoylcarnitine | | C18:1-OH | Hydroxyoctadecenoylcarnitine | |
| C9 | Nonanoylcarnitine | | C18:2 | Octadecadienylcarnitine | |
| C10 | Decanoylcarnitine | |  |  | |
|  | | | | | |
| **Amino Acids (14)** | | | | | |
| Arg | Arginine | | Pro | Proline | |
| Gln | Glutamine | | Ser | Serine | |
| Gly | Glycine | | Thr | Threonine | |
| His | Histidine | | Trp | Tryptophan | |
| Met | Methionine | | Tyr | Tyrosine | |
| Orn | Ornithine | | Val | Valine | |
| Phe | Phenylalanine | | xLeu | Leucine/Isoleucine | |
|  | | | | | |
| **Monosaccharides (1)** | | | | | |
| Sum of Hexoses (including Glucose) | | | | | |
|  | | | | | |
| **Glycerophospholipids (92)** | | | | | |
| lysoPC a C6:0 | | PC aa C32:3 | PC aa C40:6 | | PC ae C38:1 |
| lysoPC a C14:0 | | PC aa C34:1 | PC aa C42:0 | | PC ae C38:2 |
| lysoPC a C16:0 | | PC aa C34:2 | PC aa C42:1 | | PC ae C38:3 |
| lysoPC a C16:1 | | PC aa C34:3 | PC aa C42:2 | | PC ae C38:4 |
| lysoPC a C17:0 | | PC aa C34:4 | PC aa C42:4 | | PC ae C38:5 |
| lysoPC a C18:0 | | PC aa C36:0 | PC aa C42:5 | | PC ae C38:6 |
| lysoPC a C18:1 | | PC aa C36:1 | PC aa C42:6 | | PC ae C40:0 |
| lysoPC a C18:2 | | PC aa C36:2 | PC ae C30:0 | | PC ae C40:1 |
| lysoPC a C20:3 | | PC aa C36:3 | PC ae C30:1 | | PC ae C40:2 |
| lysoPC a C20:4 | | PC aa C36:4 | PC ae C30:2 | | PC ae C40:3 |
| lysoPC a C24:0 | | PC aa C36:5 | PC ae C32:1 | | PC ae C40:4 |
| lysoPC a C26:0 | | PC aa C36:6 | PC ae C32:2 | | PC ae C40:5 |
| lysoPC a C26:1 | | PC aa C38:0 | PC ae C34:0 | | PC ae C40:6 |
| lysoPC a C28:0 | | PC aa C38:1 | PC ae C34:1 | | PC ae C42:0 |
| lysoPC a C28:1 | | PC aa C38:3 | PC ae C34:2 | | PC ae C42:1 |
| PC aa C24:0 | | PC aa C38:4 | PC ae C34:3 | | PC ae C42:2 |
| PC aa C26:0 | | PC aa C38:5 | PC ae C36:0 | | PC ae C42:3 |
| PC aa C28:1 | | PC aa C38:6 | PC ae C36:1 | | PC ae C42:4 |
| PC aa C30:0 | | PC aa C40:1 | PC ae C36:2 | | PC ae C42:5 |
| PC aa C30:2 | | PC aa C40:2 | PC ae C36:3 | | PC ae C44:3 |
| PC aa C32:0 | | PC aa C40:3 | PC ae C36:4 | | PC ae C44:4 |
| PC aa C32:1 | | PC aa C40:4 | PC ae C36:5 | | PC ae C44:5 |
| PC aa C32:2 | | PC aa C40:5 | PC ae C38:0 | | PC ae C44:6 |
|  | | | | | |
| **Sphingolipids (15)** | | | | | |
| SM (OH) C14:1 | | SM C18:0 | SM (OH) C22:1 | | SM (OH) C24:1 |
| SM C16:0 | | SM C18:1 | SM (OH) C22:2 | | SM C26:0 |
| SM C16:1 | | SM C20:2 | SM C24:0 | | SM C26:1 |
| SM (OH) C16:1 | | SM C22:3 | SM C24:1 | |  |
