# Supplementary material for: A network-based conditional genetic association analysis of the human metabolome
Source: Gigascience. 2018 Nov 29;7(12):giy137. doi: 10.1093/gigascience/giy137 (PMC6287100; doi:10.1093/gigascience/giy137)
Supplement: response_to_reviewer_comments_original_submission.pdf [file giy137_response_to_reviewer_comments_original_submission.pdf]

Author's Response To Reviewer Comments

Close

Dear Hans,

We would like to thank you and reviewers for helpful comments and suggestions. Please find attached our point-by-point answers below. We revised our manuscript accordingly. We hope that you and the reviewers will find the revised manuscript suitable for publication in GigaScience.

We must note that while implementing the reproducible workflow and answering the comments and suggestions from the reviewers we have detected and corrected several inconsistencies between our actual current numbers and few numbers reported in the manuscript. Our conclusions were not affected by these occasional changes.

Yours Sincerely, also on behalf of other authors,
prof. Yurii Aulchenko and dr. Yakov Tsepilov

Editor’s comments:
1) In particular, reviewer 1 highlights a couple of issues that require more clarity and statistical rigor, for example regarding the two data models, cGAS vs. uGAS.

We carefully revised manuscript according to the comments of Reviewer #1 and made necessary clarifications and corrections (see our answers below).

2) Along similar lines, reviewer 2 feels the text is hard to follow and should be revised to be more accessible, also to readers who don't have specific expertise in this area (please see the two reports below for details).

We extended the text of the manuscript with additional explanations and clarifications according to Reviewer’s suggestions (see answers below).

3) I also agree with point #7 of reviewer that a reproducible workflow (with code and summary data) would be most helpful and would add value to your manuscript. Providing data and code in reproducible and re-usable formats is one of our major goals at GigaScience. We recently started a collaboration with the code sharing platform "Code Ocean" (https://codeocean.com/). Code submitted to Code Ocean is assigned a Digital Object Identifier, and via a DOI it can be easily and stably referenced in your GigaScience article. To learn more about Code Ocean integration, please read our blog post … Please consider to provide your workflow and data in an easily executable format. I feel this could also help to answer some of the reviewers' concerns. If you have further questions regarding Code Ocean integration please don't hesitate to get in touch.

Thank you for this suggestion! We designed reproducible workflow and made it available through the CodeOcean platform, as suggested. The link to the workflow is: https://codeocean.com/2018/04/02/a-network-based-conditional-genetic-association-analysis-of-the-human-metabolome/

Reviewer reports:

Reviewer #1:
1) One major issue I see is the way the authors claim that a cGAS would usually have higher power. In general, power is defined in relation to a specific null hypothesis and the null hypothesis is different for the uGAS versus the cGAS. For example, the higher power may be at the cost of a higher type I error/FWER. Furthermore, all these claims are made based on a single data analysis, whereas more convincing and illustrative arguments generally would also include simulations where the true data-generating mechanisms is known - for example, if the data follows the uGAS models, how does using the cGAS impact the type I error/FWER as well as the power?

We agree that, strictly speaking, the null hypotheses for uGAS and cGAS are different. uGAS test compares a model where mean, genetic effect, and residual variance are estimated with the (null) model where genetic effect is set to zero, while all other parameters are estimated. cGAS test compares a model where mean, genetic effect, effects of covariates, and residual variance are estimated with the (null) model where genetic effect is set to zero, while all other parameters are estimated. However, for both uGAS and cGAS tests, the difference between the number of parameters estimated under the null and under the alternative is one, and, according to Neyman-Pearson lemma, both uGAS and cGAS tests should -- under the null hypothesis -- follow the chi-square distribution with 1 degree of freedom. The fact that each of the tests is distributed as chi-squared with 1df under the null can be supported by observation that both for uGAS and GGM-cGAS lambda GC varied from 0.98 to 1.05. Further, to support our reasoning that both uGAS and cGAS tests are distributed in the same manner under the null, we compared the Genomic control inflation factor Lambda between uGWAS and GGM-cGAS, for all 151 metabolites. The difference between these was not significant (t-test p-value=0.08), with Lambda greater in case of uGAS than in case of cGAS for 81 out of 151 metabolites (Wilcoxon paired samples t-test p=0.14). In our opinion, these results are convincingly demonstrating that the assumption that the uGAS and cGAS test statistics (under the null) follow the same distribution, and this distribution is a chi-squared with 1df, is valid.

2) The authors do not differentiate between random variables and their estimators in the manuscript.

Thank you for attracting out attention to this; we carefully revised manuscript and corrected our notation.

3) The approaches used could be described more clearly and the notation could be more consistent and intuitive. For example, for equation (1), it seems like beta_yg is simply the estimate of beta_g from the conditional model. This is generally denoted by hat{beta}_g - the authors could denote it by hat{beta^c}_g since they consider both the unconditional and conditional models; using non-standard notation makes the manuscript more difficult to follow. It also seems like beta_yc is in fact equal to rho_yc (under the assumption of zero mean and standard deviation of 1 for all the random variables) for the case of a single covariate. If so, this could be pointed out to the readers. The terminology is also not always consistent, eg "partial regression coefficients," "partial correlation coefficients," "partial coefficients of regression" etc. The "total observed correlation" is introduced in the discussion - presumably this is just the marginal correlation, but this term is not used elsewhere.

You are right. We now re-formulated text on p. 7 in order to avoid excessive indexes and to make the relation between different parameters and notations we use clearer. We also corrected the terminology we use throughout the manuscript.

4) The authors claim on page 7 that "Because the noise component [..] is always >= 1, any possible decrease in the ratio... is determined by the sign and magnitude of the term [..]. If this term is negative, there will always be an increase in power of the conditional analysis." However, the conditional analysis will necessarily estimate more parameters, using up more degrees of freedom. This becomes clear if one specifies that the t-test is used, for which the degrees of freedom decreases with the number of parameters. (In the discussion, the non-centrality parameter is mentioned - presumably for the t-test - but not the number of degrees of freedom.)

Here, we see two statements, which we will address separately.

First, we agree that “the conditional analysis will necessarily estimate more parameters, using up more degrees of freedom”. This, coupled with the fact that the univariate and the conditional models are hierarchical, is exactly the reason why the noise component is always >= 1. We now make this reasoning more clear in the text on p.7.

Second, you say that “This becomes clear if one specifies that the t-test is used, for which the degrees of freedom decreases with the number of parameters. (In the discussion, the non-centrality parameter is mentioned - presumably for the t-test - but not the number of degrees of freedom.)”. We are not using the t-test anywhere, but we rather use the Wald test that is distributed as chi-squared with one degree of freedom under the null. Again, we make this explicit on p. 7 now. The non-centrality parameter mentioned in Discussion relates to the non-centrality parameter of the Wald test under the alternative; we now change that to “log-ratio between the cGAS and uGAS tests” to avoid excessive notation.

Finally, while this is not explicitly stated, your first statement may suggest that the distribution of the log-ratio (that we use as an indicator of power advantage of the conditional vs. univariate model) may be shifted from zero. Both test statistics which are included in the log-ratio are distributed as chi-squared with 1df under the null, and our expectation is that on average, under the null, the log-ratio between them will be centered at zero (even if covariates c are significantly associated with outcome y). We empirically test this assumption by randomly sampling 10,000 SNPs for each trait and computing the log(T^2_c/T^2_u). Then, we tested whether this quantity is significantly different from zero, using the paired t-test and the Wilcoxon sign test. For each of the 151 traits, we found that the average log-ratio was close to zero (on average, the mean was 0.002; the proportion of log-ratios >0 was equal to 0.5006; the proportion of t-test having p<0.1 was 15/151=0.0993; the proportion of p<0.05 was 8/151 = 0.053).

5) The authors mention that one might be able to apply "a machine-learning approach that allows for differential shrinkage." It is unclear why they do not just apply something like a grouped LASSO (or even a regular LASSO that does not shrink the genotype) and compare the results?

This is an interesting suggestion; however, it is out of the scope of this work, which explored two network-based approaches, one of which learns from the data, and other is based on prior knowledge. Your suggestion would lead to another, not network-based, way

to analyze highly dimensional omics data; while this may be interesting, we see this as potentially separate big problem (e.g., developing a LASSO model that does not shrink some of the parameters, and that operates on summary level data, in our view, would be not trivial).

6) The portions on the chi-squared test are confusing. As written, it is somewhat confusing where this test was used (and why) and where the paired Wilcoxon was used (presumably the pairs represent the uGAS and cGAS models for each metabolite?)

Thank you for pointing this out. We used the ratio of the chi-squared tests to provide what we see as an "intuitive" measure of average gain (loss) of power, while testing whether this gain was statistically significant was done using non-parametric Wilcoxon test. We now make it clear the first time we apply this logic (lines 227-230 of previous version, L242-244 in current manuscript).

7) Having a reproducible workflow which includes both the data (summary-level KORA data) and the code would be very helpful to the reader.

As suggested by you and the editor we made available our scripts and summary data necessary for reproducing the results of work (in "push the button - get results" format) on the CodeOcean platform. The link to the workflow is https://codeocean.com/2018/04/02/a-network-based-conditional-genetic-association-analysis-of-the-human-metabolome/code .

8) a) It would be helpful to highlight locus FADS1 in Figure 1.

Done.

9) b) It would be helpful to use the notations introduced earlier in the caption of Figure 2, to make it easier to make the connections.

Done. Now we are using the term "partial regression coefficient" throughout the text.

Reviewer #2:
10) 1. It was not clear to me how different the proposed approach is from that of ref 15 which presents the conditional analysis method. As far as I can see, the basic idea is the same, but perhaps the way the covariates are selected is the key contribution here?

The central topic of the work [15] is the mathematical methodology allowing for conditional analysis based on summary-level data, and generalisation of this methodology to the case of multiple SNPs. Authors of [15] provide several numerical examples, but they do detail the implications and relevance of the model in general genetic and specific biological context. The also do not discuss the question of selection of covariates.

We use the same conditional model, as described by reference [15], and our summary-level-based implementation of the model and corresponding tests is very similar. We are not saying that "we build upon the work of [15]" because in fact we have developed the part of the method we use in parallel and in fact, published it first (see our biorxiv paper from Dec 2016 at https://www.biorxiv.org/content/early/2016/12/27/096982, while the paper [15] was published in May 2017). We take this approach one step further by analytical analysis and

identification of scenarios where one should expect gain or loss in power when compared with a model without covariates, and we discuss biological plausibility of different models; we apply the developed methods to real data and identify real examples of these models. The biological and genetic context aside, the main methodological difference between our current work and [15] is indeed, as you rightly noticed, the (network-based) way we select covariates for the model. We now make these similarities more explicit in Introduction.

11) 2. I find the general message of the paper rather unsurprising - that including other traits as covariates improves the model or its interpretation. Surely anyone would expect this to be the case? Perhaps the authors can make a clearer and more focused conclusion based on the novelty they are bringing to the work.

Thank you for this comment. Indeed, one of the general messages of our work is that “including other traits as covariates improves [the model’s] interpretation”, and indeed, this is expected. However, the statement that “including other traits as covariates improves model” is not a part of our message. Somewhat contradictory, and not entirely expected, our message in the context of testing of genetic effects is that adding other (biologically related) traits as covariates may increase or decrease the power, depending on interplay between the pleiotropic architecture of the locus being tested and the residual environmental and genetic factors. We now try to make our message more explicit in the Abstract and the Short Abstract.

12) 3. Lines 160-162. Please explain for readers not familiar with the approach, why Tc depends on beta_yg while Tu depends on rho_yg (not beta). Also the derivation of expressions for Tc in line 162 could do with being a little more explicit I think.

Thank you for this comment. We added necessary clarifications in text L160-171. Given the assumptions that all random variables are distributed with a mean of zero and a standard deviation of 1, the joint distribution of y, g, and c can be specified using a set of three correlation coefficients, rho_yg (correlation between the trait and the genotype), rho_cg (between the covariate and the trait), and rho_yc (between the trait and the covariate). In case of uGAS beta_y (denoted as beta_yg in previous version) is equivalent to the rho_yg. In case of conditional model beta_y is not equivalent to rho_yg, but beta_y =rho_yg - beta_c*rho_cg. For both T^2_c and T^2_u we used the Wald test of significance of deviation from zero.

We now make it explicit and introduce more explanation and a reference on page 7.

13) 4. The reasoning behind the discussion of the pleitropic component - lines 173-185 is not clear to me and I think could be made more explicit. For example, those not familiar with Medelian randomization studies may not follow the first sentence. Why is beta_yc 'mostly environmental'? Why would it be 'unexpected' for the genotype and environmental effects to be of different signs? This may be obvious to the authors, but I doubt to the general reader.

Thank you for these comments. To account for them, we added more explanation and details to the text of Results (L188-189) and also to the discussion (L384-400).

14) 5. Line 233 'As shown in figure 1, the ratio is determined primarily by the second (ie pleiotropic) term in Eq (2)'. Presumably the authors are drawing this conclusion from the

slope of the pleiotropic and noise regression lines in the figure? Please make this reasoning explicit.

Thank you for pointing this out. You right, we made this conclusion primarily from these slopes. We added explanation into the text (L247-248).

15) 6. Figure 1:
a. Please label the regression line going through the asterisks.

Done.

b. Caption: "on the y axis the asterisk corresponds to the log-ratio" - of what?

Of the log-ratio of cGAS and uGAS T2 statistics. We have now corrected it, thank you!

c. "The three dark green vertical lines". There are 4 dark green vertical lines.

Indeed. It was typo. We have now corrected it, thank you!

d. I am confused. There are 4 dark green vertical lines which are the "associations significant in cGAS but not uGAS". But table 1 shows only two associations of this type. Similarly there are 2 dark red lines which are associations "only significant in uGAS" but table 1 shows only 1 association of this type.

Green lines correspond to the SNP-trait pairs for which association was significant in cGAS but not in uGAS. It is true that we have two loci found to be significant only in cGAS (locus # 10 and #11 in table1), but also we have two SNP-trait pairs in each of the loci #1 and #2. These loci also have another SNP-traits pairs that were significant in uGAS, but for different traits. In the Figure, we show all of them. The same explains the number of red lines.

16) 7. For completeness it would be helpful to list the estimated pleiotropic and noise component terms in tables 1 & 2 for each locus.

Thank you for this suggestion. We have added corresponding column into the tables.

17) 8. Figure 2: I find this hard to follow:
a. "the first column below the diagonal line" What does this mean? I guess just the first column on the left?

Indeed, this is the first column; we have now corrected the sentence.

b. Do the areas of the squares and their colours represent different quantities?

No, they all correspond to the value of correlations. The area of a square is proportional to the absolute value of correlation (partial regression coefficient); the effect magnitude is also reflected by square's color (the scale provided at the bottom of the graph).

c. The text compares the conditional and unconditional analyses with respect to Fig 2. Are the results of the unconditional regression represented in the plot? If not, where?

Thank you for pointing this out. The matrix of correlations (above diagonal line) shows only the results of the univariate regression. We have arranged a new Supplementary Table (1C) summarizing univariate results for corresponding traits and SNPs for this figure.

18) 9. Line 298: In the GGM-cGAS study, the noise component was found to be larger than BN-cGAS. This seems to be opposite of what was expected?

No, the fact that noise component of GGM-cGAS is larger than the one of BN-cGAS was expected. Since GGM-cGAS had on average more covariates than BN-cGAS, and the GGM covariates were specifically selected to explain large proportion of the variance of the trait of interest, it is expected that the residual variance of the dependent variable will be smaller for GGM-cGAS than for BN-cGAS, leading to higher noise component of Eq. 2.

19) 10. The acylcarnitines are identified throughout just by their chain lengths (C10 etc). It would be helpful to clarify their chemical class on the plots/tables as well (since there are other classes present).

Thank you for this suggestion. We have added a column describing the chemical class of a metabolite into Supplementary Tables 1 and 2.

20) 11. Line 334-337 "We found no prior evidence…" Could these be new associations rather than false positives?

Taking into account that this association was not found in (much) bigger meta-analysis, it is rather unlikely that these are novel findings. We now indicate this in L349.

21) 12. Discussion, line 392: What is DEPICT?

Thank you for this question. We now decipher this abbreviation and provide the reference to the DEPICT software in Material and Methods L535: “To prioritize genes in associated regions, gene set enrichment, and tissue/cell-type enrichment analyses, we used DEPICT (Data-driven Expression-Prioritized Integration for Complex Traits) software”

22) 13. Methods line 490 typo: "separated by at least 500." 500 *what*?
Corrected to “at least 500 kb”, thank you!

Close
