# Supplementary material for: A network-based conditional genetic association analysis of the human metabolome
Source: Gigascience. 2018 Nov 29;7(12):giy137. doi: 10.1093/gigascience/giy137 (PMC6287100; doi:10.1093/gigascience/giy137)
Supplement: response_to_reviewer_comments_revision_1.pdf [file giy137_response_to_reviewer_comments_revision_1.pdf]

Author's Response To Reviewer Comments

Close

Dear Hans,

We would like to thank you and reviewers again for comments and suggestions. Please find attached our point-by-point answers below. We hope that you and the reviewers will find the revised manuscript suitable for publication.

While addressing the comments we have noticed that we have used SNP rs17112944 when comparing the components of GGM- and BN-cGAS (LL 315-323). However, in other parts of the manuscript we consider this SNP to be false-positive and thus exclude it from other computations. We now excluded this SNP from computations, making our story more coherent. These changes did not affect our conclusions.

Yours Sincerely. also on behalf of other authors,
prof. Yurii Aulchenko and dr. Yakov Tsepilov

Reviewer #1 report:

1) For Supplementary Table 1 - the ARCHIVE tab should be removed from the file, the row freeze should include the 11th SNP (row 17) in Supplementary Table 1A, the heading should be edited for Supplementary Table 1C to emphasize that it is specifically for 1C. It is also unclear to me where the p-values come from in Table S1C - they are not exactly the same as the uGAS p-values, but are very similar. I also think Figure 2 should reference this table rather than Table S1B.

Thank you for these suggestions. We made all necessary corrections in text and in Supplementary Tables. In tables ST1A-B and ST2 the freeze now includes only two first columns and the header. The reason of minor differences in p-values was the application of genomic control in S1A,B and S2 (see also comment #5).

2) Thank you for clarifying that the Wald test was used, assuming a chi-squared distribution. The reason I had mentioned the t-test is that it is generally more common to use the t-test for individual parameters in either simple or multivariable regression, as chi-squared tests rely on asymptotic properties (of the number of samples going to infinity) and are more commonly used in generalized linear regression models. The choice to use this test may thus be worth a short discussion.

We have used the Wald test statistic because this allowed us to analytically express the log-ratio between conditional and univariate tests (equations 1 and 2). We believe this is valid approximation because the typical number of samples in genetic association studies is thousands or orders or magnitude more, which we now mention on lines LL 168-170.

3) For Tables 1 and 2, it would be great if the noise/pleiotropic components were in different columns (easier to look at and potentially use in downstream analyses)

Thank you for this comment. Done.

4) Thank you for expanding the description of the model and changing the notation in the Results section. I think a few small edits should be made here:
- on line 161, rho_cg is the correlation between the covariate and the genotype, not the covariate and the trait
- on lines 165-167, should include "estimated" before "residual variance" and "partial correlation"

Corrected.

5.1) Thank you for making everything available through Code Ocean! The figures reproduce very well, however it would be helpful if all the main and supplementary tables were also available in the same format as they are in the manuscript.

Thank you for these suggestions. We now have implemented the full pipeline starting from clumping to the final supplementary tables. It should be noted, that some steps (like comparison with Tsepilov et.al., 2015 and Draisma et.al., 2016) were done manually and are not implemented in CodeOcean.

5.2) I also wasn't sure where the uGAS output was to be found.

The uGAS output could be found here: CodeOcean workspace/Data/uGWAS
Thanks to your comment, we now updated the README file to make the navigation more clear. Note that uGAS is denoted as uGWAS in CodeOcean.

5.3) Additionally, I found some small discrepancies with the cGAS p-values, for example in Table 2, the p-value for the association of rs2286963 with C9 is 7.41E-73 whereas in the Code Ocean result it is 1.53E-73.

The reason for these discrepancies in p-values was the application of genomic control in S1A, S1B, S2. To all supplementary tables we have added new columns that list p-values before and after genomic control.

Close
