# Supplementary material for: A network-based conditional genetic association analysis of the human metabolome
Source: Gigascience. 2018 Nov 29;7(12):giy137. doi: 10.1093/gigascience/giy137 (PMC6287100; doi:10.1093/gigascience/giy137)
Supplement: response_to_reviewer_comments_revision_2.pdf [file giy137_response_to_reviewer_comments_revision_2.pdf]

Author's Response To Reviewer Comments

Close

Dear Hans,

Please find attached our revised manuscript (in .docx format). We have added citations to "Availability of Data and Materials" section and to reference list. We also have removed highlighting in red and changed affiliation #11.

Yours Sincerely. also on behalf of other authors,
prof. Yurii Aulchenko and dr. Yakov Tsepilov

Close
