# Supplementary material for: A network-based conditional genetic association analysis of the human metabolome
Source: Gigascience. 2018 Nov 29;7(12):giy137. doi: 10.1093/gigascience/giy137 (PMC6287100; doi:10.1093/gigascience/giy137)
Supplement: reviewer_1_report_(original_submission) -- Simina Boca — 1/22/2018 Reviewed [file giy137_reviewer_1_report_(original_submission).pdf]

Reviewer Report

Title: A network-based conditional genetic association analysis of the human metabolome

Version: Original Submission **Date:** 1/22/2018

Reviewer name Simina Boca

Reviewer Comments to Author:

This manuscript discusses a metabolomics GWAS analysis, namely a GWAS where the outcomes/traits of interest are metabolites. It discusses the use of different models for looking at the associations between metabolites and genotypes. Thus, in this scenario, one may either consider a model where the metabolic trait is regressed only on the genotype ("unconditional model" = uGAS) or a model that additionally also includes other metabolites as covariates ("conditional model" = cGAS). The metabolites included as covariates can be selected in 2 ways: either by using known biochemical networks (BN) or by using a data-driven approach with Gaussian graphical models (GGM.) Thus, the uGAS represents simple linear regression models in the context of GWAS, whereas the cGAS represents multiple linear regression, with a variable selection step.

In general, I find that this paper could be clearer and more rigorous from a statistical point of view. For example:
1) One major issue I see is the way the authors claim that a cGAS would usually have higher power. In general, power is defined in relation to a specific null hypothesis and the null hypothesis is different for the uGAS versus the cGAS. For example, the higher power may be at the cost of a higher type I error/FWER. Furthermore, all these claims are made based on a single data analysis, whereas more convincing and illustrative arguments generally would also include simulations where the true data-generating mechanisms is known - for example, if the data follows the uGAS models, how does using the cGAS impact the type I error/FWER as well as the power?
2) The authors do not differentiate between random variables and their estimators in the manuscript.
3) The approaches used could be described more clearly and the notation could be more consistent and intuitive. For example, for equation (1), it seems like beta_yg is simply the estimate of beta_g from the conditional model. This is generally denoted by hat{beta}_g - the authors could denote it by hat{beta^c}_g since they consider both the unconditional and conditional models; using non-standard notation makes the manuscript more difficult to follow. It also seems like beta_yc is in fact equal to rho_yc (under the assumption of zero mean and standard deviation of 1 for all the random variables) for the case of a single covariate. If so, this could be pointed out to the readers. The terminology is also not always consistent, eg "partial regression coefficients," "partial correlation coefficients," "partial coefficients of regression" etc. The "total observed correlation" is introduced in the discussion - presumably this is just the marginal correlation, but this term is not used elsewhere.
4) The authors claim on page 7 that "Because the noise component [..] is always >= 1, any possible decrease in the ratio... is determined by the sign and magnitude of the term [..]. If this term is negative, there will always be an increase in power of the conditional analysis." However, the conditional analysis will necessarily estimate more parameters, using up more degrees of freedom. This becomes clear if one specifies that the t-test is used, for which the degrees of freedom decreases with the number of parameters. (In the discussion, the non-centrality parameter is mentioned - presumably for the t-test - but not the number of degrees of freedom.)
5) The authors mention that one might be able to apply "a machine-learning approach that allows for differential shrinkage." It is unclear why they do not just apply something like a grouped LASSO (or even a regular LASSO that does not shrink the genotype) and compare the results?
6) The portions on the chi-squared test are confusing. As written, it is somewhat confusing where this test was used (and why) and where the paired Wilcoxon was used (presumably the pairs represent the uGAS and

cGAS models for each metabolite?)
7) Having a reproducible workflow which includes both the data (summary-level KORA data) and the code would be very helpful to the reader.

Minor comments:
a) It would be helpful to highlight locus FADS1 in Figure 1.
b) It would be helpful to use the notations introduced earlier in the caption of Figure 2, to make it easier to make the connections.

Level of Interest

Please indicate how interesting you found the manuscript: An article of importance in its field

Quality of Written English

Please indicate the quality of language in the manuscript: Needs some language corrections before being published

Declaration of Competing Interests

Please complete a declaration of competing interests, considering the following questions:

- Have you in the past five years received reimbursements, fees, funding, or salary from an organisation that may in any way gain or lose financially from the publication of this manuscript, either now or in the future?
- Do you hold any stocks or shares in an organisation that may in any way gain or lose financially from the publication of this manuscript, either now or in the future?
- Do you hold or are you currently applying for any patents relating to the content of the manuscript?
- Have you received reimbursements, fees, funding, or salary from an organization that holds or has applied for patents relating to the content of the manuscript?
- Do you have any other financial competing interests?
- Do you have any non-financial competing interests in relation to this paper?

If you can answer no to all of the above, write 'I declare that I have no competing interests' below. If your reply is yes to any, please give details below.

I declare that I have no competing interests

I agree to the open peer review policy of the journal. I understand that my name will be included on my report to the authors and, if the manuscript is accepted for publication, my named report including any attachments I upload will be posted on the website along with the authors' responses. I agree for my report to be made available under an Open Access Creative Commons CC-BY license (http://creativecommons.org/licenses/by/4.0/). I understand that any comments which I do not wish to

be included in my named report can be included as confidential comments to the editors, which will not be published.

I agree to the open peer review policy of the journal

To further support our reviewers, we have joined with Publons, where you can gain additional credit to further highlight your hard work (see: https://publons.com/journal/530/gigascience). On publication of this paper, your review will be automatically added to Publons, you can then choose whether or not to claim your Publons credit. I understand this statement. Yes
