# Supplementary material for: A network-based conditional genetic association analysis of the human metabolome
Source: Gigascience. 2018 Nov 29;7(12):giy137. doi: 10.1093/gigascience/giy137 (PMC6287100; doi:10.1093/gigascience/giy137)
Supplement: reviewer_1_report_revision_1 -- Simina Boca — 5/25/2018 Reviewed [file giy137_reviewer_1_report_revision_1.pdf]

Reviewer Report

Title: A network-based conditional genetic association analysis of the human metabolome

Version: Revision 1 Date: 5/25/2018

Reviewer name Simina Boca

Reviewer Comments to Author:

My concerns have for the most part been resolved. A few minor notes:

1) For Supplementary Table 1 - the ARCHIVE tab should be removed from the file, the row freeze should include the 11th SNP (row 17) in Supplementary Table 1A, the heading should be edited for Supplementary Table 1C to emphasize that it is specifically for 1C. It is also unclear to me where the p-values come from in Table S1C - they are not exactly the same as the uGAS p-values, but are very similar. I also think Figure 2 should reference this table rather than Table S1B.
2) Thank you for clarifying that the Wald test was used, assuming a chi-squared distribution. The reason I had mentioned the t-test is that it is generally more common to use the t-test for individual parameters in either simple or multivariable regression, as chi-squared tests rely on asymptotic properties (of the number of samples going to infinity) and are more commonly used in generalized linear regression models. The choice to use this test may thus be worth a short discussion.
3) For Tables 1 and 2, it would be great if the noise/pleiotropic components were in different columns (easier to look at and potentially use in downstream analyses)
4) Thank you for expanding the description of the model and changing the notation in the Results section. I think a few small edits should be made here:
- on line 161, rho_cg is the correlation between the covariate and the genotype, not the covariate and the trait
- on lines 165-167, should include "estimated" before "residual variance" and "partial correlation"
5) Thank you for making everything available through Code Ocean! The figures reproduce very well, however it would be helpful if all the main and supplementary tables were also available in the same format as they are in the manuscript. I also wasn't sure where the uGAS output was to be found. Additionally, I found some small discrepancies with the cGAS p-values, for example in Table 2, the p-value for the association of rs2286963 with C9 is 7.41E-73 whereas in the Code Ocean result it is 1.53E-73.

Level of Interest

Please indicate how interesting you found the manuscript: Choose an item.

Quality of Written English

Please indicate the quality of language in the manuscript: Choose an item.

Declaration of Competing Interests

Please complete a declaration of competing interests, considering the following questions:

- Have you in the past five years received reimbursements, fees, funding, or salary from an organisation that may in any way gain or lose financially from the publication of this manuscript, either now or in the future?

- Do you hold any stocks or shares in an organisation that may in any way gain or lose financially from the publication of this manuscript, either now or in the future?
- Do you hold or are you currently applying for any patents relating to the content of the manuscript?
- Have you received reimbursements, fees, funding, or salary from an organization that holds or has applied for patents relating to the content of the manuscript?
- Do you have any other financial competing interests?
- Do you have any non-financial competing interests in relation to this paper?

If you can answer no to all of the above, write 'I declare that I have no competing interests' below. If your reply is yes to any, please give details below.

I declare that I have no competing interests

I agree to the open peer review policy of the journal. I understand that my name will be included on my report to the authors and, if the manuscript is accepted for publication, my named report including any attachments I upload will be posted on the website along with the authors' responses. I agree for my report to be made available under an Open Access Creative Commons CC-BY license (http://creativecommons.org/licenses/by/4.0/). I understand that any comments which I do not wish to be included in my named report can be included as confidential comments to the editors, which will not be published.

I agree to the open peer review policy of the journal

To further support our reviewers, we have joined with Publons, where you can gain additional credit to further highlight your hard work (see: https://publons.com/journal/530/gigascience). On publication of this paper, your review will be automatically added to Publons, you can then choose whether or not to claim your Publons credit. I understand this statement. Yes
