# Supplementary material for: A network-based conditional genetic association analysis of the human metabolome
Source: Gigascience. 2018 Nov 29;7(12):giy137. doi: 10.1093/gigascience/giy137 (PMC6287100; doi:10.1093/gigascience/giy137)
Supplement: reviewer_2_report_(original_submission) -- Tim Ebbels — 2/2/2018 Reviewed [file giy137_reviewer_2_report_(original_submission).pdf]

Reviewer Report

Title: **A network-based conditional genetic association analysis of the human metabolome**

Version: **Original Submission** **Date:** 2/2/2018

Reviewer name **Tim Ebbels**

Reviewer Comments to Author:

This paper presents an approach for analysing associations between genomic variants and multiple phenotypes (traits) by conditioning the models on a subset of the traits. The authors argue that this 'trait centric' approach is in contrast to the conventional approach which focuses on the effect of a gene on multiple traits. They demonstrate the method on a set of metabolomics data, selecting the covariate traits either according to known biological pathways, or via a correlation network.

The work is interesting and potentially useful. However, I found some parts of the manuscript hard to follow. I think the text would benefit from a clearer explanation of the motivation for the new approach, as well as the method itself, accessible to readers outside the immediate statistical genetics community. I list some specific comments below.

1. It was not clear to me how different the proposed approach is from that of ref 15 which presents the conditional analysis method. As far as I can see, the basic idea is the same, but perhaps the way the covariates are selected is the key contribution here?
2. I find the general message of the paper rather unsurprising - that including other traits as covariates improves the model or its interpretation. Surely anyone would expect this to be the case? Perhaps the authors can make a clearer and more focused conclusion based on the novelty they are bringing to the work.
3. Lines 160-162. Please explain for readers not familiar with the approach, why Tc depends on beta_yg while Tu depends on rho_yg (not beta). Also the derivation of expressions for Tc in line 162 could do with being a little more explicit I think.
4. The reasoning behind the discussion of the pleitropic component - lines 173-185 is not clear to me and I think could be made more explicit. For example, those not familiar with Medelian randomization studies may not follow the first sentence. Why is beta_yc 'mostly environmental'? Why would it be 'unexpected' for the genotype and environmental effects to be of different signs? This may be obvious to the authors, but I doubt to the general reader.
5. Line 233 'As shown in figure 1, the ratio is determined primarily by the second (ie pleiotropic) term in Eq (2)'. Presumably the authors are drawing this conclusion from the slope of the pleiotropic and noise regression lines in the figure? Please make this reasoning explicit.
6. Figure 1:
a. Please label the regression line going through the asterisks.
b. Caption: "on the y axis the asterisk corresponds to the log-ratio" - of what?
c. "The three dark green vertical lines". There are 4 dark green vertical lines.
d. I am confused. There are 4 dark green vertical lines which are the "associations significant in cGAS but not uGAS". But table 1 shows only two associations of this type. Similarly there are 2 dark red lines which are associations "only significant in uGAS" but table 1 shows only 1 association of this type.
7. For completeness it would be helpful to list the estimated pleiotropic and noise component terms in tables 1 & 2 for each locus.
8. Figure 2: I find this hard to follow:
a. "the first column below the diagonal line" What does this mean? I guess just the first column on the left?
b. Do the areas of the squares and their colours represent different quantities?
c. The text compares the conditional and unconditional analyses with respect to Fig 2. Are the results of the

unconditional regression represented in the plot? If not, where?
9. Line 298: In the GGM-cGAS study, the noise component was found to be larger than BN-cGAS. This seems to be opposite of what was expected?
10. The acylcarnitines are identified throughout just by their chain lengths (C10 etc). It would be helpful to clarify their chemical class on the plots/tables as well (since there are other classes present).
11. Line 334-337 "We found no prior evidence…" Could these be new associations rather than false positives?
12. Discussion, line 392: What is DEPICT?
13. Methods line 490 typo: "separated by at least 500." 500 *what*?

Level of Interest

Please indicate how interesting you found the manuscript: An article whose findings are important to those with closely related research interests

Quality of Written English

Please indicate the quality of language in the manuscript: Acceptable

Declaration of Competing Interests

Please complete a declaration of competing interests, considering the following questions:

- Have you in the past five years received reimbursements, fees, funding, or salary from an organisation that may in any way gain or lose financially from the publication of this manuscript, either now or in the future?
- Do you hold any stocks or shares in an organisation that may in any way gain or lose financially from the publication of this manuscript, either now or in the future?
- Do you hold or are you currently applying for any patents relating to the content of the manuscript?
- Have you received reimbursements, fees, funding, or salary from an organization that holds or has applied for patents relating to the content of the manuscript?
- Do you have any other financial competing interests?
- Do you have any non-financial competing interests in relation to this paper?

If you can answer no to all of the above, write 'I declare that I have no competing interests' below. If your reply is yes to any, please give details below.

I declare that I have no competing interests

I agree to the open peer review policy of the journal. I understand that my name will be included on my report to the authors and, if the manuscript is accepted for publication, my named report including any attachments I upload will be posted on the website along with the authors' responses. I agree for my report to be made available under an Open Access Creative Commons CC-BY license (http://creativecommons.org/licenses/by/4.0/). I understand that any comments which I do not wish to

be included in my named report can be included as confidential comments to the editors, which will not be published.

I agree to the open peer review policy of the journal

To further support our reviewers, we have joined with Publons, where you can gain additional credit to further highlight your hard work (see: https://publons.com/journal/530/gigascience). On publication of this paper, your review will be automatically added to Publons, you can then choose whether or not to claim your Publons credit. I understand this statement. Yes
